# Application of the EU-SILC 2011 data module “intergenerational transmission of disadvantage” to robust analysis of inequality of opportunity

**DOI:** 10.1016/j.dib.2019.104301

**Published:** 2019-07-23

**Authors:** Francesco Andreoli, Alessio Fusco

**Affiliations:** a Department of Economics, University of Verona, Via Cantarane 24, 37129, Verona, Italy; b Luxembourg Institute of Socio-Economic Research – LISER, 10 Porte des Sciences, L-4366, Esch-sur-Alzette, Luxembourg

**Keywords:** Inequality, Survey data, Intergenerational, Cohort, Earnings, Gap curves, Europe

## Abstract

This data article describes the original data, the sample selection process and the variables used in Andreoli and Fusco (Andreoli and Fusco, 2019) to estimate gap curves for a sample of European countries. Raw data are from 2011 roaster of EU-SILC, cross-sectional sample of module “intergenerational transmission of disadvantage”. This article reports descriptive statistics of the using sample. It also discusses the algorithm adopted to estimate the main effects and details the content of additional Stata files stored on the online repository. These additional files contain raw estimates from bootstrapped samples, which form the basis for estimating gap curves and their variance-covariance matrices. The data article also reports representations of gap curves for all 16 selected countries.

**Table:** Specifications table

Subject	Economics
Specific subject area	Public economics, welfare economics, inequality analysis, distribution methods, inference
Type of data	Table Figure Raw (sample) data
How data were acquired	Access to EU-SILC 2011 wave granted within the NETSILC2 collaborative network. Data available from Eurostat upon request, see Microdata Access Workflow Tool.
Data format	Raw data (not uploaded on the server), anonymized sample used in the analysis (uploaded), bootstrapped estimators (uploaded) are all in Stata format.
Parameters for data collection	Survey data collected by European National Statistics Offices on behalf of Eurostat. Collection is based on households, individuals and houses registers. The using sample is representative of the population aged 18 to 80 living in selected European countries in 2010.
Description of data collection	Primary and Secondary survey units are randomized from registers. PSU defines the geographic are of stratification, SSU defines the households/individuals, for which a representative sample is collected. Information needed for EU-SILC can be extracted either from registers or collected from interviews. As for the interviews, there are four different ways to collect the data: Paper-Assisted Personal Interview (PAPI), Computer-Assisted Personal Interview (CAPI), Computer-Assisted Telephone Interview (CATI) and Self-administrated questionnaire. Data are collected with one-shot survey fieldwork which extends over less than four consecutive months. The lag between income reference period and fieldwork is limited to eight months.
Data source location	Esch-sur-Alzette, Luxembourg, Data Centre of the Luxembourg Institute of Socio-Economic Research.
Data accessibility	Raw data are not available on the public repository. They can be accessed through Eurostat upon request, see Microdata Access Workflow Tool. An anonymized using sample is made available: Repository name: Mendeley Data Data identification number: 10.17632/4fyym7dhxg.1 Direct URL to data: https://data.mendeley.com/datasets/4fyym7dhxg/1
Related research article	Francesco Andreoli, Alessio Fusco Robust cross-country analysis of inequality of opportunity Economics Letters https://doi.org/10.1016/j.econlet.2019.06.005

**Table:** 

**Value of the data** • EU-SILC data represent the baseline survey introduced by the European Commission and managed by Eurostat to monitor and compare standard of living across European countries. • Data are highly harmonized across countries, and collected by central statistical institutes. This guarantees a high degree of comparability of countries in terms of the main variables we consider to define earnings opportunities and parental circumstances. • Data are available free of charge in selected institutions in Europe (such as LISER). Users can apply for a visiting scheme which grants resources (material and knowledge-based) to the users of these data.

## 1 Data

The raw data are from the *European Union - Statistics on Income and Living Conditions* (EU-SILC) 2011 module on intergenerational transmission of disadvantage, where measures of parental background for a sufficiently large number of respondents are available. This module provides repeated cross-sectional information on the socioeconomic background of origin of the individuals interviewed in EU-SILC, along with standard relevant measures of labour market outcomes. In particular, the 2011 module contains retrospective information about the parental background experienced by the respondents when aged between 12 and 16 (see Atkinson et al. [3] for pros and cons of retrospective data). This unique base provides (to a large extent) comparable data allowing similar definitions for variables measuring outcome and circumstances across countries and time.

Base on raw EU-SILC 2011 module data (cross-section) data, this article extrapolates information for a subset of 16 countries: Austria (AT), Belgium (BE), Germany (DE), Estonia (EE), Finland (FI), Hungary (HU), Ireland (IE), Iceland (IS), Lithuania (LT), Luxembourg (LU), the Netherlands (NL), Norway (NO), Poland (PL), Sweden (SE), Slovakia (SK) and the United Kingdom (UK).

Sample selection process is based on males, aged between 30 and 50 who worked full time as an employee for at least 7 months in the income reference period. In addition, individuals who declared that they were living in another private household, foster home, collective household or institution were excluded. Following Raitano and Vona [4], intergenerational module weights are applied. The running sample that is used to produce Table 1 and Fig. 1 in [1] is made of 41,533 male respondents for which we observe circumstances, earnings and demographics (age in years and a categorical variable for being married). Descriptive statistics of the distribution of those variables are reported in Table 1 below. The data files are collected in the *example_econletters.dta* file in Stata format (optimized for Stata 13) available on the online repository.

**Table 1:** Summary statistics of running sample.

Country	N	Types			Earnings				Age	Married
		High	Medium	Low	All	High	Medium	Low		
AT	2887	0.10	0.43	0.48	37,320	49,367	39,829	32,604	40.4	0.69
BE	2446	0.19	0.23	0.57	38,788	54,702	37,742	33,792	40.1	0.65
DE	5345	0.30	0.58	0.11	41,444	44,228	40,642	38,108	41.4	0.75
EE	1777	0.18	0.43	0.40	12,966	17,494	13,398	10,508	40.4	0.64
FI	1949	0.21	0.22	0.56	31,245	41,842	30,229	27,627	40.4	0.61
HU	3825	0.10	0.36	0.54	11,548	19,096	12,506	9,476	39.8	0.69
IE	1122	0.14	0.22	0.65	40,408	52,155	48,067	35,358	40.2	0.74
IS	835	0.14	0.50	0.35	35,873	40,840	37,189	31,950	40.1	0.59
LT	1716	0.11	0.29	0.60	9,546	13,485	10,424	8,426	41.4	0.87
LU	2883	0.13	0.31	0.56	48,562	67,307	57,617	39,039	39.7	0.69
NL	2310	0.21	0.27	0.52	44,900	52,415	48,198	40,212	40.1	0.64
NO	1622	0.28	0.43	0.29	40,774	47,395	39,119	36,872	40.2	0.57
PL	5805	0.06	0.49	0.45	13,641	19,894	14,599	11,726	39.9	0.86
SE	1349	0.16	0.24	0.60	30,673	39,868	32,158	27,583	39.7	0.48
SK	2977	0.10	0.60	0.31	10,809	15,002	10,699	9,702	40.3	0.80
UK	2685	0.17	0.25	0.58	43,383	57,191	46,342	38,034	40.4	0.66

Total	41533	0.16	0.40	0.44	29,447	41,888	29,187	25,230	40.3	0.71

**Fig. 1:**
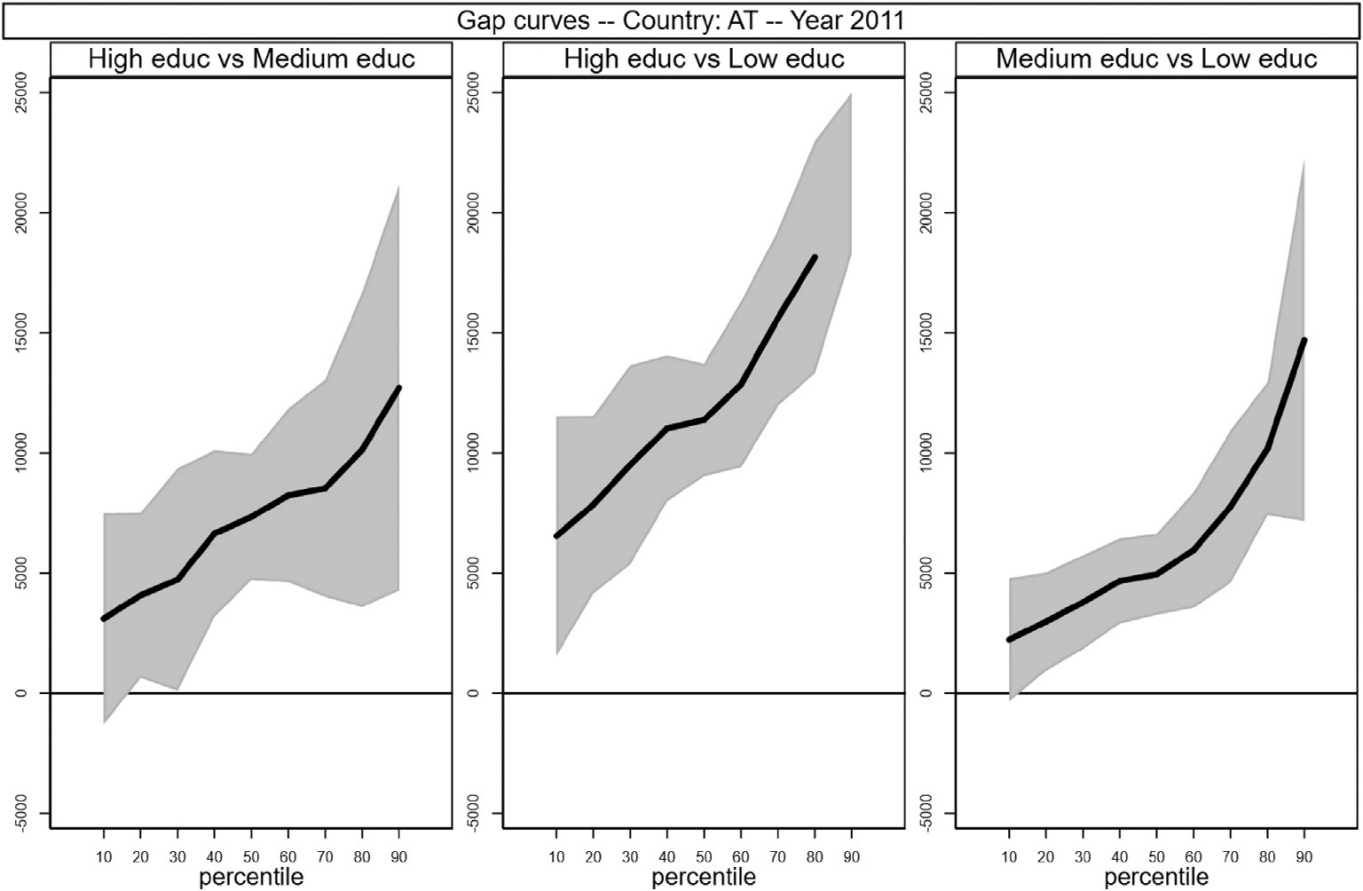
Gap curves for Austria.

Fig. 1, Fig. 2, Fig. 3, Fig. 4, Fig. 5, Fig. 6, Fig. 7, Fig. 8, Fig. 9, Fig. 10, Fig. 11, Fig. 12, Fig. 13, Fig. 14, Fig. 15, Fig. 16 in this article (see also [1]) are obtained from circumstances and earnings variables created from the raw data.

**Fig. 2:**
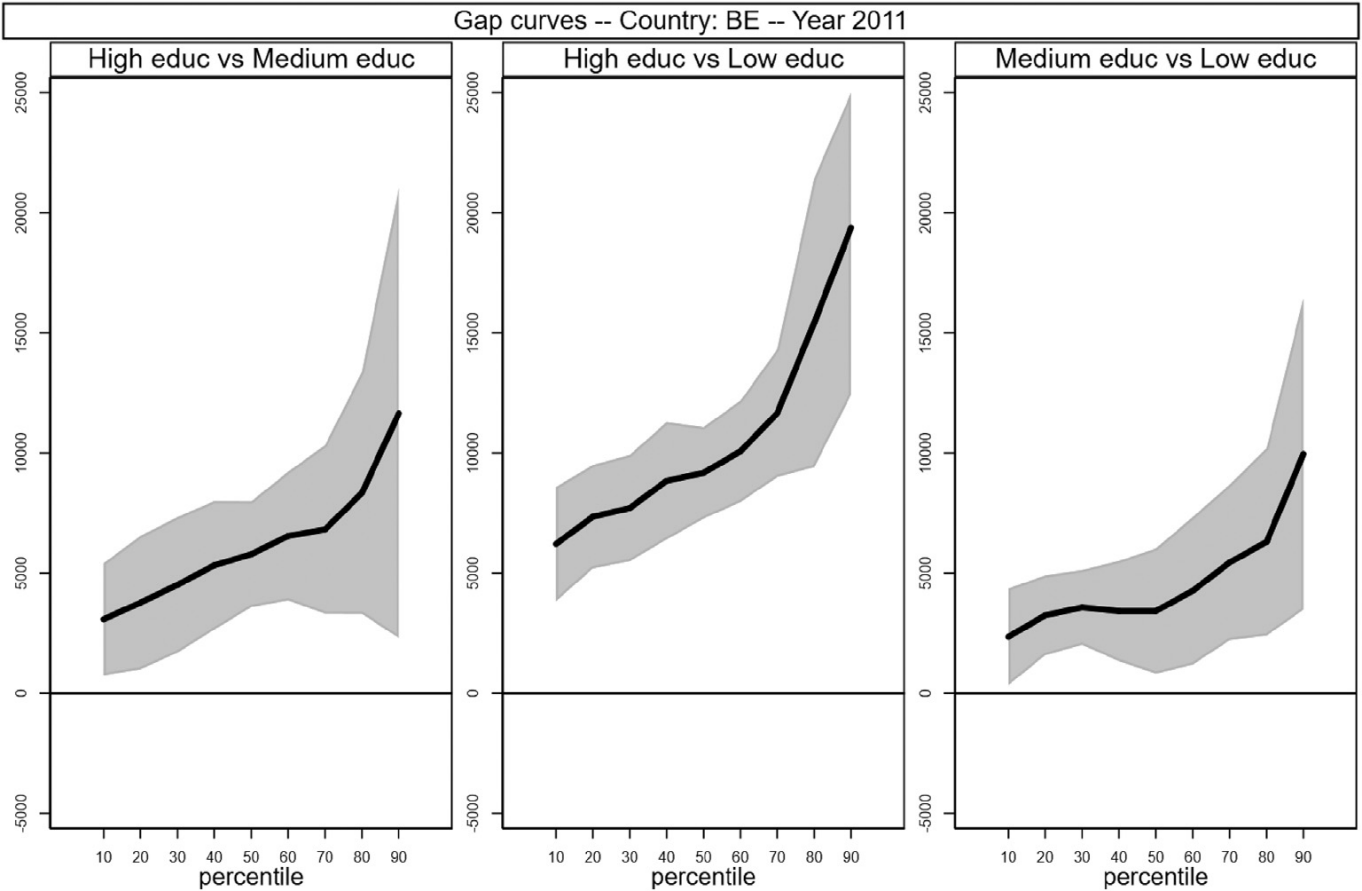
Gap curves for Belgium.

**Fig. 3:**
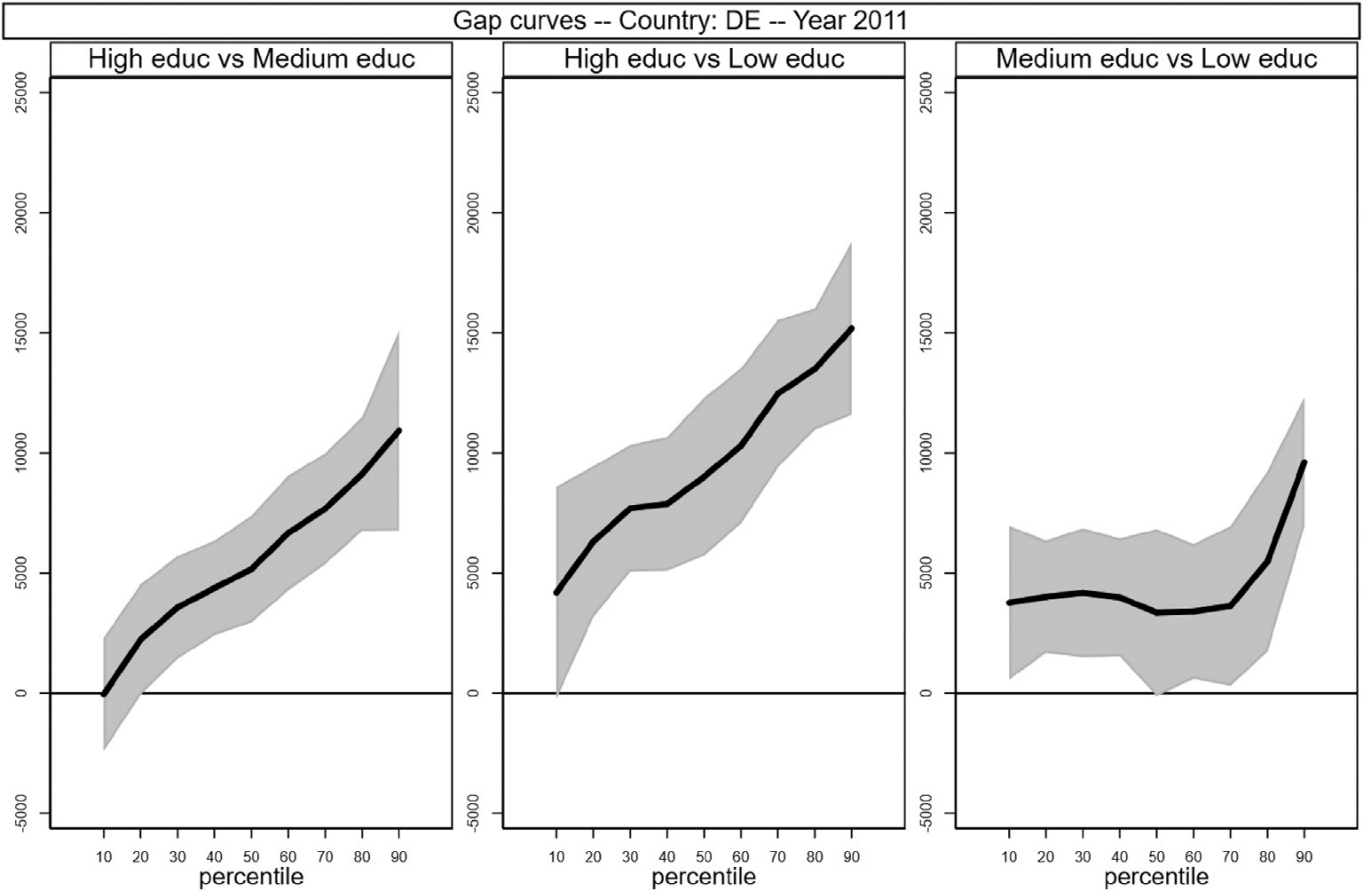
Gap curves for Germany.

**Fig. 4:**
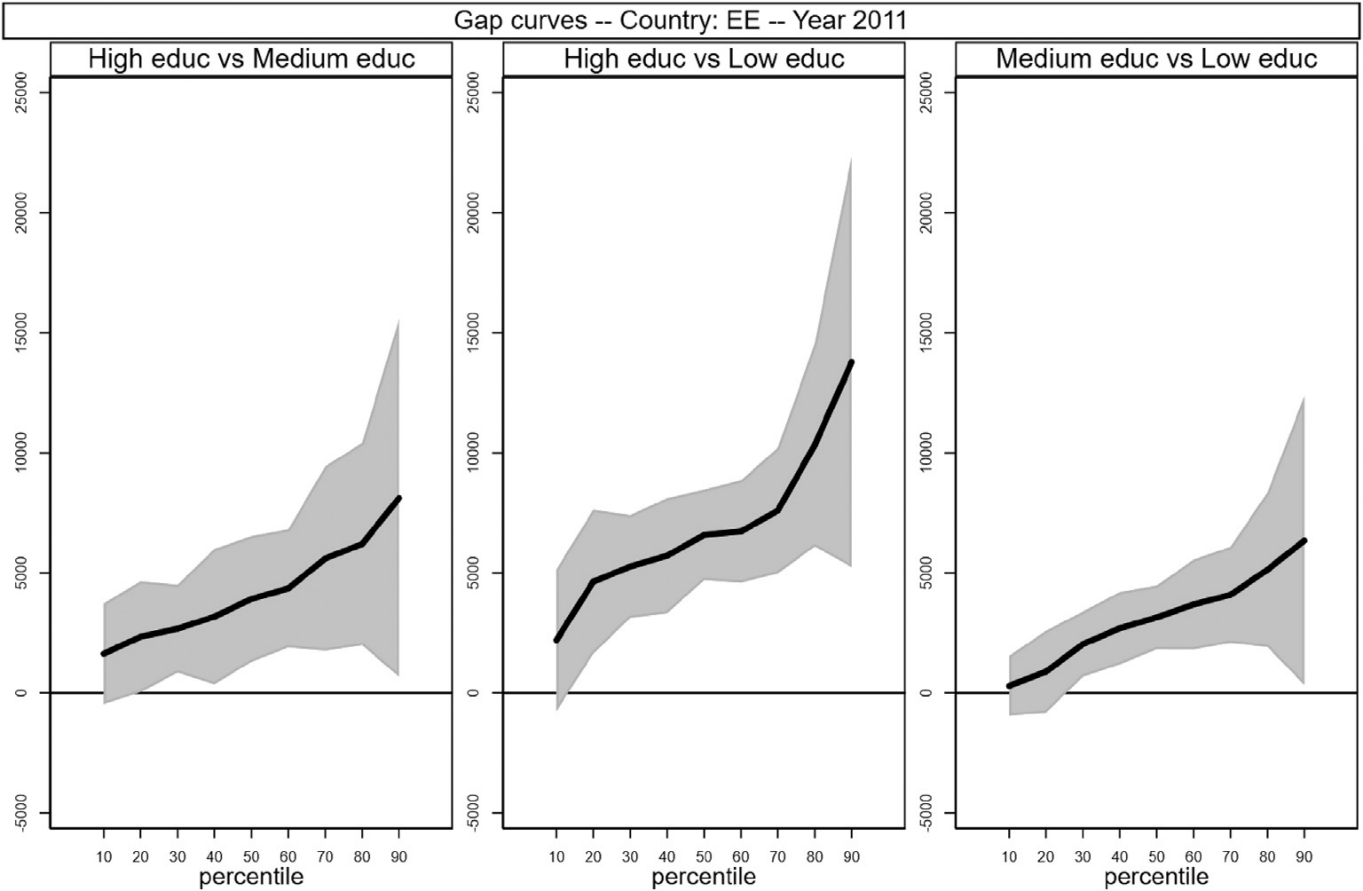
Gap curves for Estonia.

**Fig. 5:**
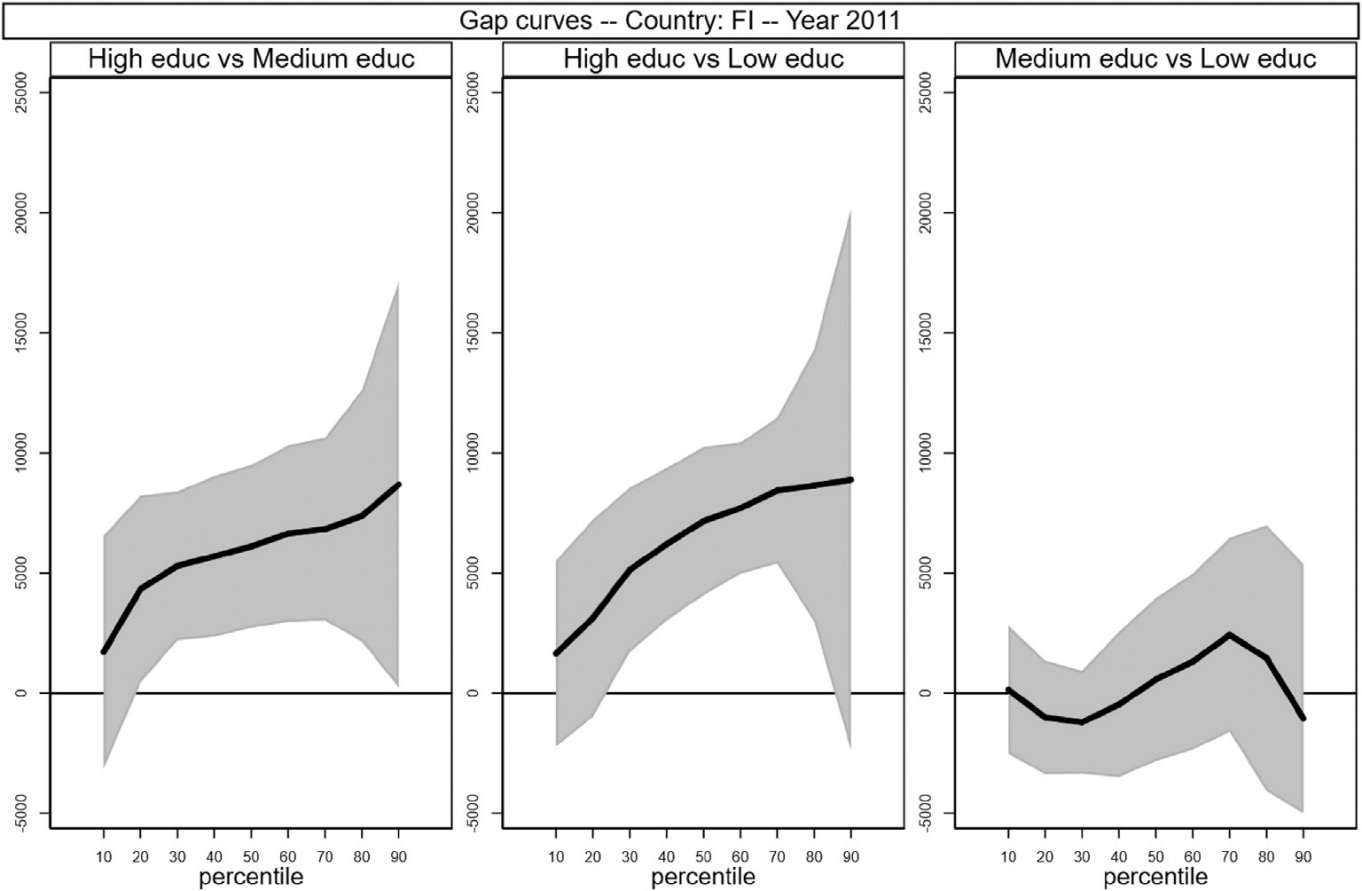
Gap curves for Finland.

**Fig. 6:**
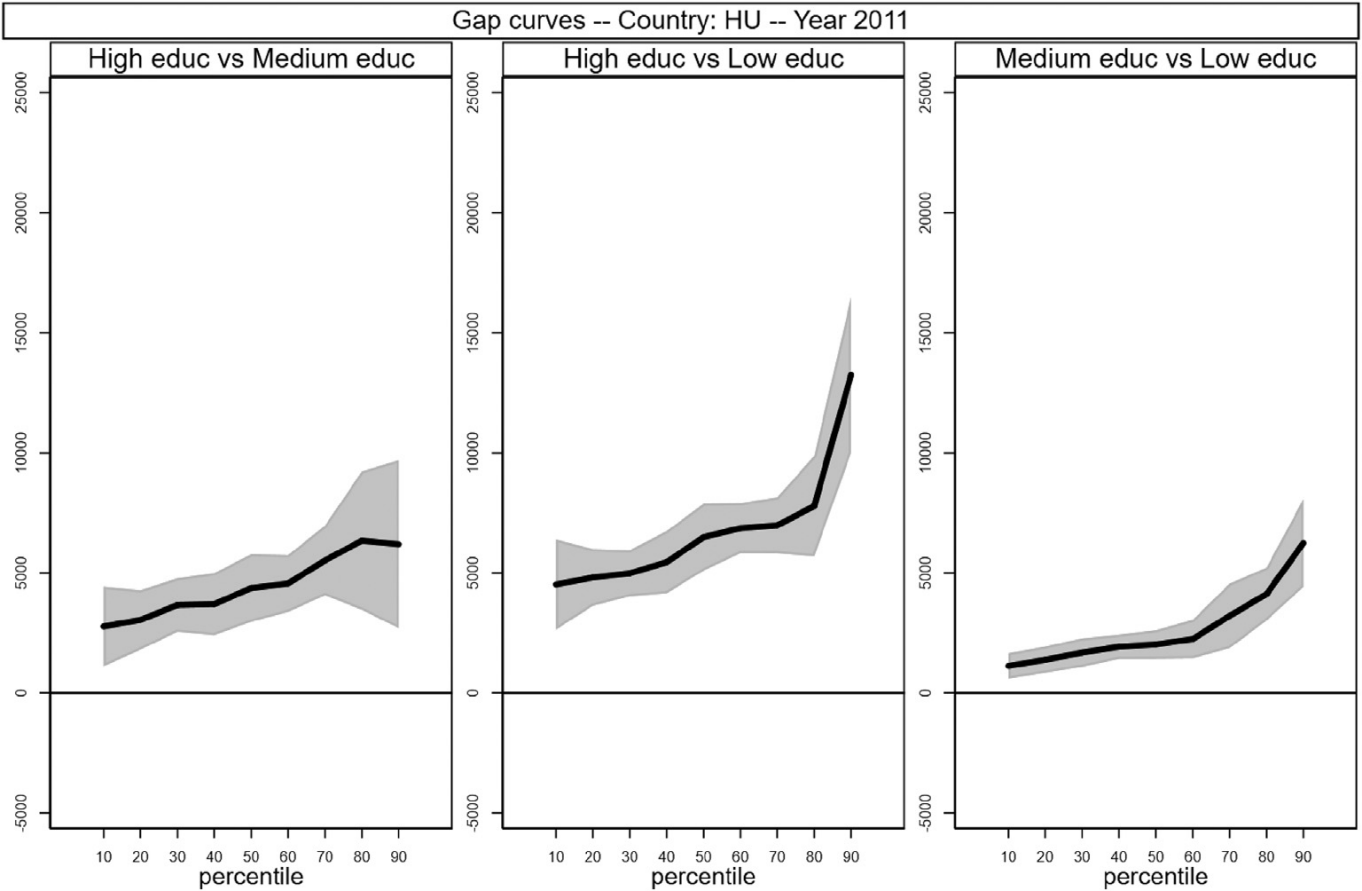
Gap curves for Hungary.

**Fig. 7:**
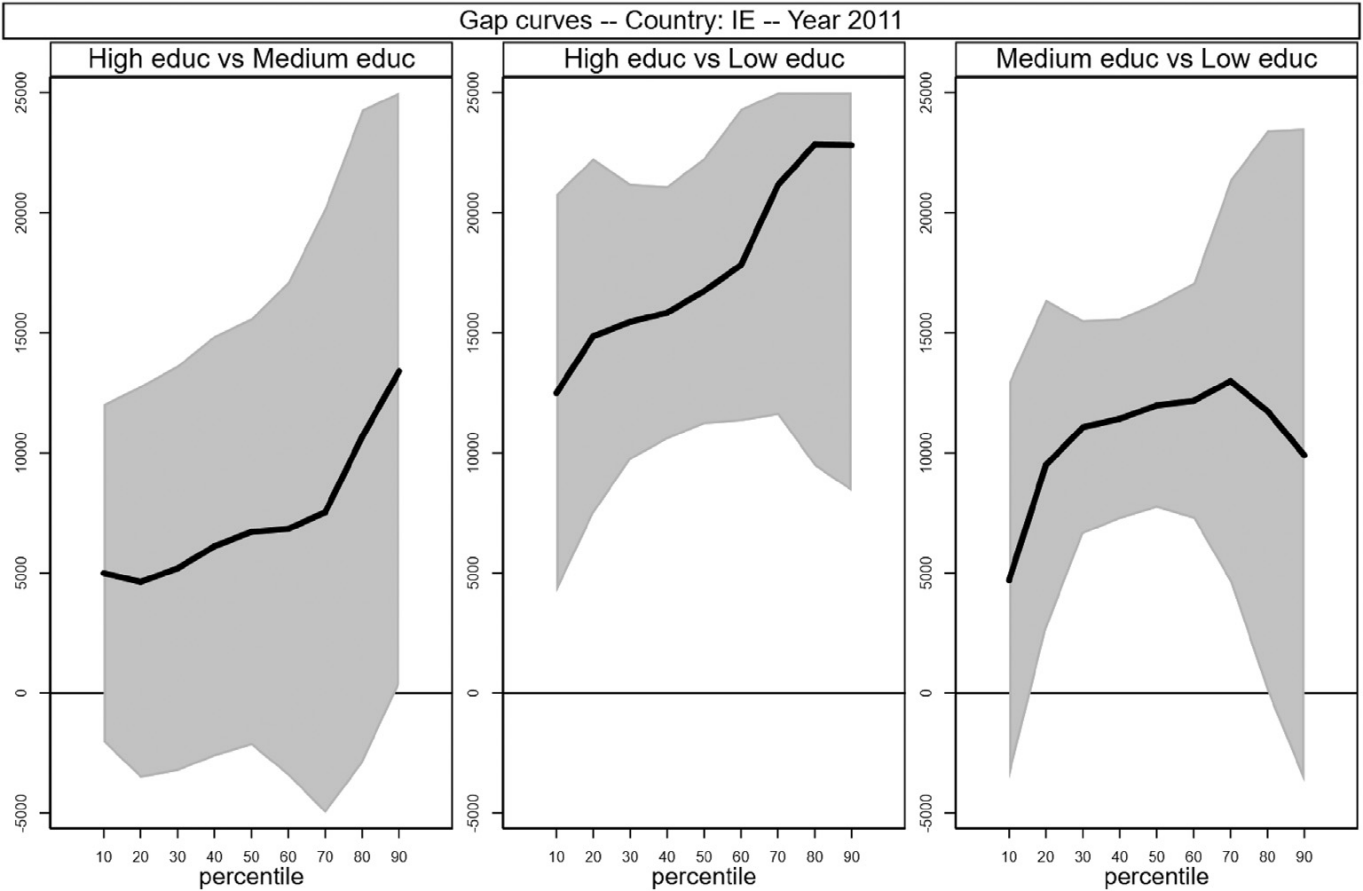
Gap curves for Ireland.

**Fig. 8:**
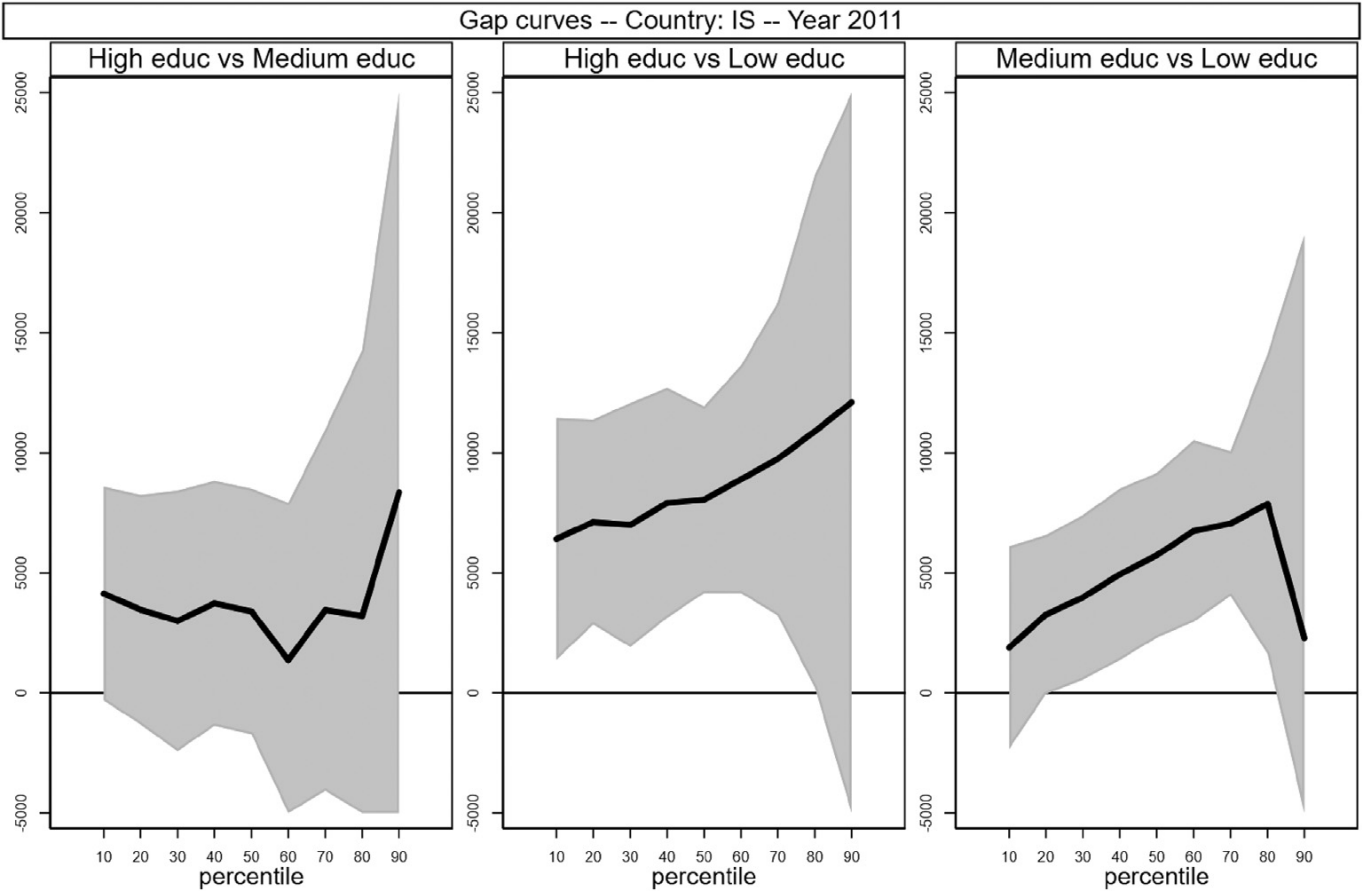
Gap curves for Iceland.

**Fig. 9:**
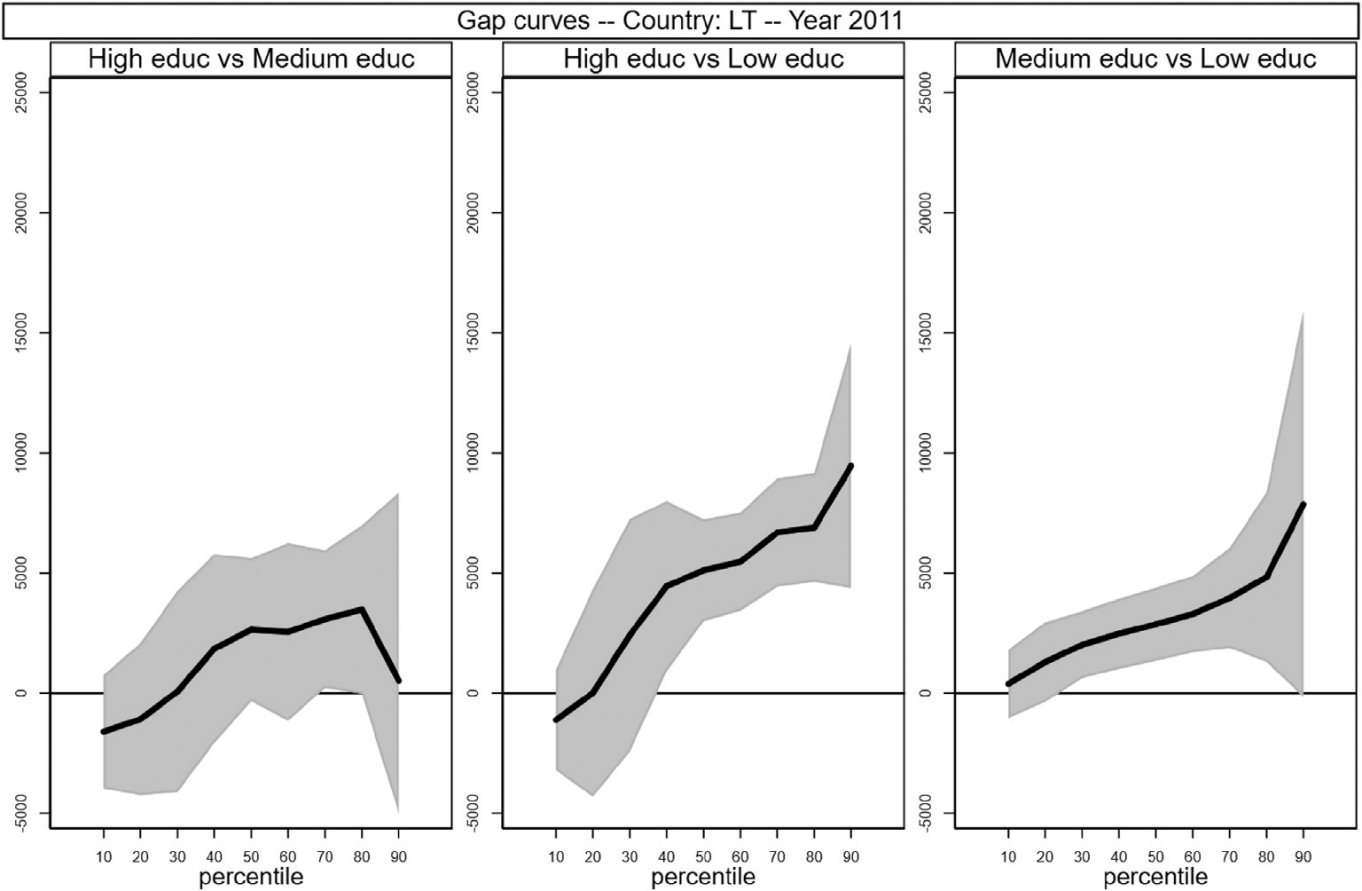
Gap curves for Lithuania.

**Fig. 10:**
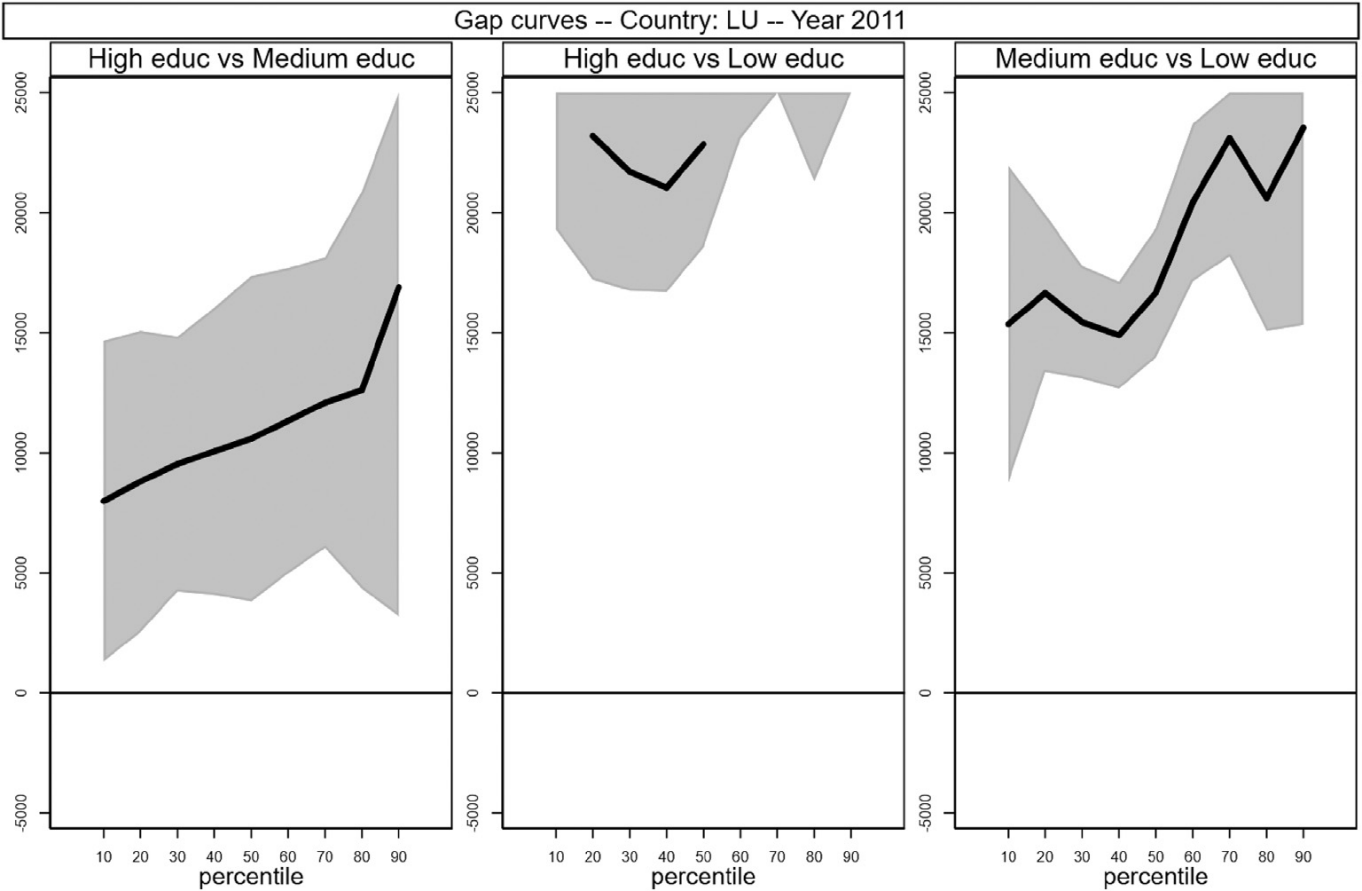
Gap curves for Luxembourg.

**Fig. 11:**
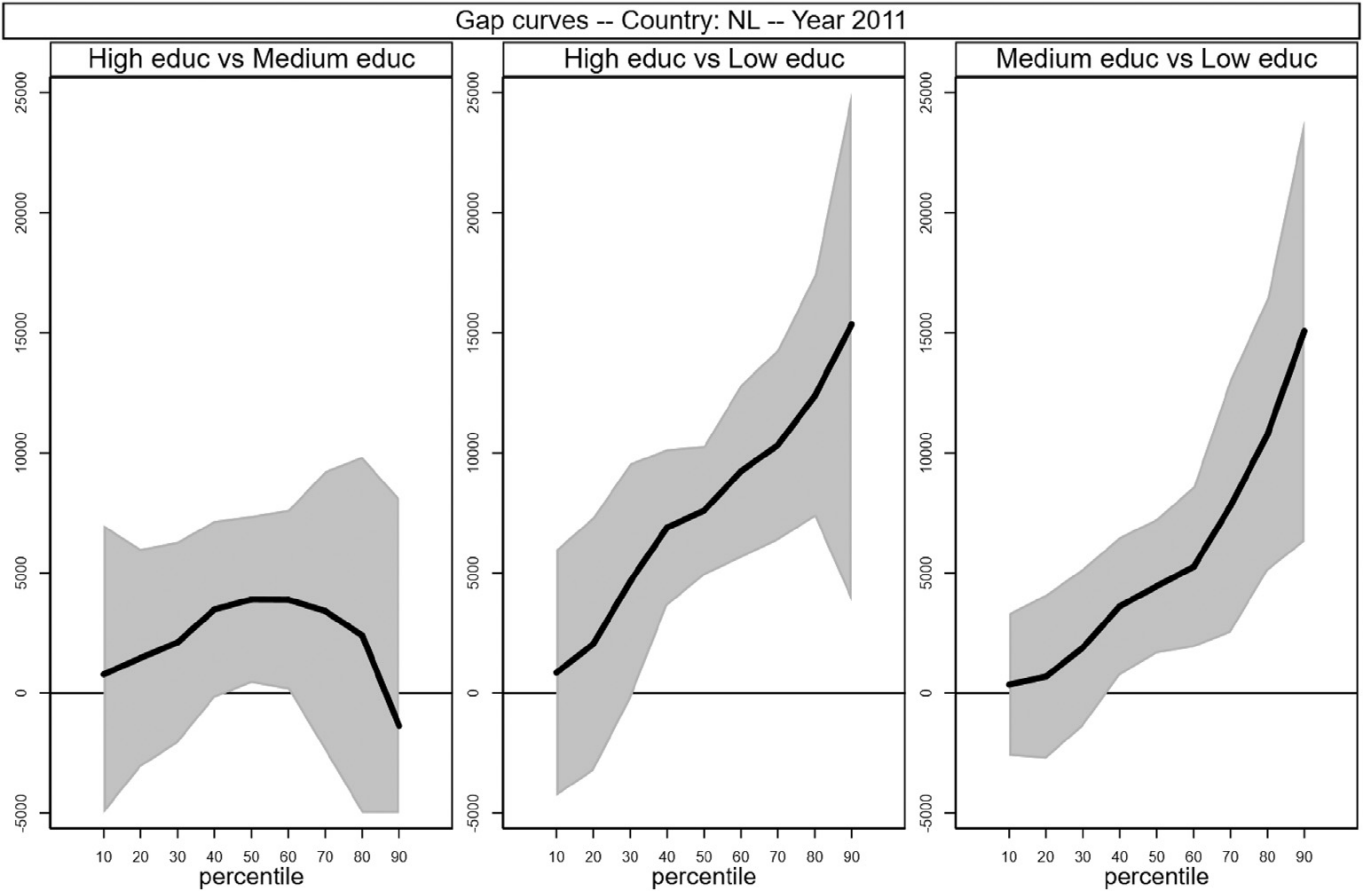
Gap curves for the Netherland.

**Fig. 12:**
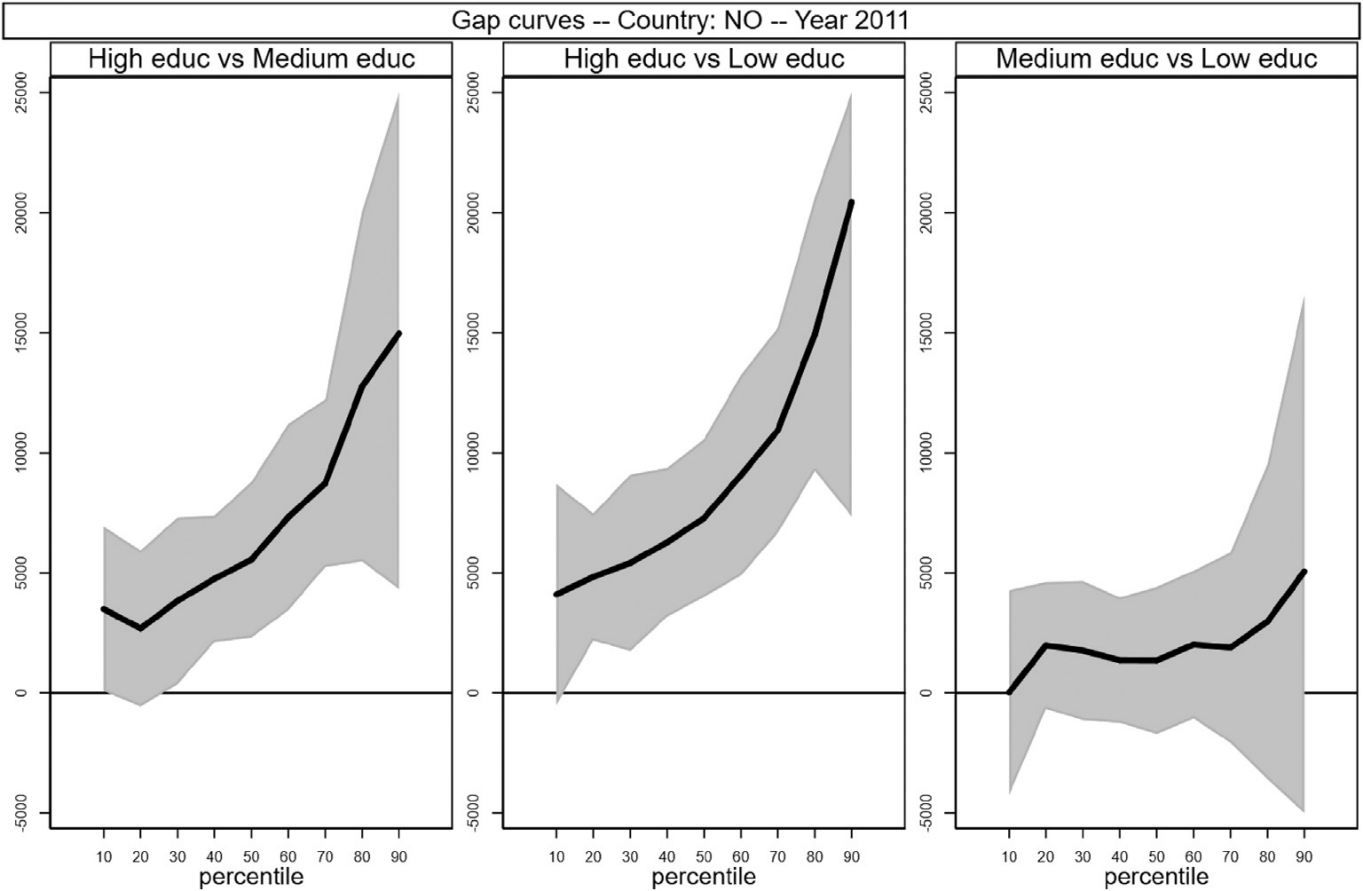
Gap curves for Norway.

**Fig. 13:**
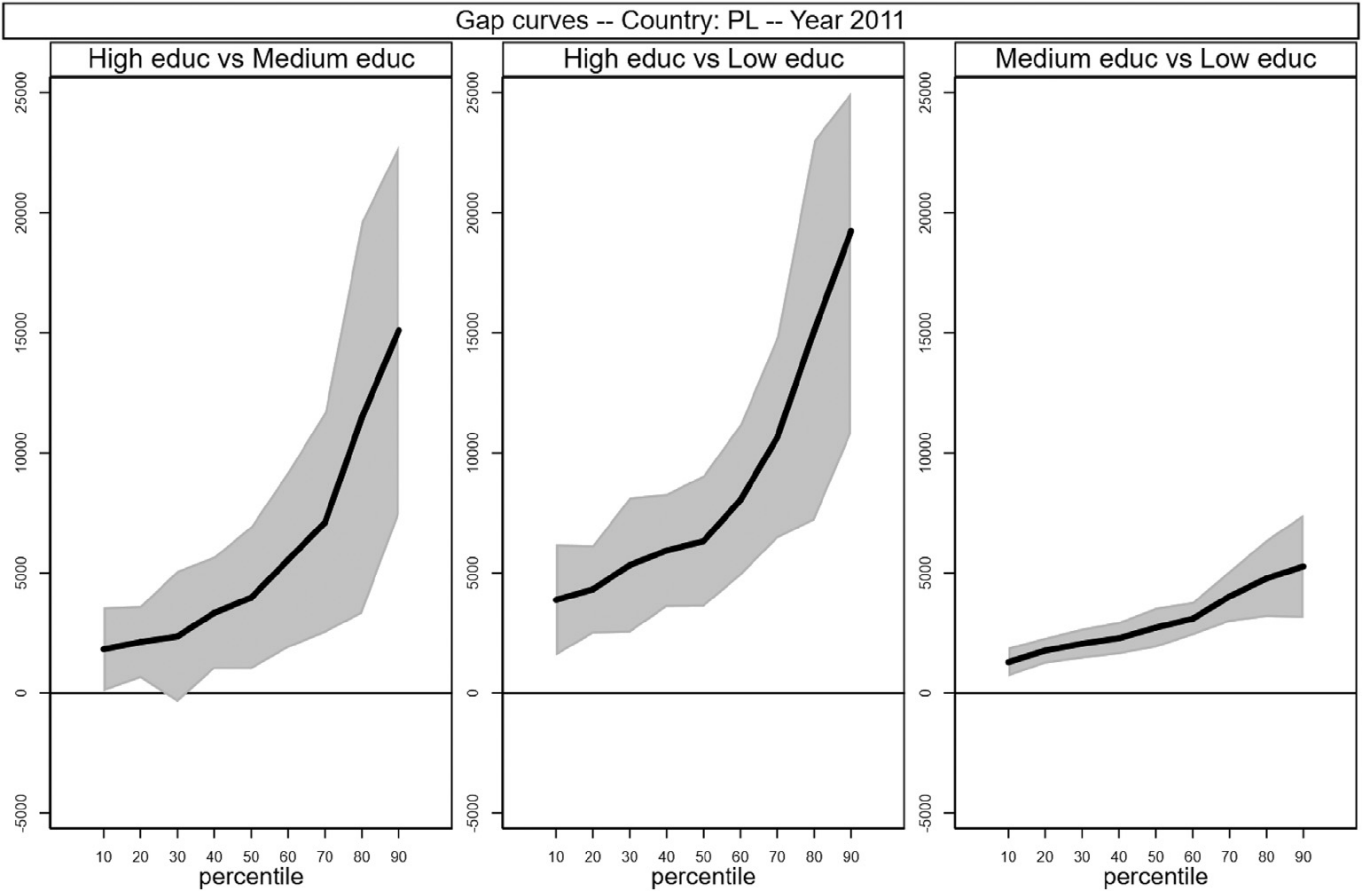
Gap curves for Poland.

**Fig. 14:**
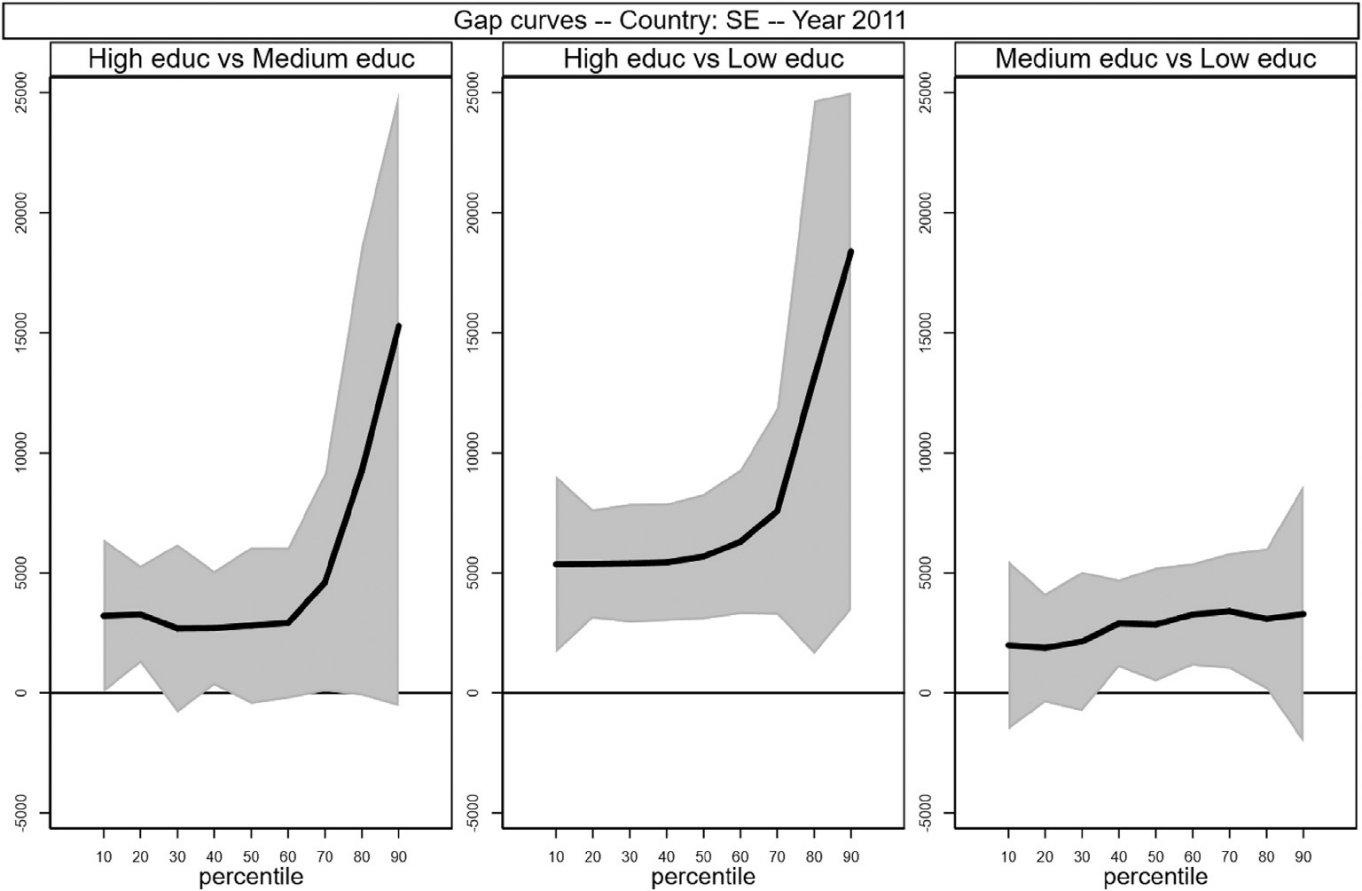
Gap curves for Sweden.

**Fig. 15:**
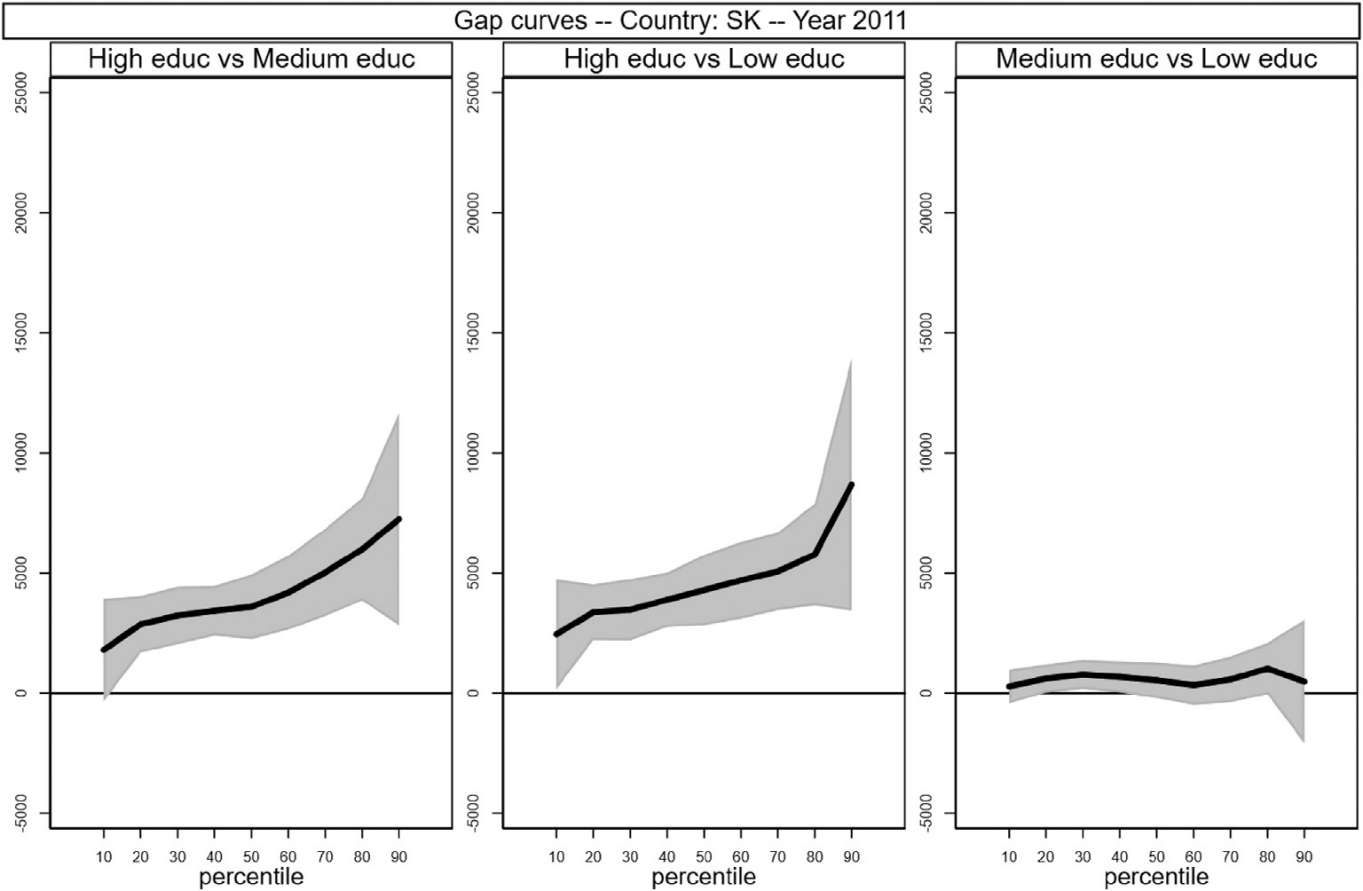
Gap curves for Slovakia.

**Fig. 16:**
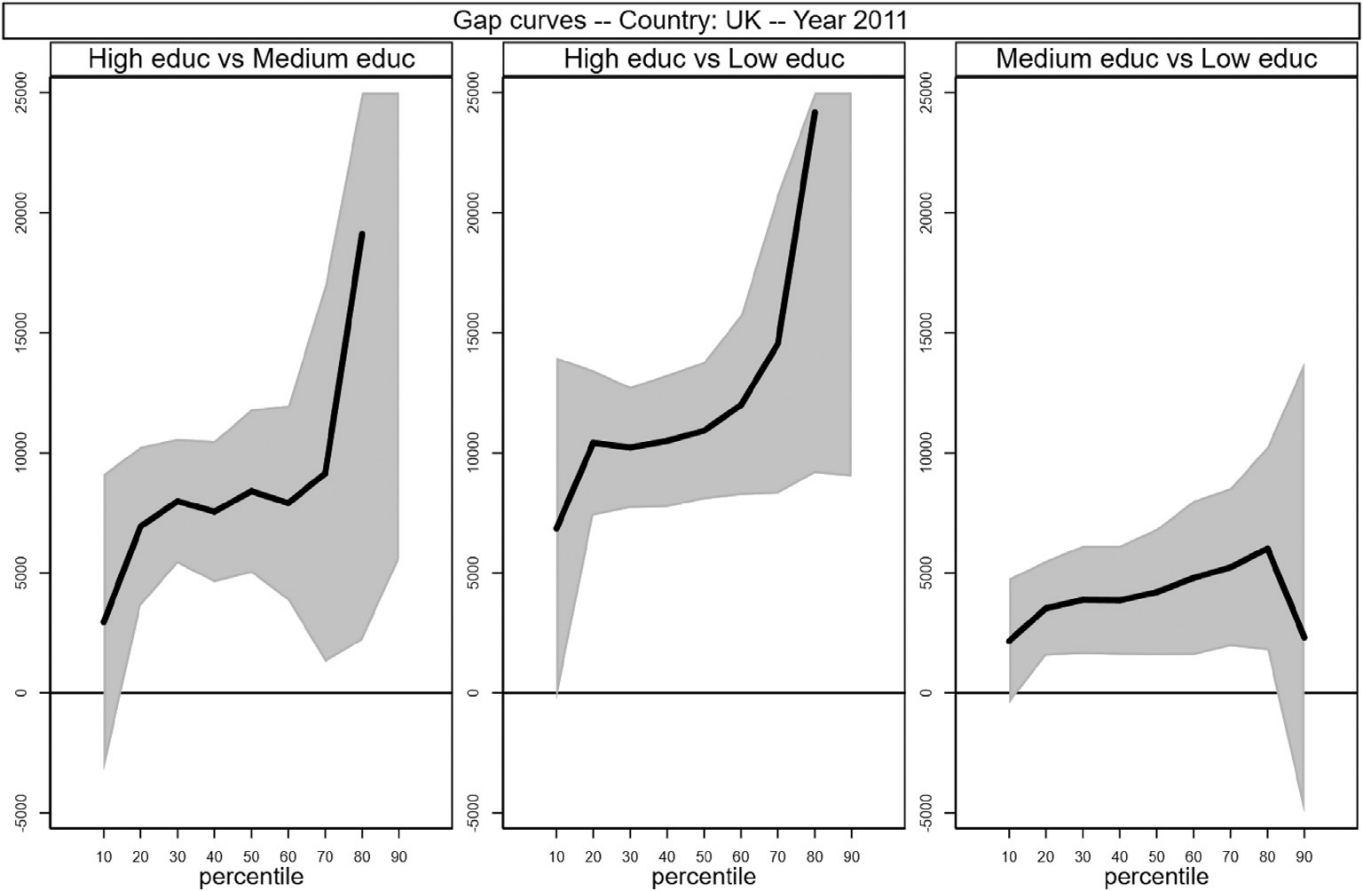
Gap curves for the UK.

*Circumstances*. The 2011 EU-SILC module contain retrospective information about parents' educational attainment, occupational status, labour market activity status, family composition as well as presence of financial difficulties during respondents' teenage years. We focus on the educational attainment of the father as the relevant circumstance. To construct circumstances, individuals are first partitioned in three types (or groups) according to their father's education. The *high education* type consists of individuals who lived in a household where the father attained the first (e.g. bachelor, master or equivalent) or second (e.g. PhD or equivalent) stage of tertiary education; the *medium education* type consists of individuals who lived in a household where the father attained upper secondary education and post-secondary, non-tertiary education. Finally, the *low education* type consists of individuals who lived in a household where the father at most completed lower secondary education. Table 2 summarizes the circumstances assignment rule adopted.

**Table 2:** Defining circumstances.

Type	Variable in EU-SILC: *pt110*: highest ISCED level of education attained by the father
Low education	- father could neither read nor write in any language - *low level* (pre-primary, primary education or lower secondary education)
Medium education	- *medium level* (upper secondary education and post-secondary non tertiary education)
High education	- *high level* (first stage of tertiary education and second stage of tertiary education)

*Earnings*. Earnings correspond to annual gross employee cash or near cash income data. This income measures is defined as the monetary component of the compensation in cash payable by an employer to an employee, and it includes the value of any social contributions and income taxes payable by an employee or by the employer on behalf of the employee to social insurance schemes or tax authorities. This variable reflects the relation between the labour income and individual circumstances before state intervention. The observed earnings were converted in purchasing power standard (PPS) using the conversion rates provided on the CIRCABC user group. For references, see: https://circabc.europa.eu/w/browse/3c60eeec-aca4-4db7-a035-0a6d892e6069.

Data reproduced in Table 1 and Fig. 1 in [1] are estimates of econometric models that are run on data from the selected running sample. Econometric models allow to filter out residual uncertainty and produce estimates of opportunity profiles at country level, and compare these estimates across countries.

## 2 Experimental design, materials, and methods

Andreoli and Fusco [1] use earnings as a metric for opportunities (see also Andreoli and Fusco [2]). Two caveats apply. First, this variable is defined at the level of the individual, implying that labour supply decisions are assumed to be made at individual level, thus neglecting household bargaining issues. Second, wages represent yearly evaluations of performances, since we focus on individuals who spent more than six months in the income reference period as full-time workers.

Opportunity profiles are estimated via Recentered Influence Function methods (Firpo, Fortin and Lemieux [5]) to recover effects of circumstances on earnings quantiles, while controlling for age and marital status. We estimate standard errors and variance-covariance matrices via bootstrapped resampling procedures on baseline data, where stratification by country, year and region of residence (“psu” variable in *example_econletters.dta*) is accounted for (see Goedemé [6]).

The estimation algorithm proceeds as follows:

1) draw a bootstrapped sample from the using sample;
2) estimate RIF regression parameters, income levels and pdf at given preselected deciles for each bootstrapped sample;
3) calculate gap curves for each country, differences in gap curves across countries for each pair of types and aggregated inequality of opportunity indices for each country and their variations across countries;
4) reiterate the bootstrap procedure 250 times;
5) compute averages and standard error of gap curves, differences in gap curves, IOp indices and store estimates;
6) produce graphs of gap curves and of their 95% confidence interval based on bootstrapped standard errors at specific earnings deciles identified in point 2);
7) estimate variance-covariance matrices from bootstrapped data and use them to test relevant hypothesis, then test these hypothesis and count cases (passed on pairwise comparisons of types) for which an hypothesis is accepted or rejected.
8) Report estimates in the form of tables.

The estimation procedure generates additional data, essentially estimates from the baseline specification of the econometric model, that are then elaborated to produce tables of results. Additional data are stored in the folder “∖output” of the data folder available in the repository. Notably, this folder contains the following datasets, all created from the resampling procedure:

- *bs_frale.dta*: reports estimates of regression coefficients estimates for RIF regressions, by country (country), income decile (percentile) and bootstrapped replica (rep).
- *bs2_frale.dta*: reports estimates of income deciles (pdf_pcty_X) and the corresponding type-specific pdf level (pdf_pcty_X) for each circumstance type X = 1,2,3 by country (country), income decile (percentile) and bootstrapped replica (rep).
- *meanGap0.dta*, reports average estimates of gap curves based on the whole running sample.
- *meanGap.dta*, reports average estimates of gap curves based on bootstrapped samples.
- *Chi2_data.dta*, collects data about gap curves estimates by deciles and country.
- *eop.dta*, reports values of test statistics for $H_{0}^{EOp}$ by country, see Andreoli and Fusco [1].
- *gapcountry.dta*, reshaped database, reports gap curves estimates by country (columns).
- *dataiop.dta*, reports the differences in gap curves of type X versus type Y across row country and column country Z, giving G_X_Y_cZ by country (country), income decile (percentile) and bootstrapped replica (rep).
- *iop.dta*, for each pair of countries (country country2), produce t-tests for differences in average gaps across types X and Y (test_G_X_Y_c) alongside the number of cases where equality in average gaps is accepted or rejected. Moreover, the file reports test statistics for equality in gap curves (Chi2G_X_Y), ascertain if $H_{0}^{IOp}$ is rejected or not for each comparison (accept_X) and then reports number of cases where $H_{0}^{IOp}$ is rejected or accepted.
- *GO_bs.dta*, reports estimates of GO index by country and of differences in GO index across countries. SE (bootstrapped) reported for levels and differences in GO index.

Table 1 in Andreoli and Fusco [1] is based on these estimates. Tests for $H_{0}^{EOp}$ and $H_{0}^{IOp}$ against unrestricted alternatives require to impose equality constraints on vectors of parameter estimates that are jointly normally distributed (by assumption). Tests putting failure of gap curves dominance at the null against strong dominance at the alternative (a test adopted in [1] to verify gap curve dominance in those cross-countries comparisons where $H_{0}^{IOp}$ is rejected) can be estimated from t-tests for differences in gap curves at specific quantiles (see Andreoli [7], [8] for a discussion and application of these tests).

Fig. 1 in Andreoli and Fusco [1] is obtained by stacking graphs of gap curves of selected countries. All gap curves (and their 95% confidence intervals) estimated from the running sample are reported below. The figures are obtained from data in *gapcountry.dta* are collected in the folder ∖output∖graphs in the repository.
