# Supplementary material for: Application of the EU-SILC 2011 data module “intergenerational transmission of disadvantage” to robust analysis of inequality of opportunity
Source: Data Brief. 2019 Jul 23;25:104301. doi: 10.1016/j.dib.2019.104301 (PMC6685694; doi:10.1016/j.dib.2019.104301)
Supplement: Supplementary file 1 [file mmc1.zip › Data_in_Brief/DiB_FAAF_01.pdf]

Data article

Title: ***Application of the EU-SILC 2011 module "intergenerational transmission of disadvantage" to robust analysis of inequality of opportunity.***

Authors: Francesco Andreoli and Alessio Fusco

Affiliations: University of Verona and LISER; Luxembourg Institute of Socio-Economic Research

Contact email: F. Andreoli (corresponding author) Department of Economics, University of Verona, Via Cantarane 24, 37129 Verona, IT and LISER, 11 Porte des Sciences, L-4366, Esch-sur-Alzette, LU. Email: francesco.andreoli@liser.lu

Abstract

This data article describes the original data, the sample selection process and the variables used in Andreoli and Fusco (2019) to estimate gap curves for a sample of European countries. Raw data are from 2011 roaster of EU-SILC, cross-sectional sample of module "intergenerational transmission of disadvantage". This article reports descriptive statistics of the using sample. It also discusses the algorithm adopted to estimate the main effects and details the content of additional Stata files stored on the online repository. These additional files contain raw estimates from bootstrapped samples, which form the basis for estimating gap curves and their variance-covariance matrices. The data article also reports representations of gap curves for all 16 selected countries.

Specifications Table

Subject area	*Economics*
More specific subject area	*Public economics, welfare economics*
Type of data	*Tables and graphs*
How data was acquired	*Access to EU-SILC 2011 wave granted within the NETSILC2 collaborative network. Data available from Eurostat upon request, see Microdata Access Workflow Tool.*
Data format	*Raw data (not uploaded on the server), anonymized sample used in the analysis (uploaded), bootstrapped estimators (uploaded) are all in Stata format.*
Experimental factors	*NA*
Experimental features	*NA*
Data source location	*NA*
Data accessibility	*Raw data are not available on the public repository. They can be accessed through Eurostat upon request, see Microdata Access Workflow Tool. An anonymized using sample is made available.*

Value of the data

- EU-SILC data represent the baseline survey introduced by the European Commission and managed by Eurostat to monitor and compare standard of living across European countries.
- Data are highly harmonized across countries, and collected by central statistical institutes. This guarantees a high degree of comparability of countries in terms of the main variables we consider to define earnings opportunities and parental circumstances.
- Data are available free of charge in selected institutions in Europe (such as LISER). Users can apply for a visiting scheme which grants resources (material and knowledge-based) to the users of these data.

Data

The raw data are taken from the *European Union - Statistics on Income and Living Conditions* (EU-SILC) 2011 module on intergenerational transmission of disadvantage, where measures of parental background for a sufficiently large number of respondents are available. This module provides repeated cross-sectional information on the socioeconomic background of origin of the individuals interviewed in EU-SILC, along with standard relevant measures of labour market outcomes. In particular, the 2011 module contains retrospective information about the parental background experienced by the respondents when aged between 12 and 16 (see Atkinson *et al*, 1983 for pros and cons of retrospective data). This unique base provides (to a large extent) comparable data allowing similar definitions for variables measuring outcome and circumstances across countries and time. An assessment of the 2011 EU-SILC module can be found here: http://ec.europa.eu/eurostat/web/income-and-living-conditions/data/ad-hoc-modules. Due to use restrictions rules, the raw data cannot be uploaded on the repository. We add workable Stata files reporting the information on data cleaning and sample selection routine.

On the repository, we report an anonymized version of the working sample we use to run our estimates. This sample is taken from EU-SILC 2011 module data (cross-section). We focus on a subset of 16 countries: Austria (AT), Belgium (BE), Germany (DE), Estonia (EE), Finland (FI), Hungary (HU), Ireland (IE), Iceland (IS), Lithuania (LT), Luxembourg (LU), the Netherlands (NL), Norway (NO), Poland (PL), Sweden (SE), Slovakia (SK) and the United Kingdom (UK). Our interest is on individual measures of income opportunities. To estimate opportunity profiles, we restrict attention to males aged between 30 and 50 who worked full time as an employee for at least 7 months in the income reference period. In addition, individuals who declared that they were living in another private household, foster home, collective household or institution were excluded. Following Raitano and Vona (2015), we use the intergenerational module weights.

The 2011 EU-SILC module contain retrospective information about parents' educational attainment, occupational status, labour market activity status, family composition as well as presence of financial difficulties during respondents' teenage years. We focus on the educational attainment of the father as the relevant circumstance. This choice, which is in line with previous literature, is driven by comparability motives and by sample size requirements at the moment of estimating the unfair disadvantage distribution. To construct circumstances, individuals are first partitioned in three types (or groups) according to their father's education. The *high education* type consists of individuals who lived in a household where the father attained the first (e.g. bachelor, master or equivalent) or second (e.g. PhD or equivalent) stage of tertiary education; the *medium education* type consists of individuals who lived in a household where the father attained upper secondary education and post-secondary, non-tertiary education. Finally, the *low education* type consists of individuals who lived in a household where the father at most completed lower secondary education. Table 1 summarizes the rule adopted to generate the circumstance variable.

Table 1: Defining circumstances

**Type**	**Variable in EU-SILC**: ***pt110***: highest ISCED level of education attained by the father
**Low education**	- father could neither read nor write in any language<br>- *low level* (pre-primary, primary education or lower secondary education)
**Medium education**	- *medium level* (upper secondary education and post-secondary non tertiary education)
**High education**	- *high level* (first stage of tertiary education and second stage of tertiary education)

Our outcome variable of interest is the annual gross employee cash or near cash income. It is defined as the monetary component of the compensation in cash payable by an employer to an employee, and it includes the value of any social contributions and income taxes payable by an employee or by the employer on behalf of the employee to social insurance schemes or tax authorities. This variable reflects the relation between the labour income and individual circumstances before state intervention. Two caveats apply to this particular metric of opportunities. First, this variable is defined at the level of the individual, implying that labour supply decisions are assumed to be made at individual level, thus neglecting household bargaining issues. Second, wages represent yearly evaluations of performances, since we focus on individuals who spent more than six months in the income reference period as full-time workers. The observed earnings were converted in purchasing power standard (PPS) using the conversion rates provided on the CIRCABC user group. For references, see: https://circabc.europa.eu/w/browse/3c60eeec-aca4-4db7-a035-0a6d892e6069.

Additionally, we consider information about marriage status (we use an indicator for married male respondents) and age of respondents.

Our selected running sample is made of 41533 male respondents. The distribution of parental education circumstances, average earnings by parental education, average age and proportion of married individuals are reported in Table 2. Data are collected in the *example_econletters.dta* file in Stata format (optimized for Stata 13).

Table 2: Summary statistics of running sample

**Country**	**N**	**Types**			**Earnings**				**Age**	**Married**
		High	Medium	Low	All	High	Medium	Low		
AT	2887	0.10	0.43	0.48	37,320	49,367	39,829	32,604	40.4	0.69
BE	2446	0.19	0.23	0.57	38,788	54,702	37,742	33,792	40.1	0.65
DE	5345	0.30	0.58	0.11	41,444	44,228	40,642	38,108	41.4	0.75
EE	1777	0.18	0.43	0.40	12,966	17,494	13,398	10,508	40.4	0.64
FI	1949	0.21	0.22	0.56	31,245	41,842	30,229	27,627	40.4	0.61
HU	3825	0.10	0.36	0.54	11,548	19,096	12,506	9,476	39.8	0.69
IE	1122	0.14	0.22	0.65	40,408	52,155	48,067	35,358	40.2	0.74
IS	835	0.14	0.50	0.35	35,873	40,840	37,189	31,950	40.1	0.59
LT	1716	0.11	0.29	0.60	9,546	13,485	10,424	8,426	41.4	0.87
LU	2883	0.13	0.31	0.56	48,562	67,307	57,617	39,039	39.7	0.69
NL	2310	0.21	0.27	0.52	44,900	52,415	48,198	40,212	40.1	0.64
NO	1622	0.28	0.43	0.29	40,774	47,395	39,119	36,872	40.2	0.57
PL	5805	0.06	0.49	0.45	13,641	19,894	14,599	11,726	39.9	0.86
SE	1349	0.16	0.24	0.60	30,673	39,868	32,158	27,583	39.7	0.48
SK	2977	0.10	0.60	0.31	10,809	15,002	10,699	9,702	40.3	0.80
UK	2685	0.17	0.25	0.58	43,383	57,191	46,342	38,034	40.4	0.66
**Total**	41533	0.16	0.40	0.44	29,447	41,888	29,187	25,230	40.3	0.71

Experimental Design, Materials and Methods

We use Recentered Influence Function methods (Firpo, Fortin and Lemieux, 2009) to recover effects of circumstances on earnings quantiles, while controlling for age and marital status. We estimate standard errors and variance-covariance matrices via bootstrapped resampling procedures on baseline data, where stratification by country, year and region of residence ("psu" variable in *example_econletters.dta*) is accounted for (see Goedemé, 2013).

The estimation algorithm proceed s as follows:

1) draw a bootstrapped sample from the using sample;
2) estimate RIF regression parameters, income levels and pdf at given preselected deciles for each bootstrapped sample;

3) calculate gap curves for each country, differences in gap curves across countries for each pair of types and aggregated inequality of opportunity indices for each country and their variations across countries;
4) reiterate the bootstrap procedure 250 times;
5) compute averages and standard error of gap curves, differences in gap curves, IOp indices and store results;
6) produce graphs of gap curves and of their 95% confidence interval based on bootstrapped standard errors at specific earnings deciles identified in point 2);
7) estimate variance-covariance matrices from bootstrapped data and use them to test relevant hypothesis, then test these hypothesis and count cases (passed on pairwise comparisons of types) for which an hypothesis is accepted or rejected.
8) Report results in the form of tables.

The whole procedure requires to generate output datasets which we store in the folder "\output" available in the repository. Notably, this folder contains the following datasets, all created from the resampling procedure:

- *bs_frale.dta*: reports estimates of regression coefficients estimates for RIF regressions, by country (country), income decile (percentile) and bootstrapped replica (rep).
- *bs2_frale.dta*: reports estimates of income deciles (pdf_pcty_X) and the corresponding type-specific pdf level (pdf_pcty_X) for each type X=1,2,3 by country (country), income decile (percentile) and bootstrapped replica (rep).
- *meanGap0.dta*, reports average estimates of gap curves based on the using sample.
- *meanGap.dta*, reports average estimates of gap curves based on bootstrapped samples.
- *Chi2_data.dta*, collects data about gap curves by country (deciles estimates).
- *eop.dta*, reports values of test statistics for H_0^{EOp}, see Andreoli and Fusco (2019).
- *gapcountry.dta*, reshaped database, reports gap curves estimates by country (columns).
- *dataiop.dta*, reports the differences in gap curves of type X versus type Y across row country and column country Z, giving G_X_Y_cZ by country (country), income decile (percentile) and bootstrapped replica (rep).
- *iop.dta*, for each pair of countries (country country2), produce t-tests for differences in average gaps across types X and Y (test_G_X_Y_c) alongside the number of cases where equality in average gaps is accepted or rejected. Moreover, the file reports test statistics for equality in gap curves (Chi2G_X_Y), ascertain if H_0^{IOp} is rejected or not for each comparison (accept_X) and then reports number of cases where H_0^{IOp} is rejected or accepted.
- *GO_bs.dta*, reports estimates of GO index by country and of differences in GO index across countries. SE (bootstrapped) reported for levels and differences in GO index.

Table 1 in Andreoli and Fusco (2019) is based on these estimates. Tests for H_0^{EOp} and H_0^{IOp} against unrestricted alternatives require to impose equality constraints on vectors of parameter estimates that are jointly normally distributed (by assumption). Tests putting failure of gap curves dominance at the null against strong dominance at the alternative (a test Andreoli and Fusco 2019 use to verify gap curve dominance in those cross-countries comparisons where H_0^{IOp} is rejected) can be

estimated from t-tests for differences in gap curves at specific quantiles (see Andreoli 2018 for a discussion about these tests).

Figure 1 in Andreoli and Fusco (2019) is obtained by stacking graphs of gap curves of selected countries. We report below all gap curves (and their 95% confidence intervals) estimated from the running sample. The figures are obtained from data in *gapcountry.dta* are collected in the folder \output\graphs in the repository.

Figure 1: gap curves for Austria

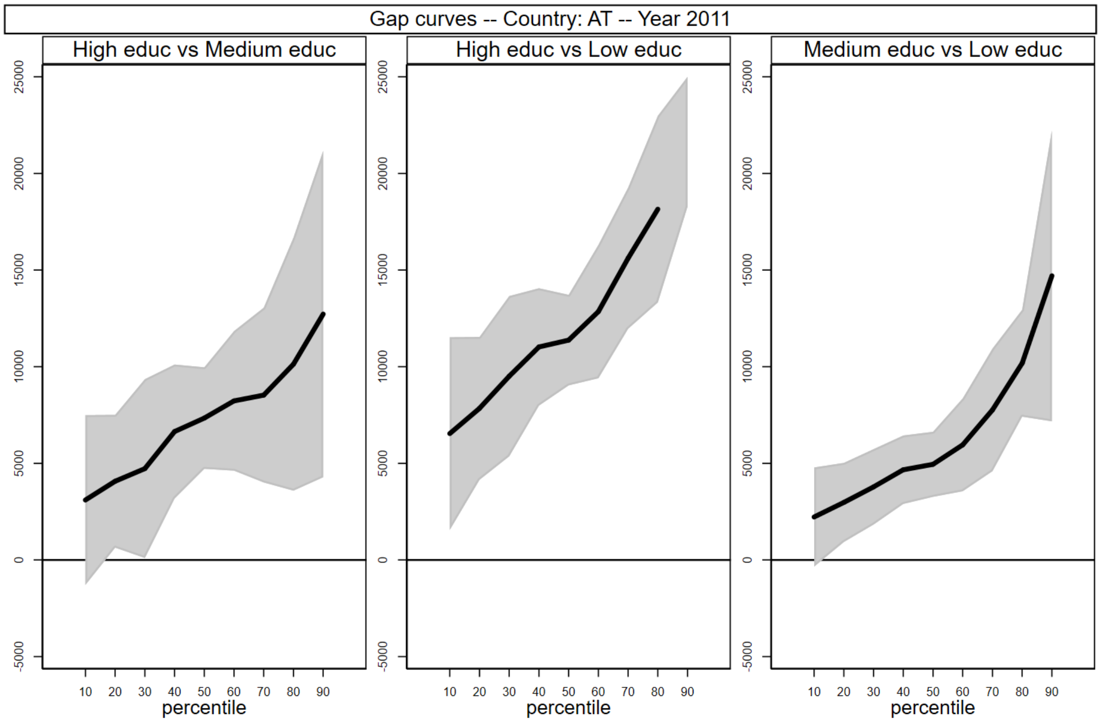

Figure 2: gap curves for Belgium

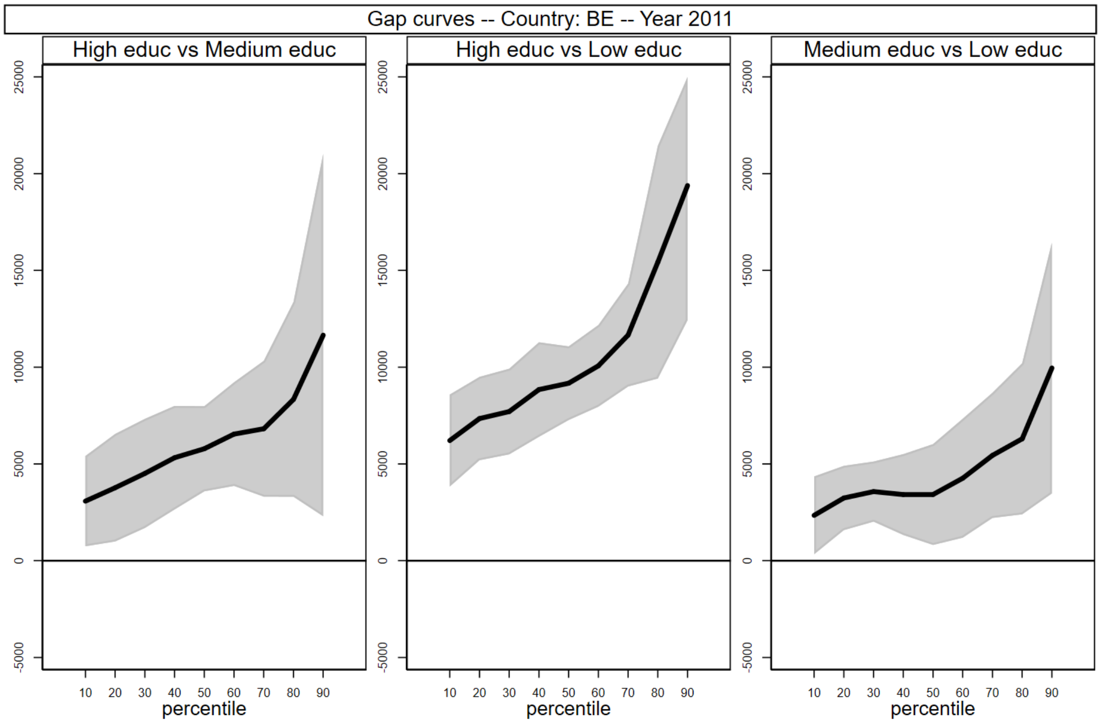

Figure 3: gap curves for Germany

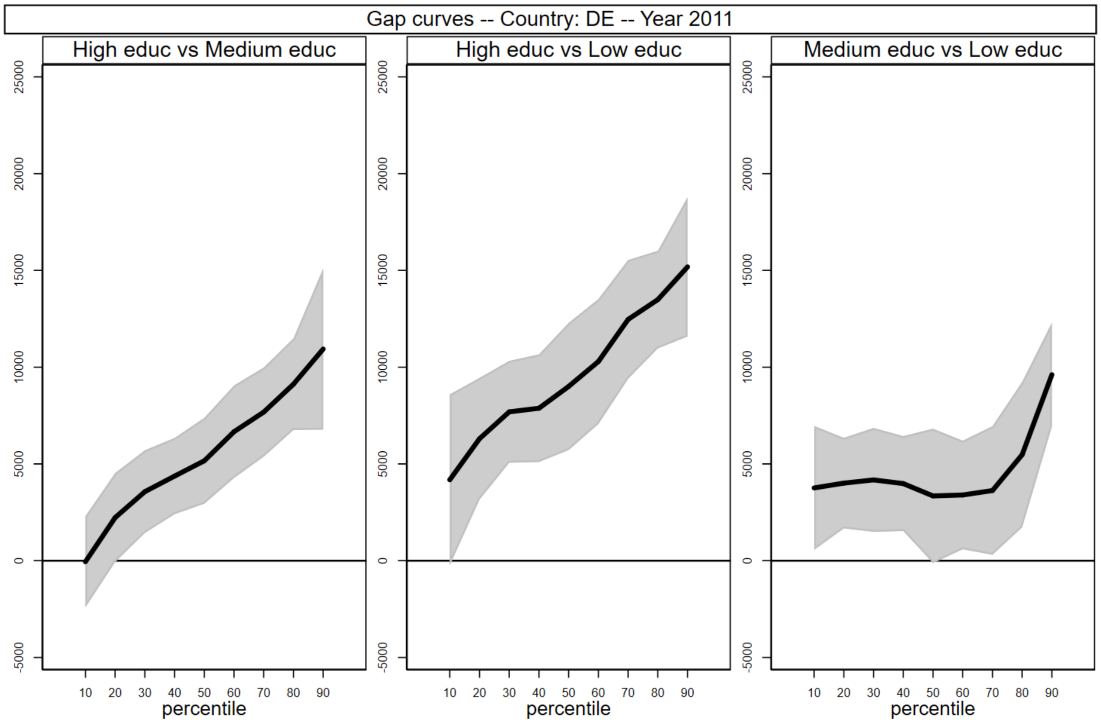

Figure 4: gap curves for Estonia

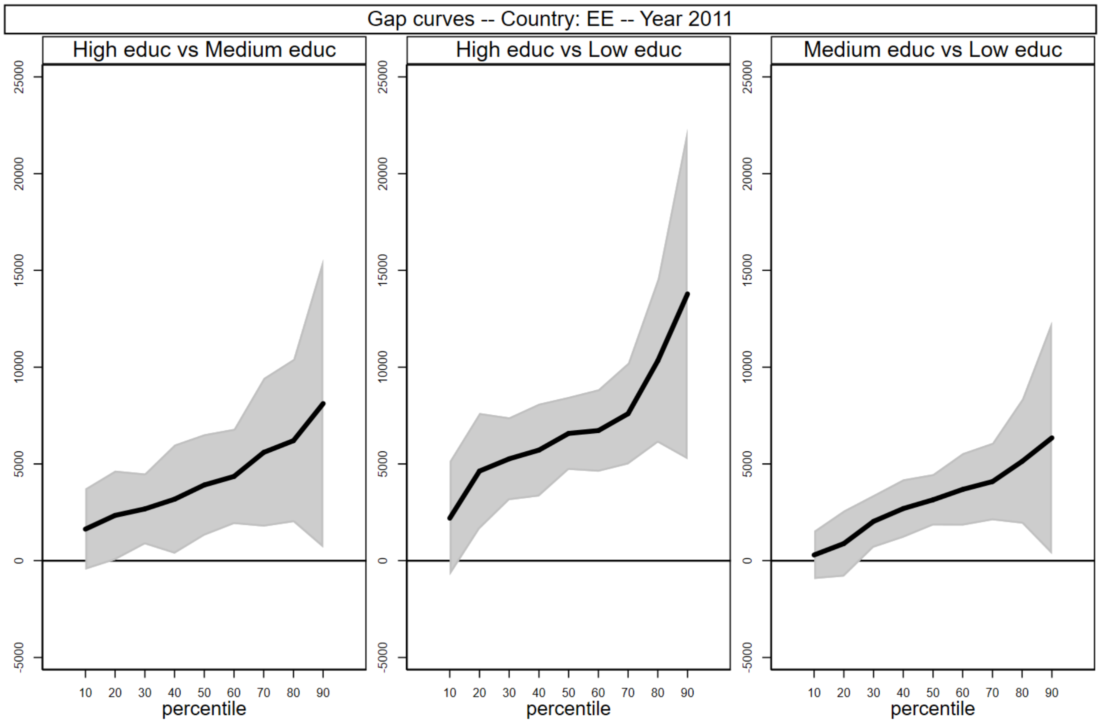

Figure 5: gap curves for Finland

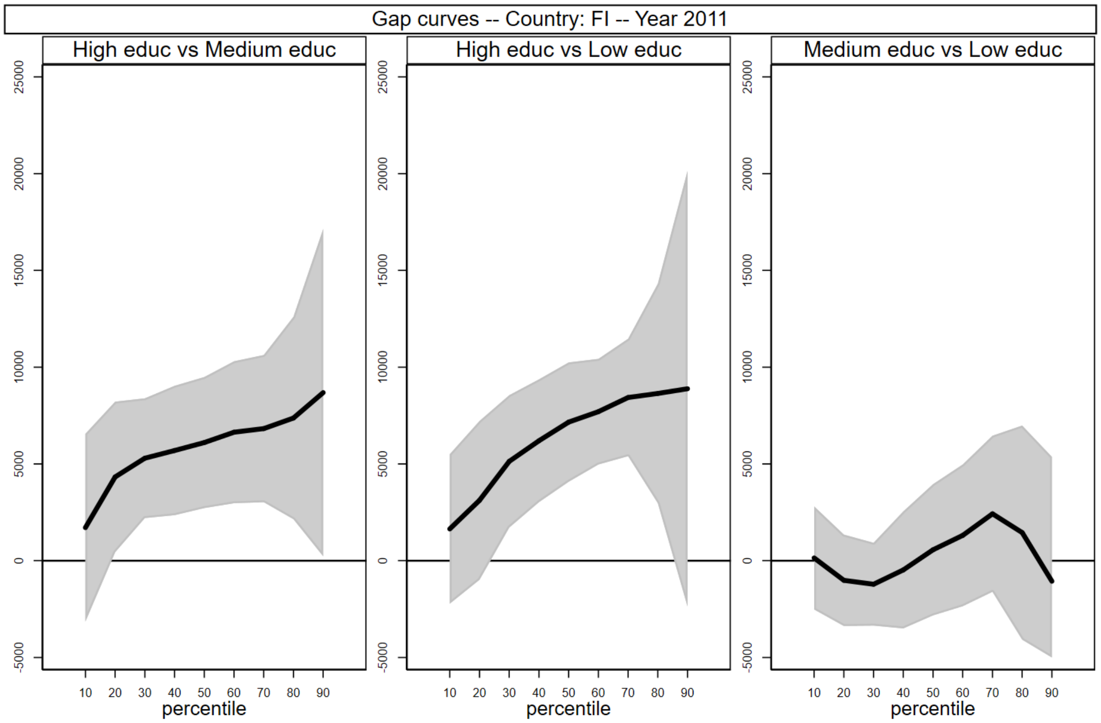

Figure 6: gap curves for Hungary

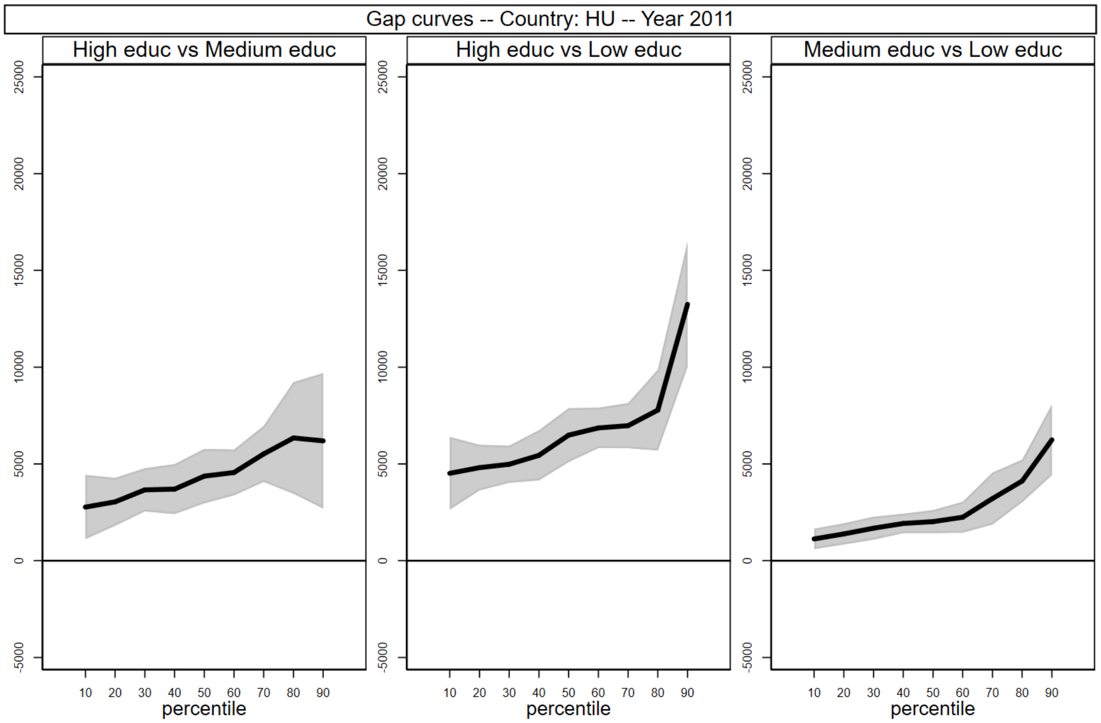

Figure 7: gap curves for Ireland

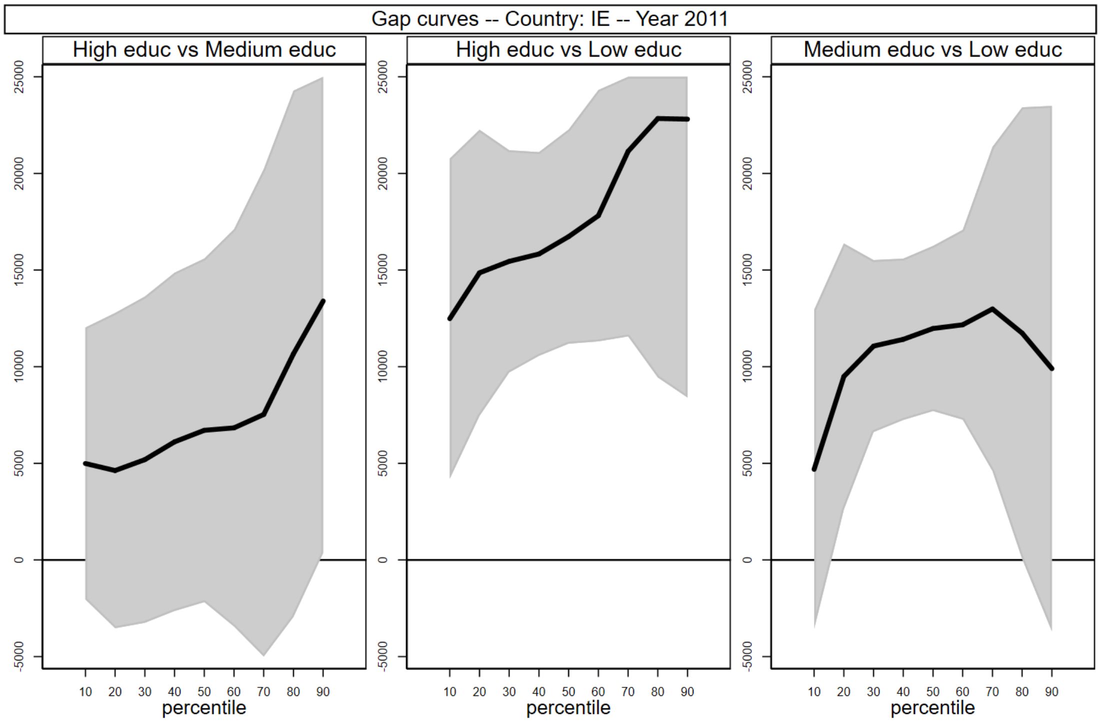

Figure 8: gap curves for Iceland

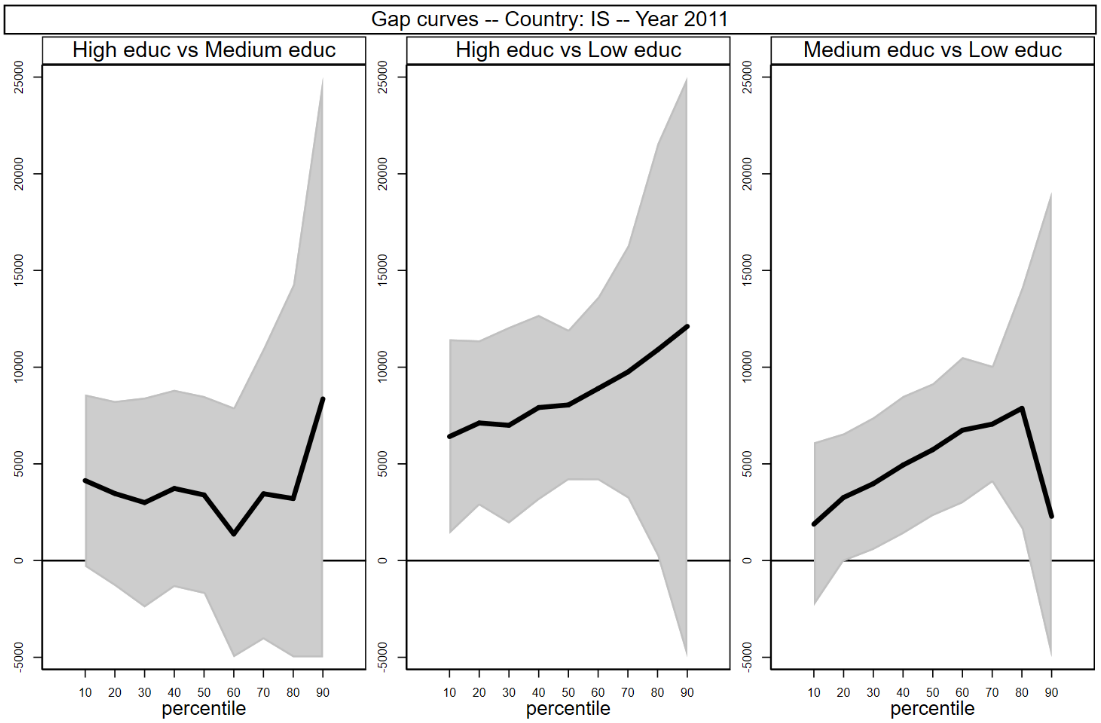

Figure 9: gap curves for Lithuania

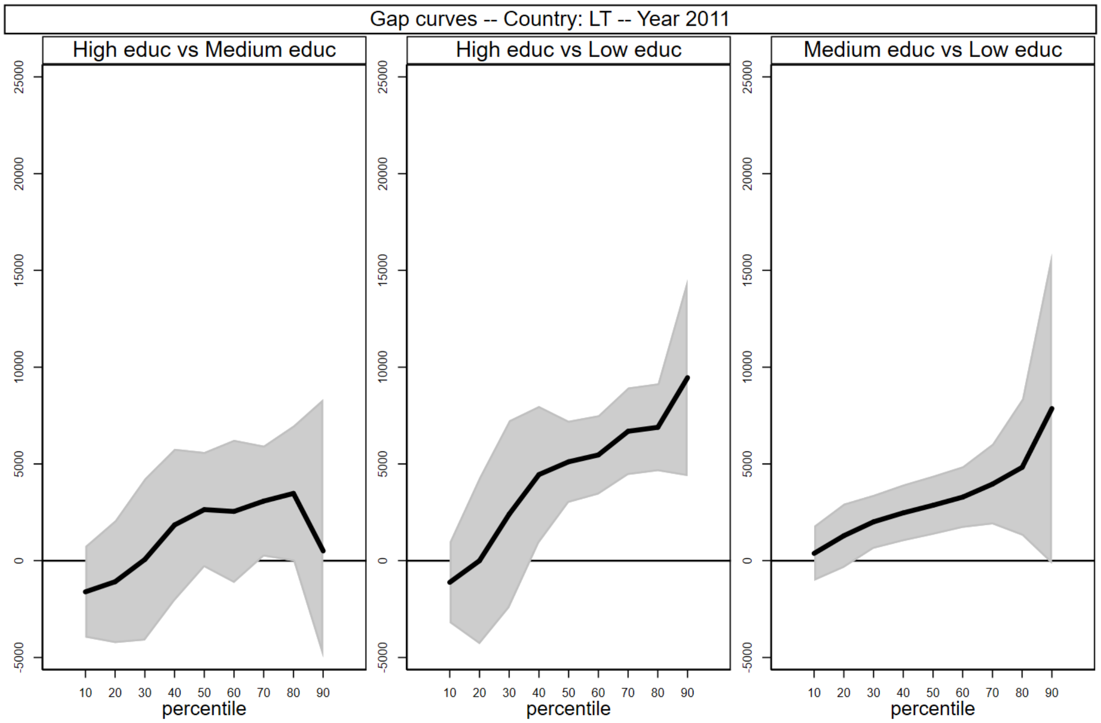

Figure 10: gap curves for Luxembourg

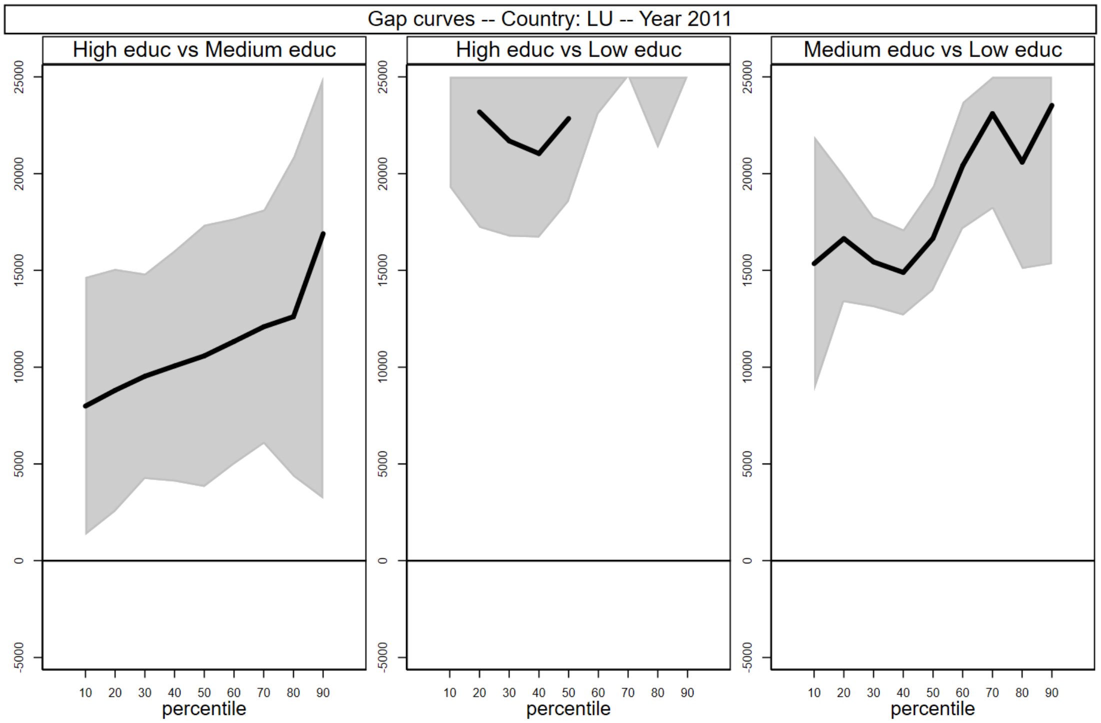

Figure 11: gap curves for the Netherland

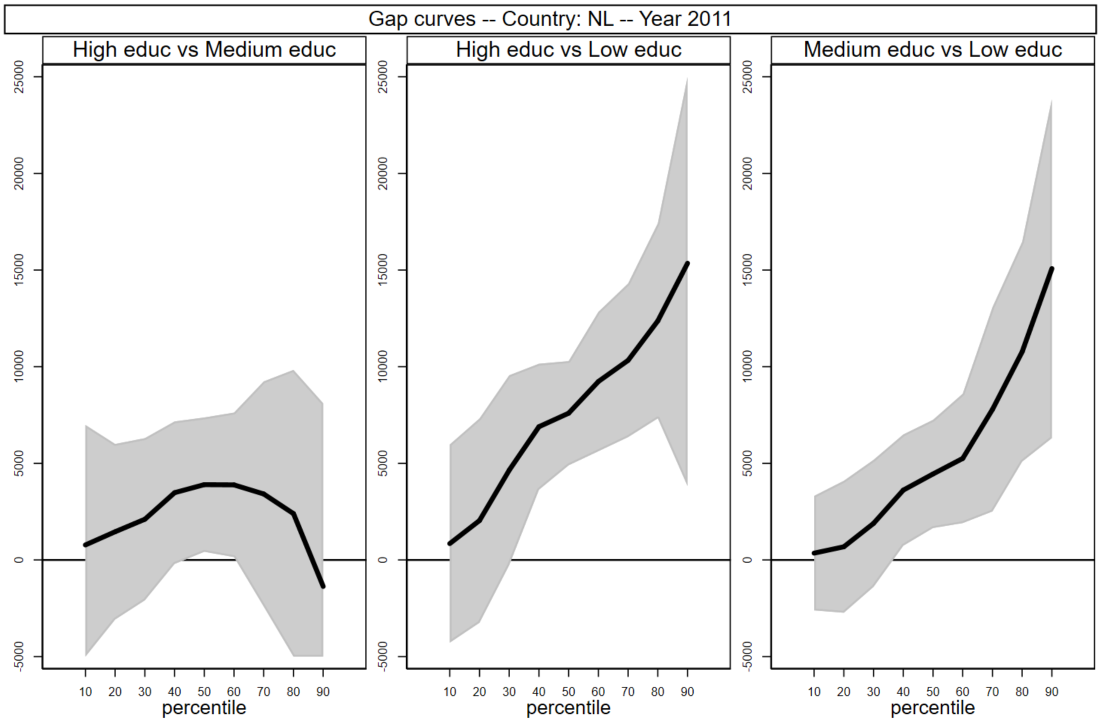

Figure 12: gap curves for Norway

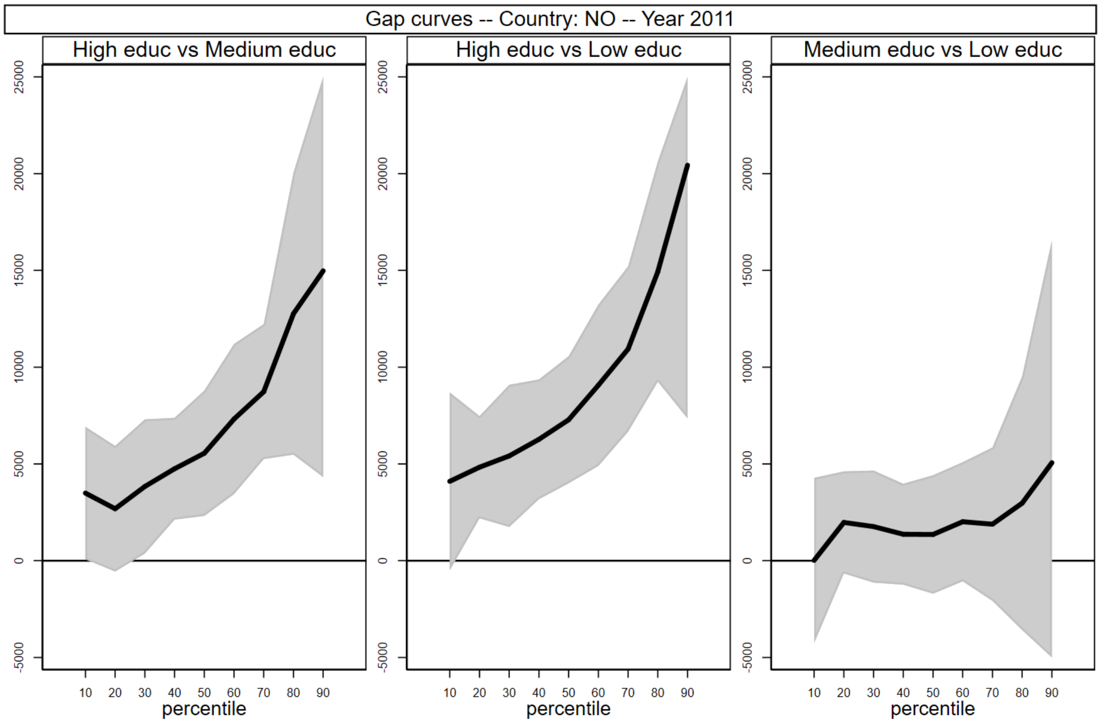

Figure 13: gap curves for Poland

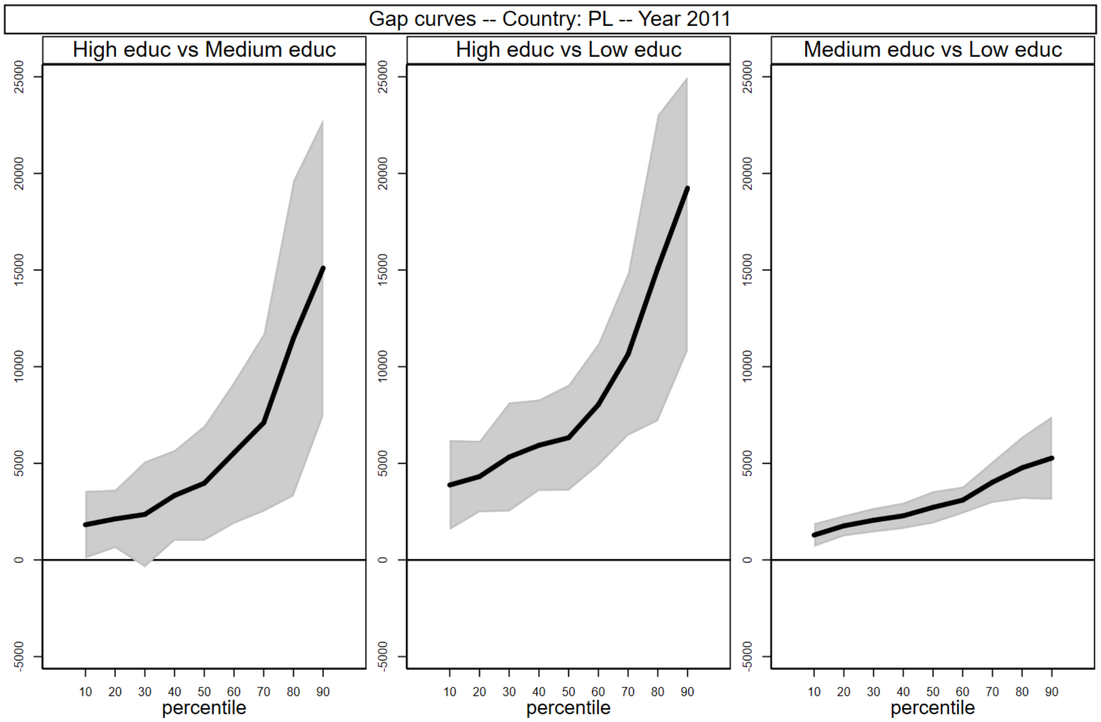

Figure 14: gap curves for Sweden

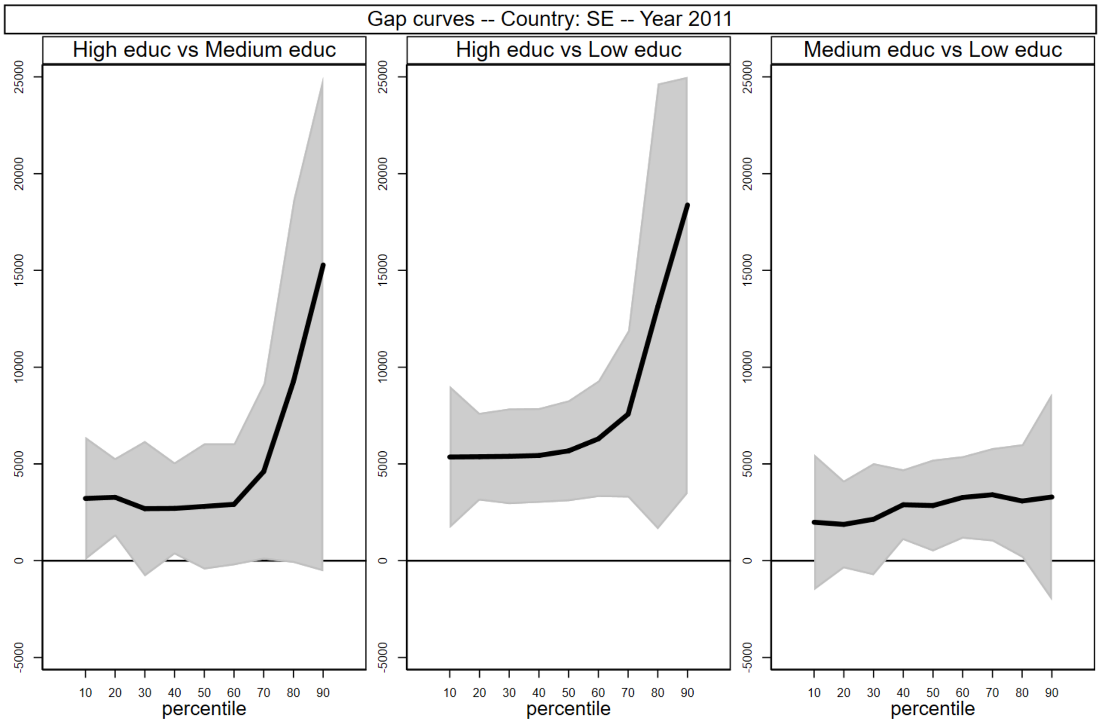

Figure 15: gap curves for Slovakia

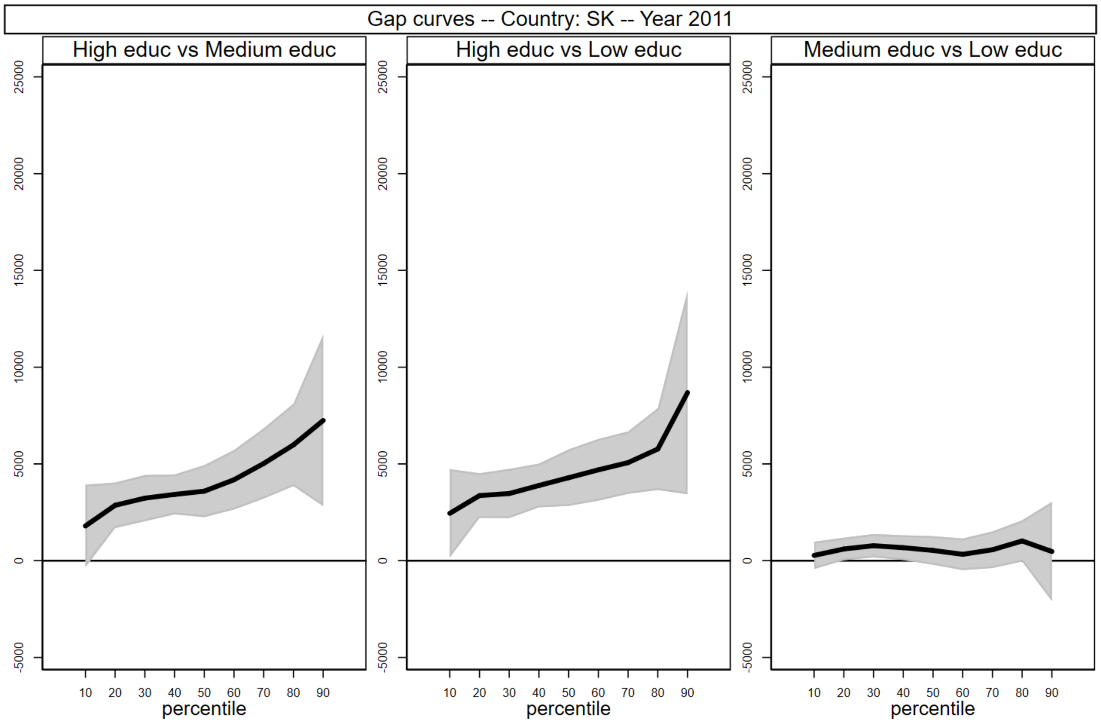

Figure 16: gap curves for the UK

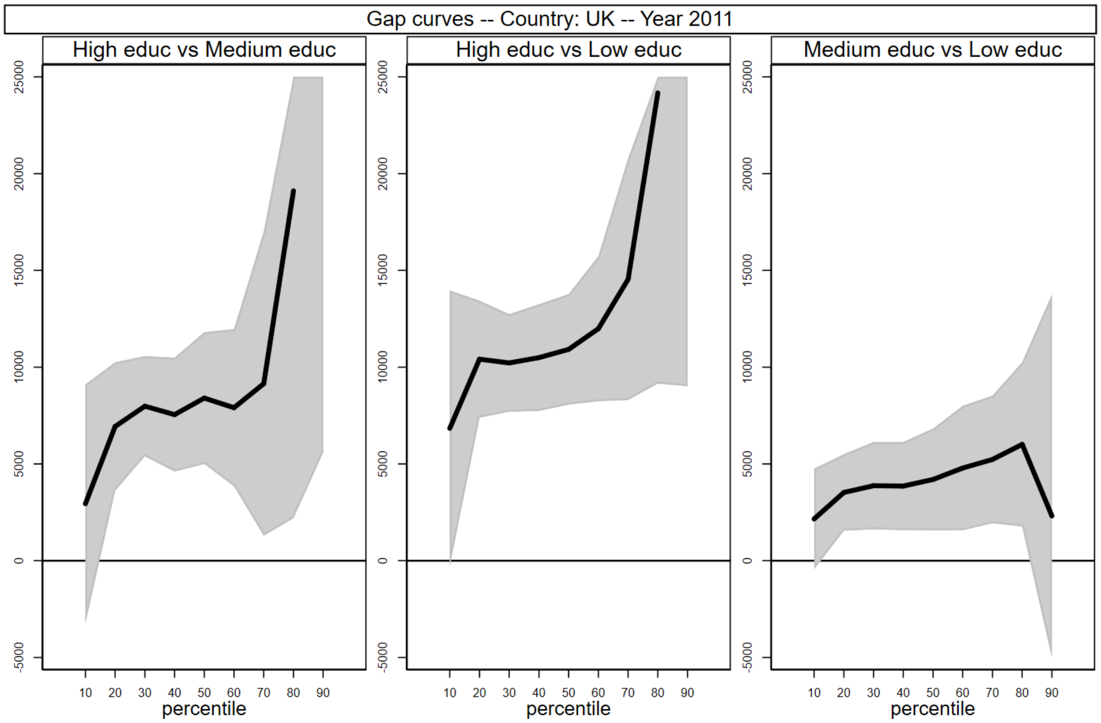

Acknowledgements

Financial support from Eurostat (Net-SILC2), the French Agence Nationale de la Recherche (Ordineq grant ANR-16-CE41-0005-01) and the Luxembourg Fonds National de la Recherche (IMCHILD grant INTER/NORFACE/16/11333934, PREFERME CORE grant C17/SC/11715898 and IMeRSe CORE grant C15/SC/10266168) is gratefully acknowledged. Fusco also thanks the World Bank Development Research Group for its hospitality.

References

Andreoli, F. (2018). Robust inference for inverse stochastic dominance, *Journal of Business & Economic Statistics* 36(1):146-159.

Andreoli, F., Fusco, A. (2017) The evolution of inequality of opportunity across Europe: EU-SILC evidence. Ch. 24 in A.B. Atkinson, A.C. Guio and E. Marlier (Eds.) *Monitoring Social Inclusion in Europe*, Eurostat, pp. 435-448.

Andreoli, F., Fusco, A. (2019) Robust cross-country analysis of inequality of opportunity, *mimeo.*

Atkinson, T., Maynard, A.K. and Trinder, C. in conjunction with Corlyon, J., Jenkins, S.P. and Sutherland, H. (1983), *Parents and children. Incomes in Two Generations, Studies in Deprivation and Disadvantage*, Heinemann educational Books, London.

Firpo, S., Fortin, N., Lemieux, T. (2009), "Unconditional quantile regressions", *Econometrica,* 77(3) :953-973.

Goedemé, T. (2013), "How much confidence can we have in EU-SILC? Complex sample designs and the standard error of the Europe 2020 poverty indicators", *Social Indicators Research* 110(1):89-110.

Raitano, M. and Vona, F. (2015), "Measuring the link between intergenerational occupational mobility and earnings: evidence from eight European countries", *Journal of Economic Inequality*. 13(1):83-102.
