# Supplementary figures and images for: Application of the EU-SILC 2011 data module “intergenerational transmission of disadvantage” to robust analysis of inequality of opportunity
Source: Data Brief. 2019 Jul 23;25:104301. doi: 10.1016/j.dib.2019.104301 (PMC6685694; doi:10.1016/j.dib.2019.104301)

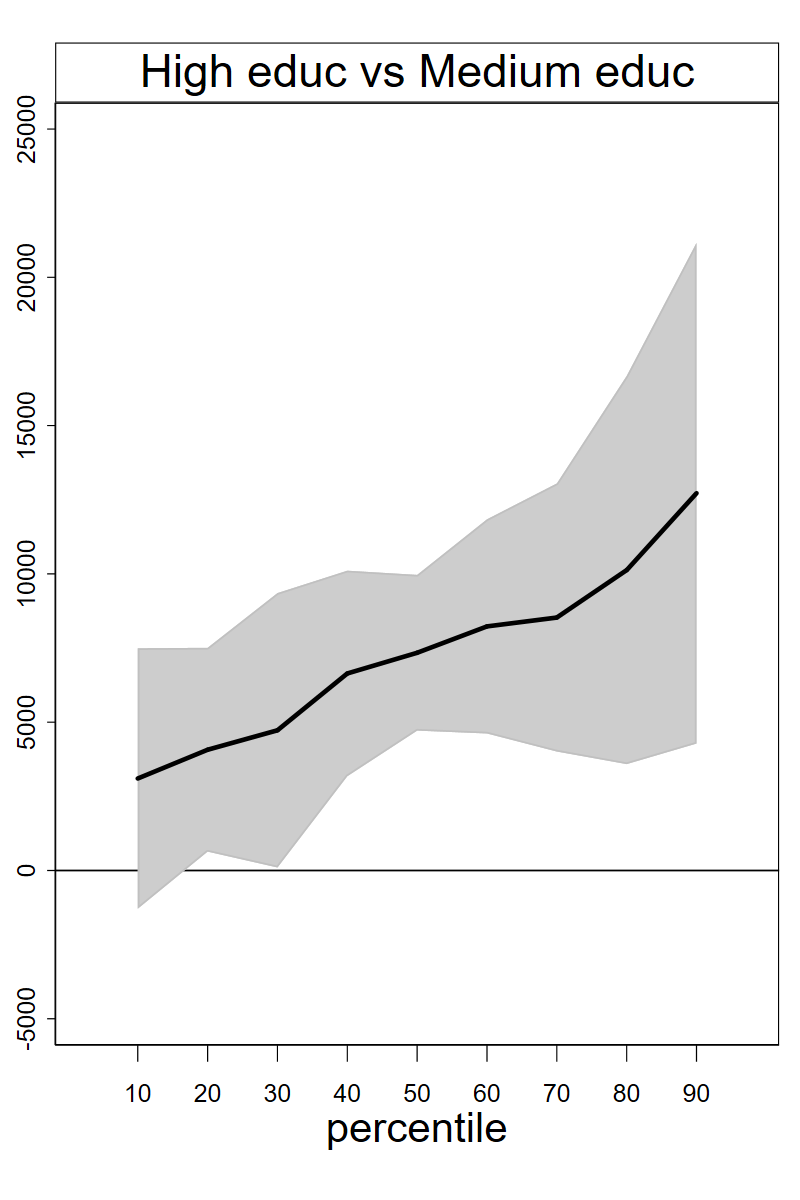

Supplement: Supplementary file 1 [file mmc1.zip › Data_in_Brief/output/graphs/G_1_2_2011_1.png]

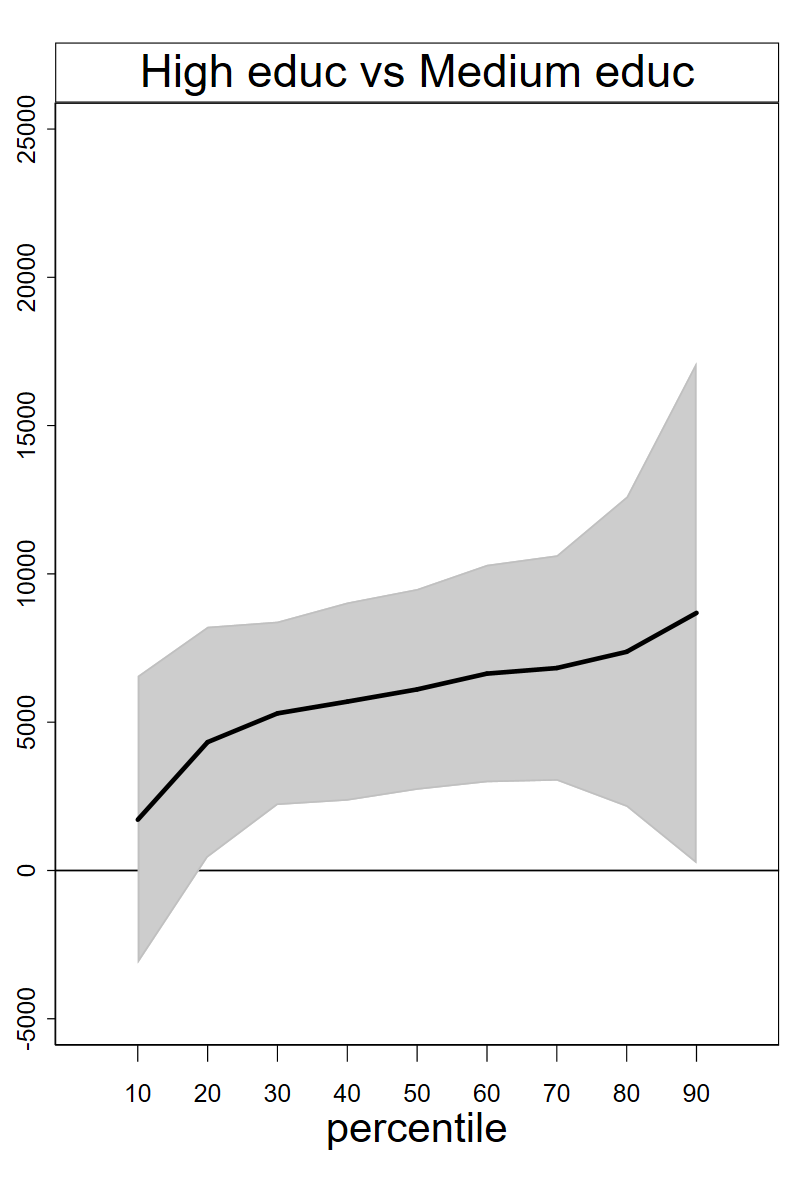

Supplement: Supplementary file 1 [file mmc1.zip › Data_in_Brief/output/graphs/G_1_2_2011_12.png]

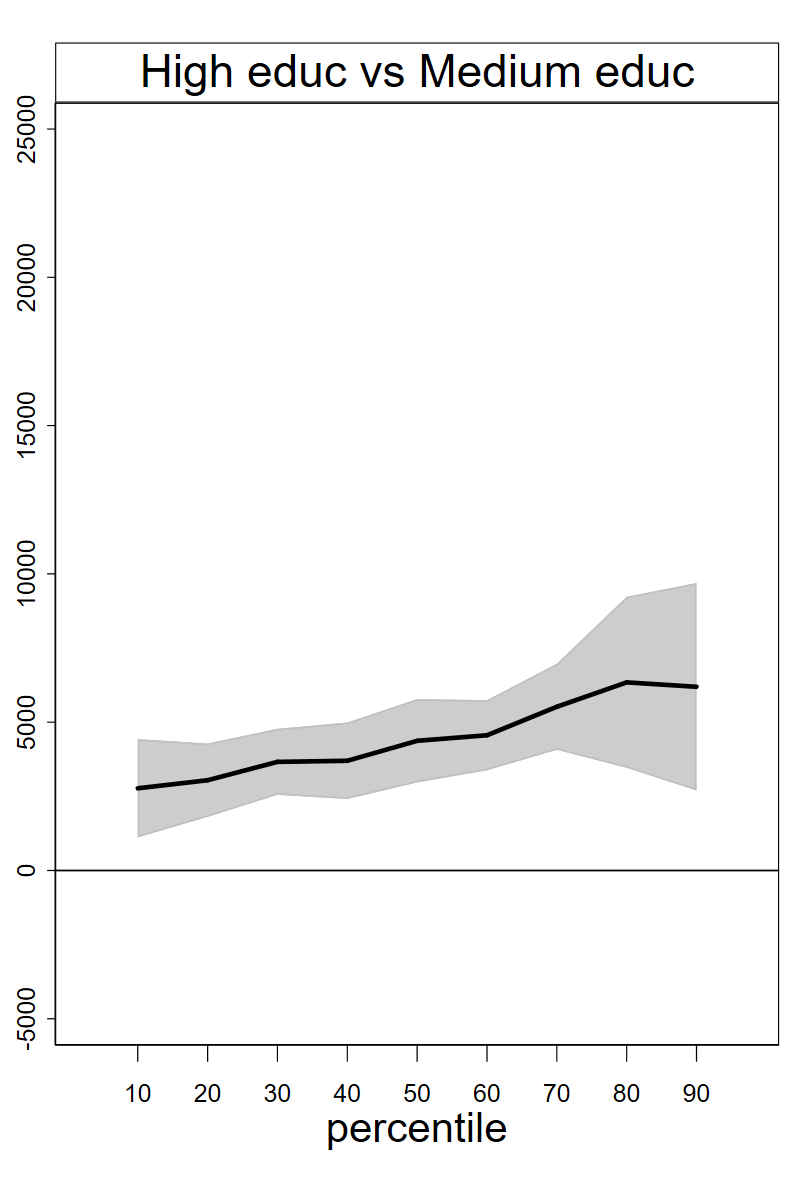

Supplement: Supplementary file 1 [file mmc1.zip › Data_in_Brief/output/graphs/G_1_2_2011_15.png]

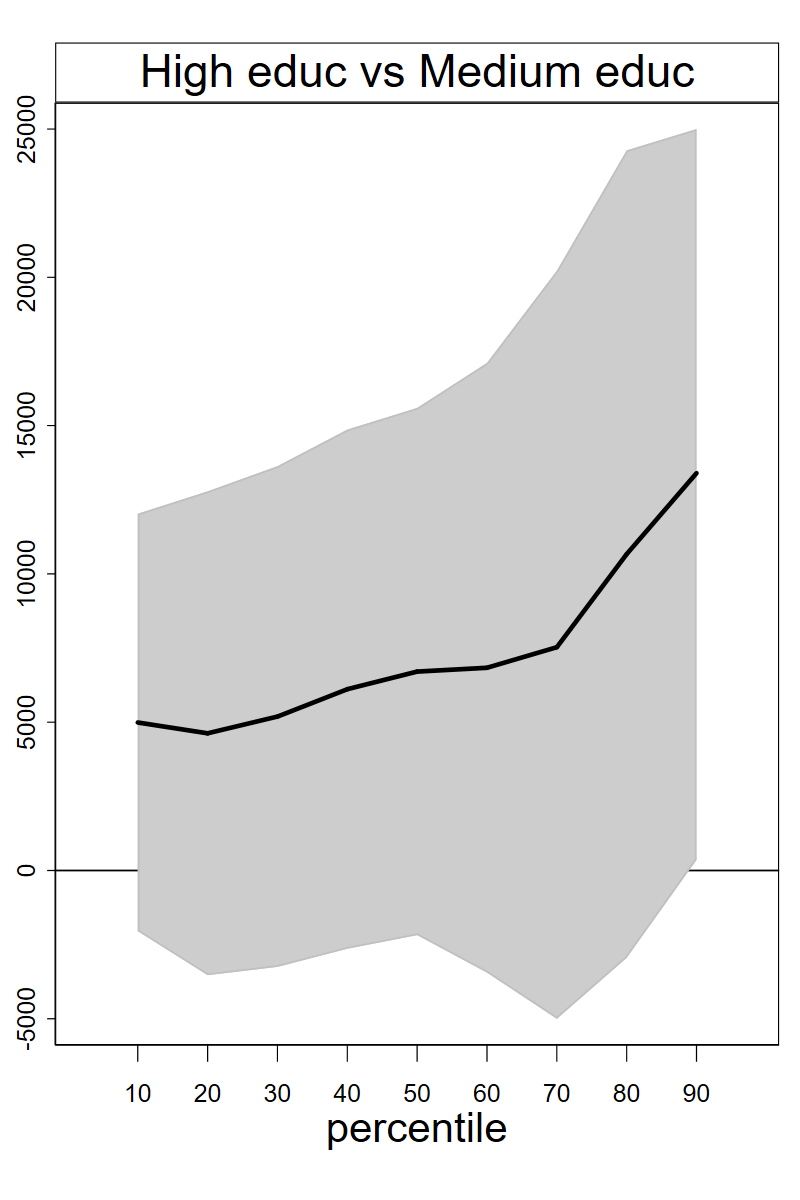

Supplement: Supplementary file 1 [file mmc1.zip › Data_in_Brief/output/graphs/G_1_2_2011_16.png]

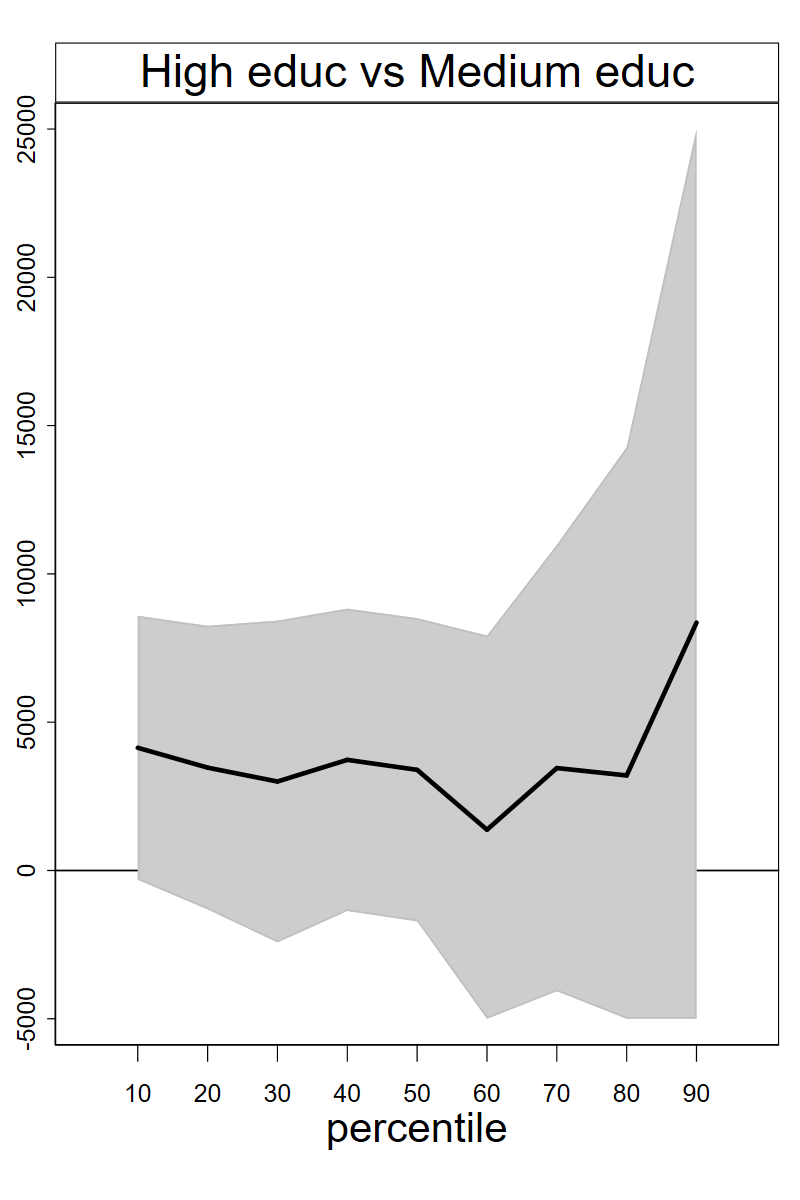

Supplement: Supplementary file 1 [file mmc1.zip › Data_in_Brief/output/graphs/G_1_2_2011_17.png]

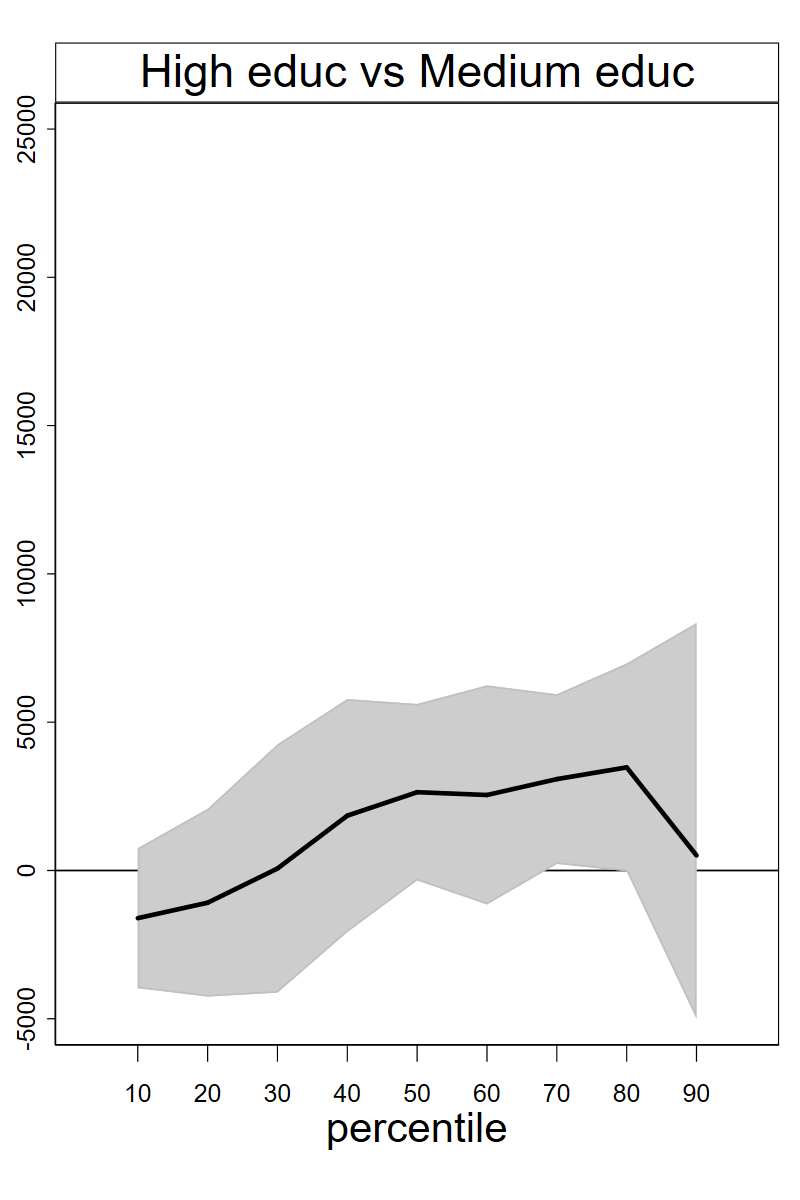

Supplement: Supplementary file 1 [file mmc1.zip › Data_in_Brief/output/graphs/G_1_2_2011_19.png]

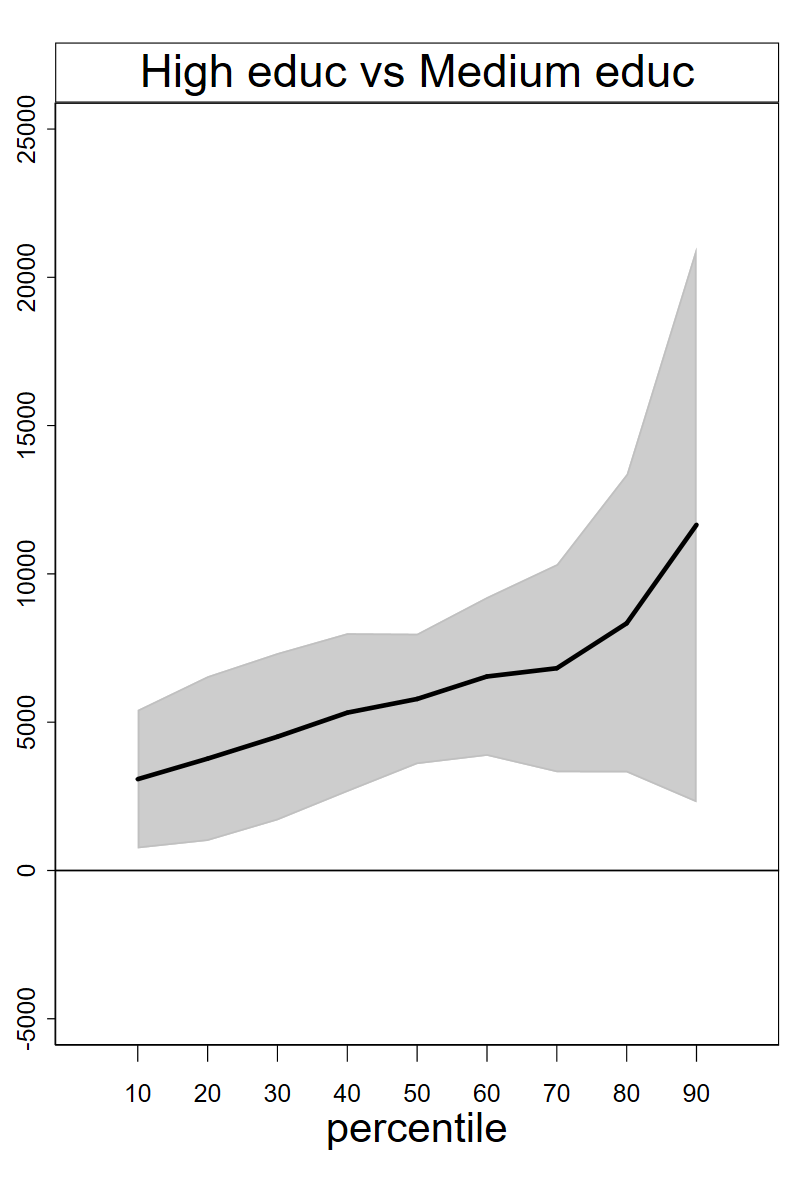

Supplement: Supplementary file 1 [file mmc1.zip › Data_in_Brief/output/graphs/G_1_2_2011_2.png]

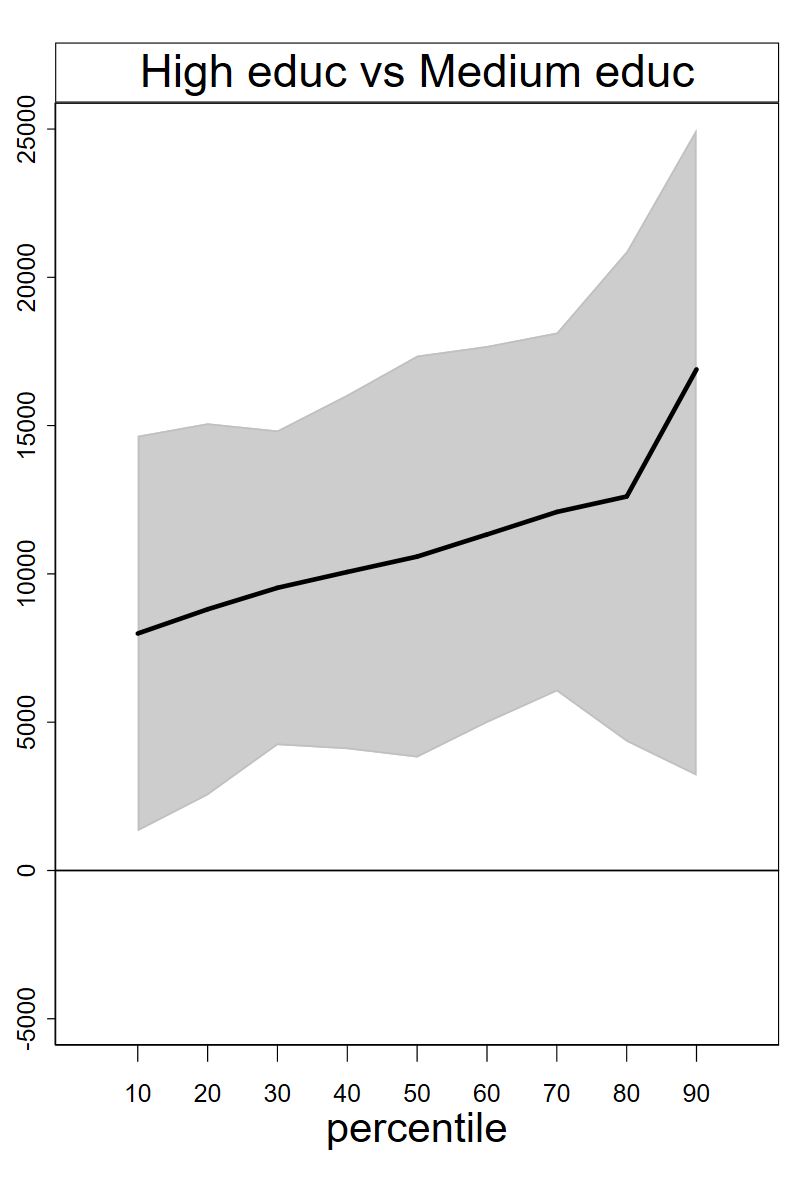

Supplement: Supplementary file 1 [file mmc1.zip › Data_in_Brief/output/graphs/G_1_2_2011_20.png]

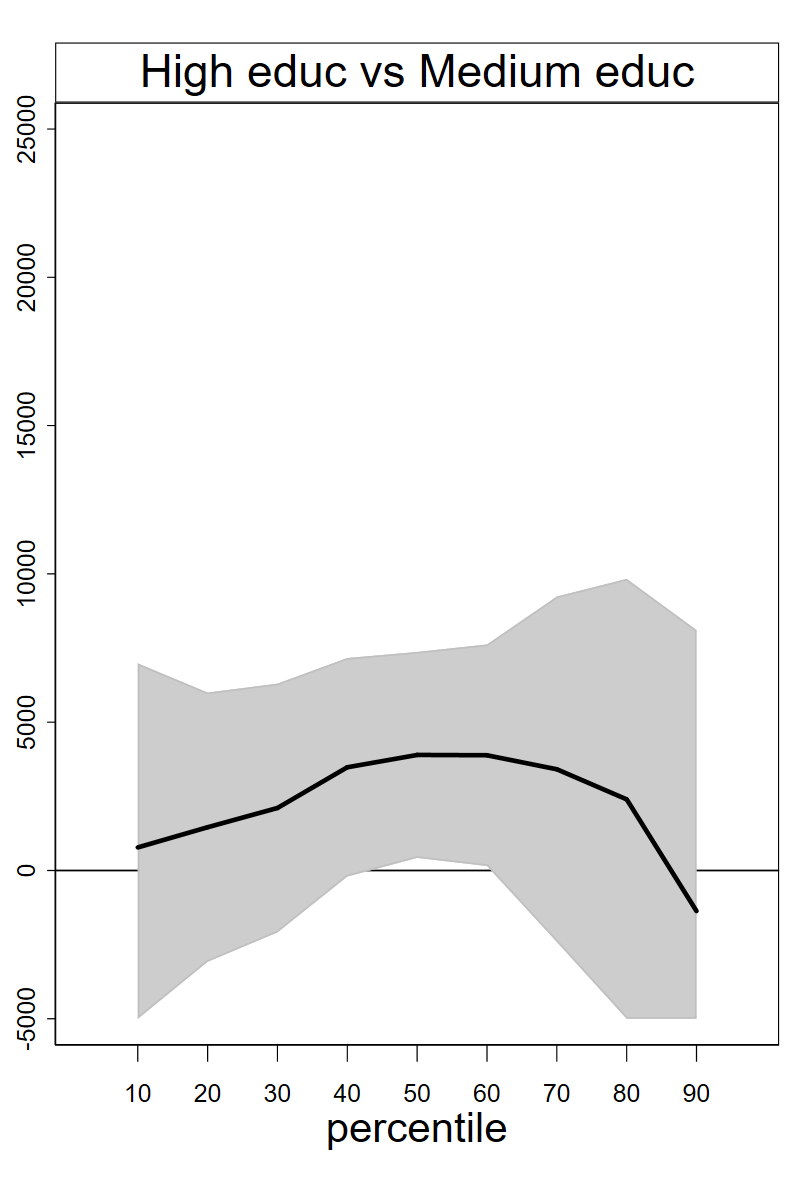

Supplement: Supplementary file 1 [file mmc1.zip › Data_in_Brief/output/graphs/G_1_2_2011_23.png]

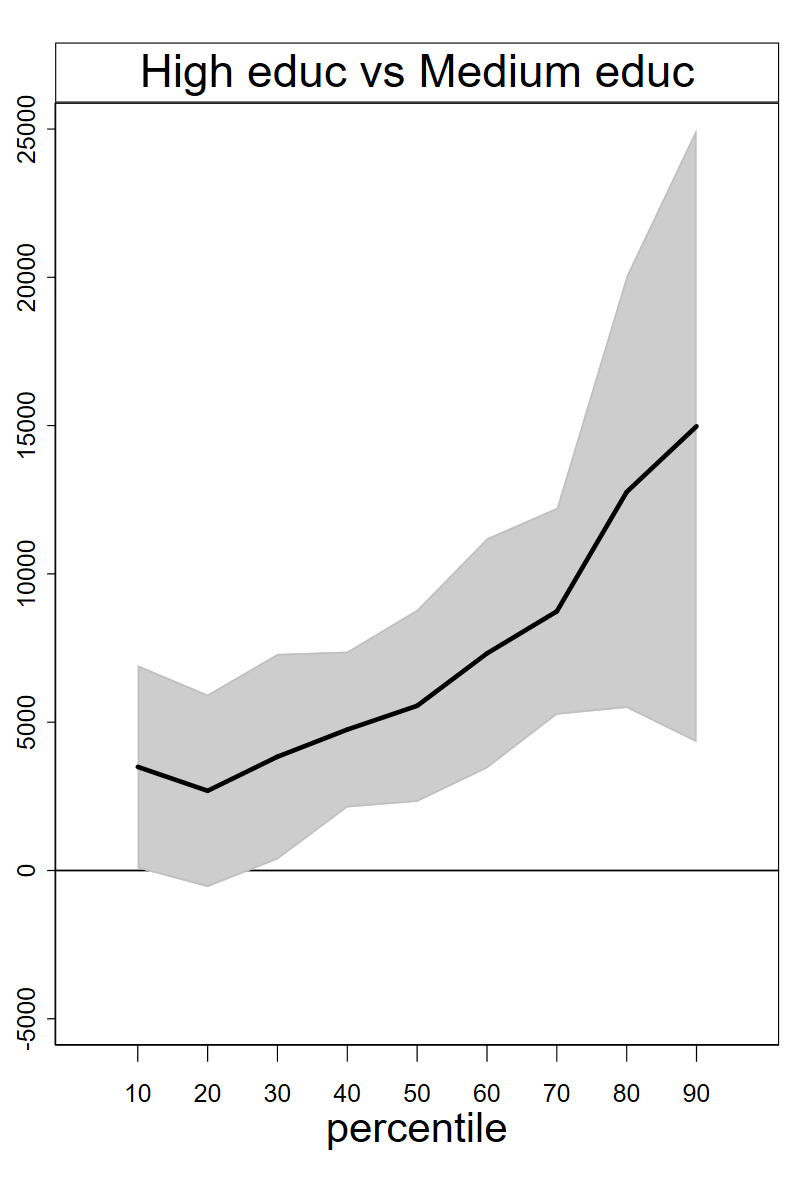

Supplement: Supplementary file 1 [file mmc1.zip › Data_in_Brief/output/graphs/G_1_2_2011_24.png]

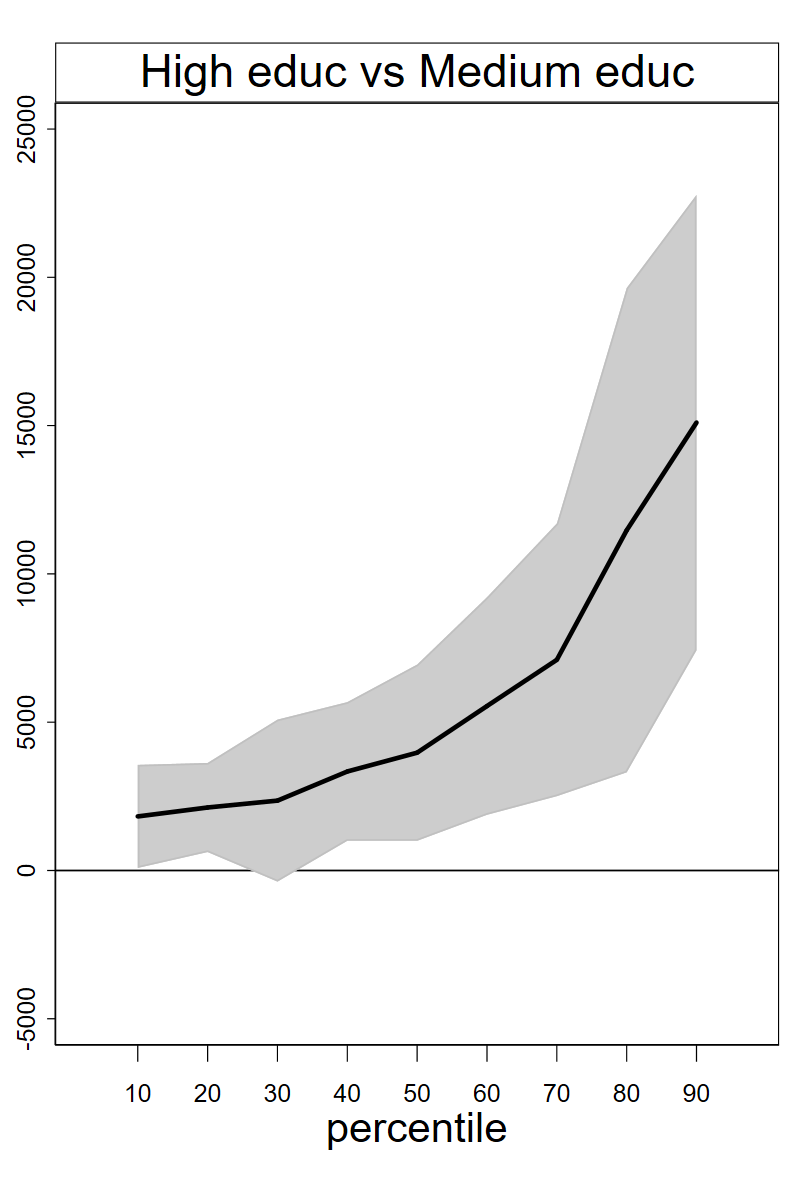

Supplement: Supplementary file 1 [file mmc1.zip › Data_in_Brief/output/graphs/G_1_2_2011_25.png]

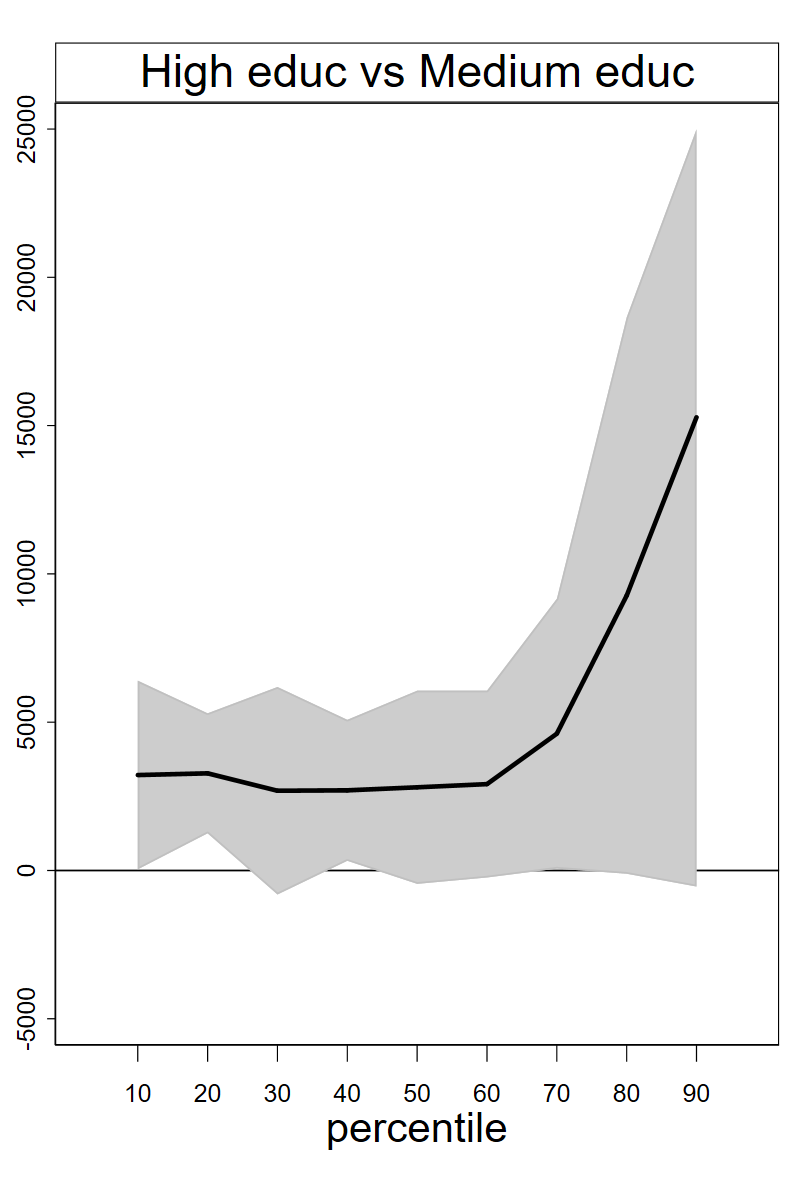

Supplement: Supplementary file 1 [file mmc1.zip › Data_in_Brief/output/graphs/G_1_2_2011_28.png]

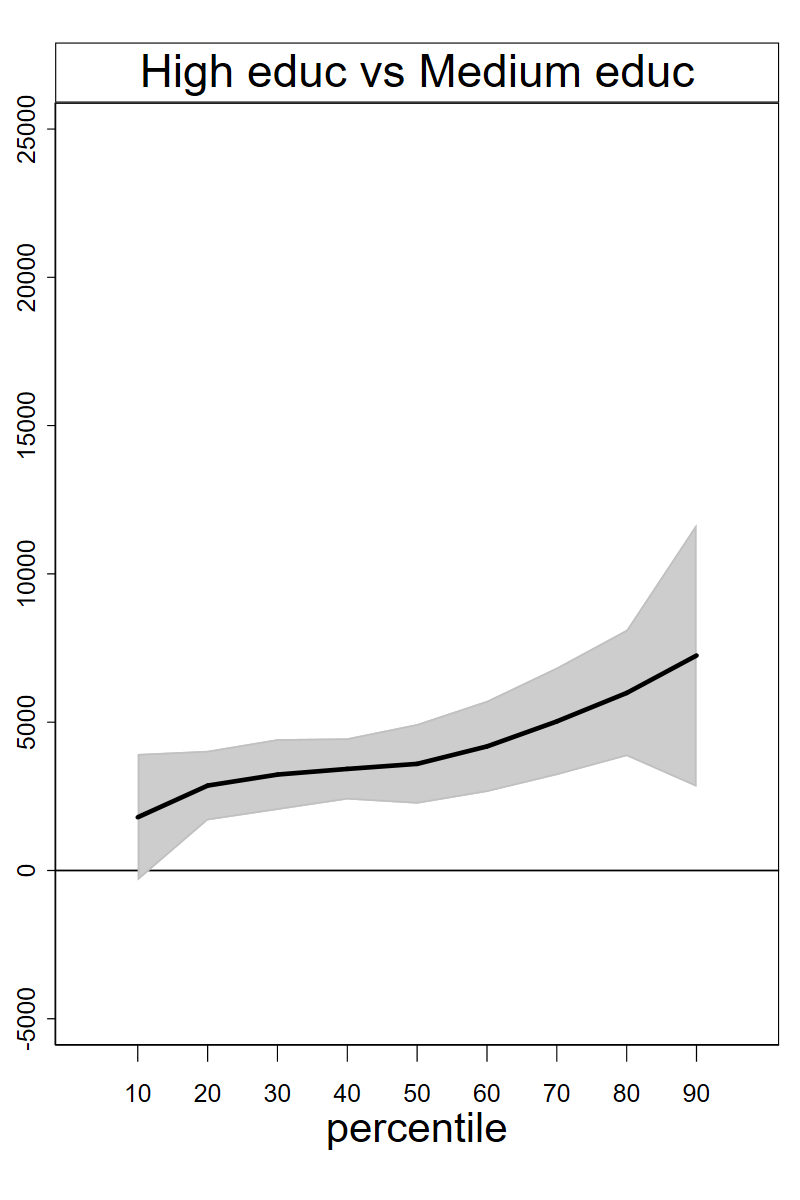

Supplement: Supplementary file 1 [file mmc1.zip › Data_in_Brief/output/graphs/G_1_2_2011_30.png]

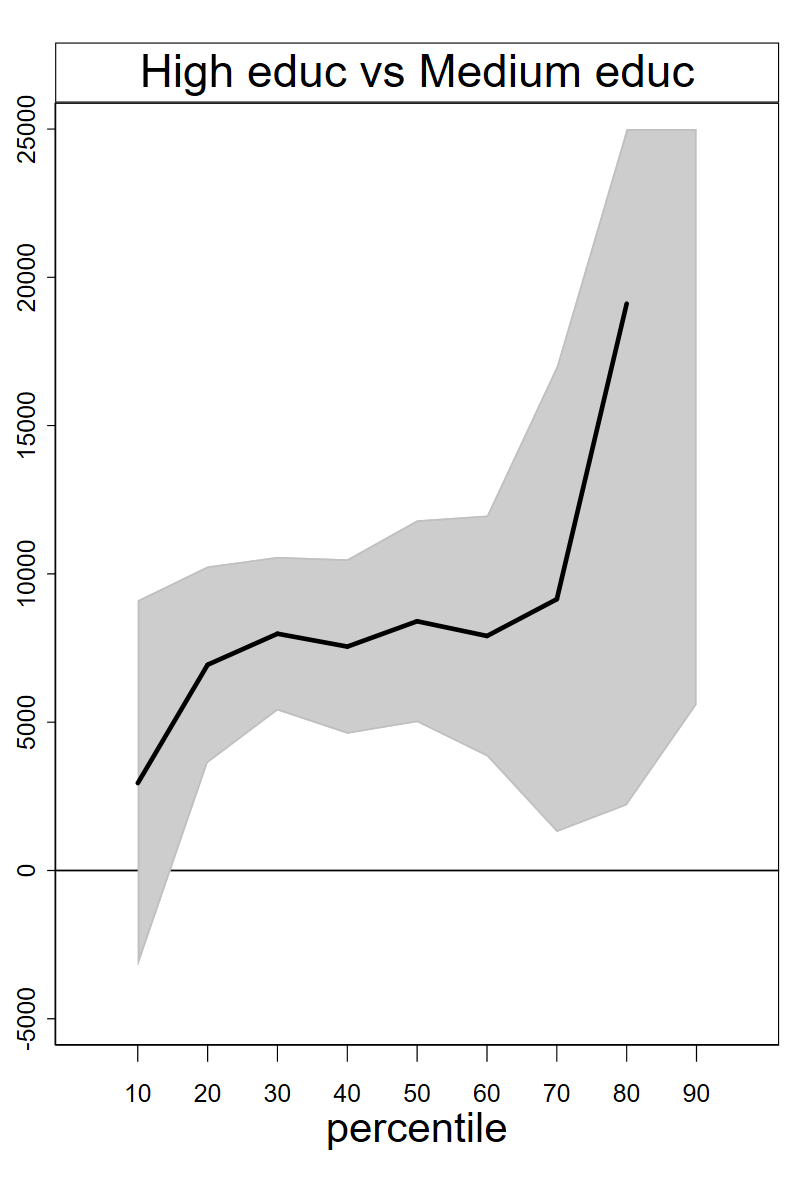

Supplement: Supplementary file 1 [file mmc1.zip › Data_in_Brief/output/graphs/G_1_2_2011_31.png]

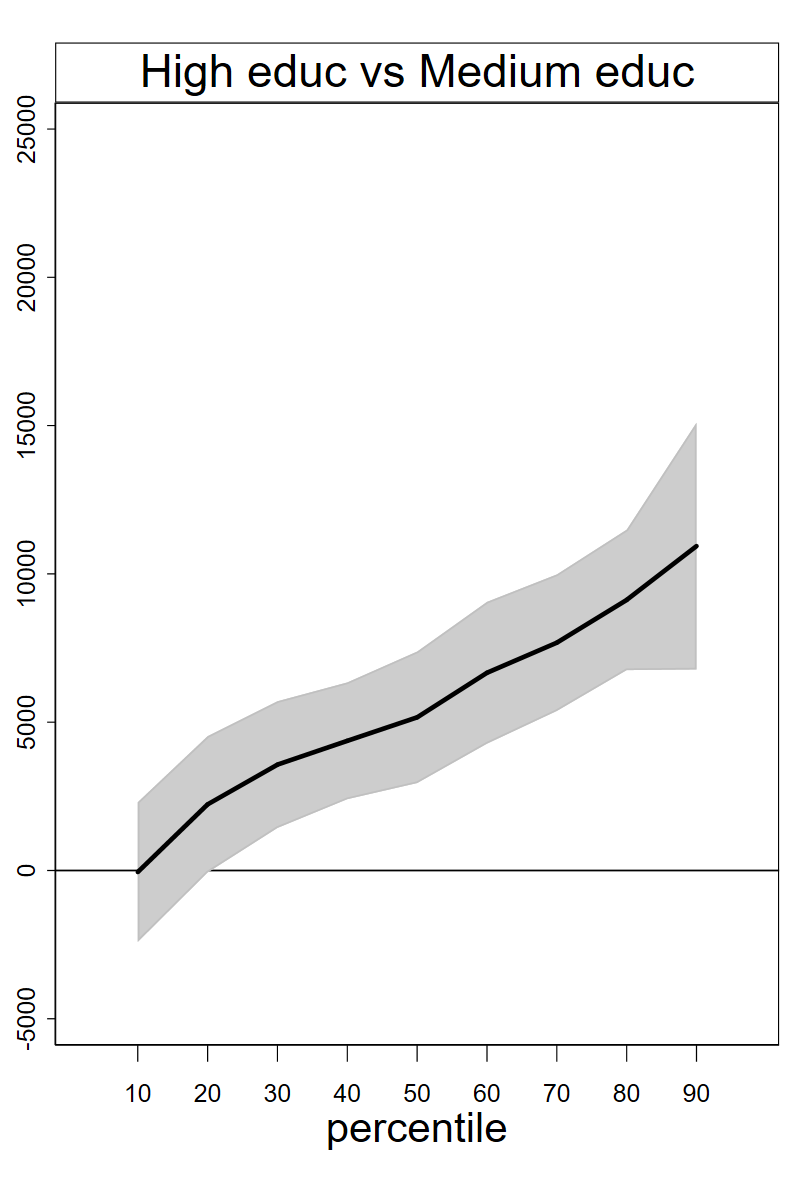

Supplement: Supplementary file 1 [file mmc1.zip › Data_in_Brief/output/graphs/G_1_2_2011_7.png]

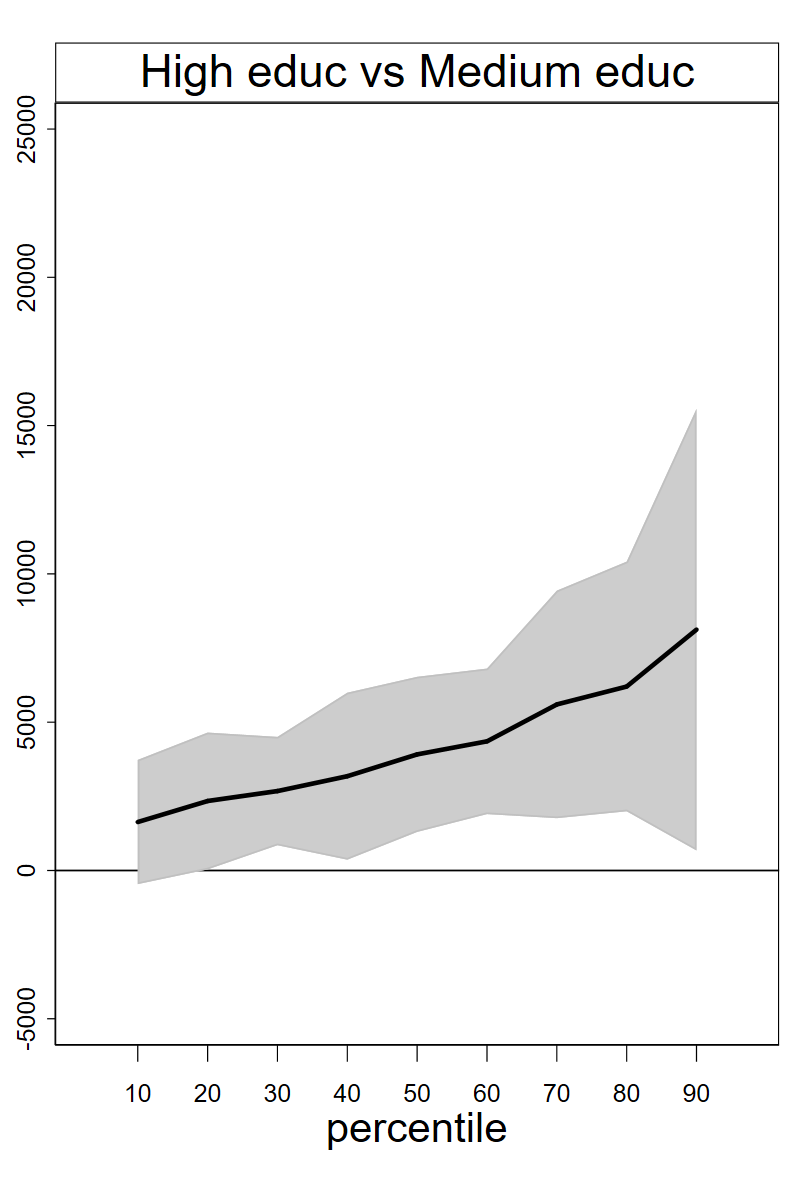

Supplement: Supplementary file 1 [file mmc1.zip › Data_in_Brief/output/graphs/G_1_2_2011_9.png]

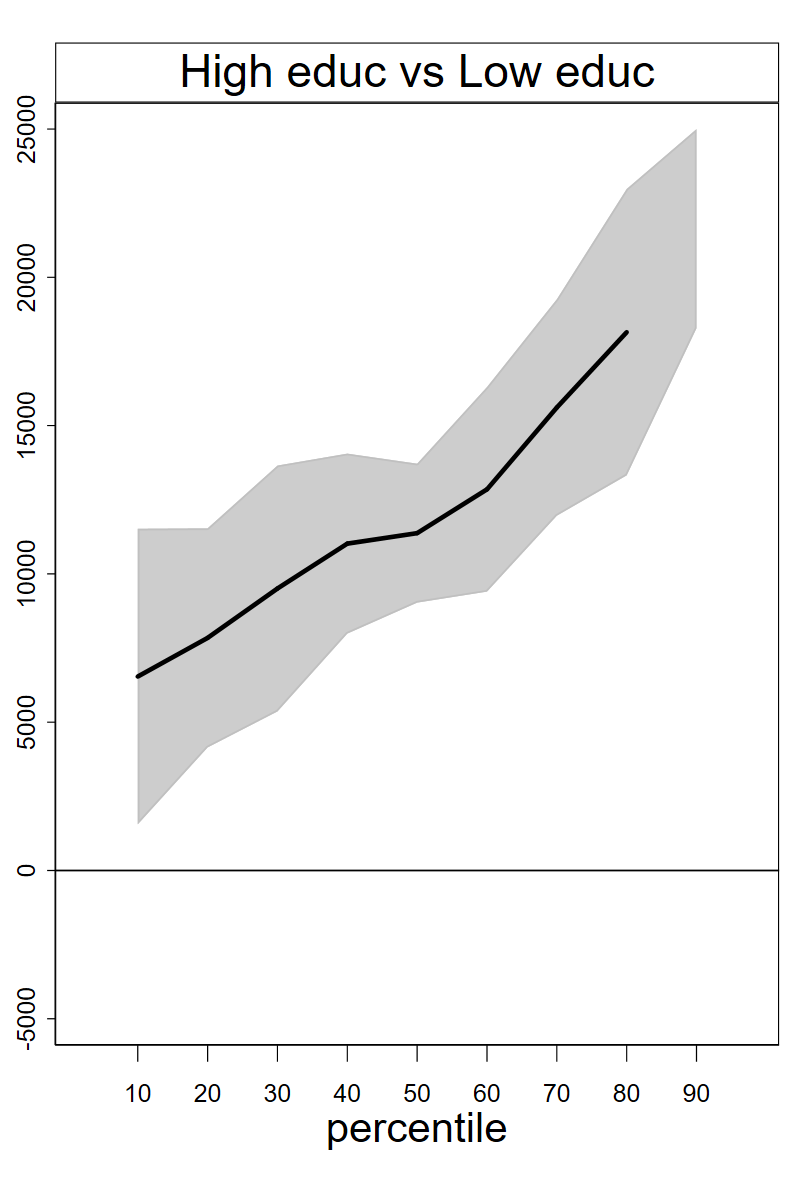

Supplement: Supplementary file 1 [file mmc1.zip › Data_in_Brief/output/graphs/G_1_3_2011_1.png]

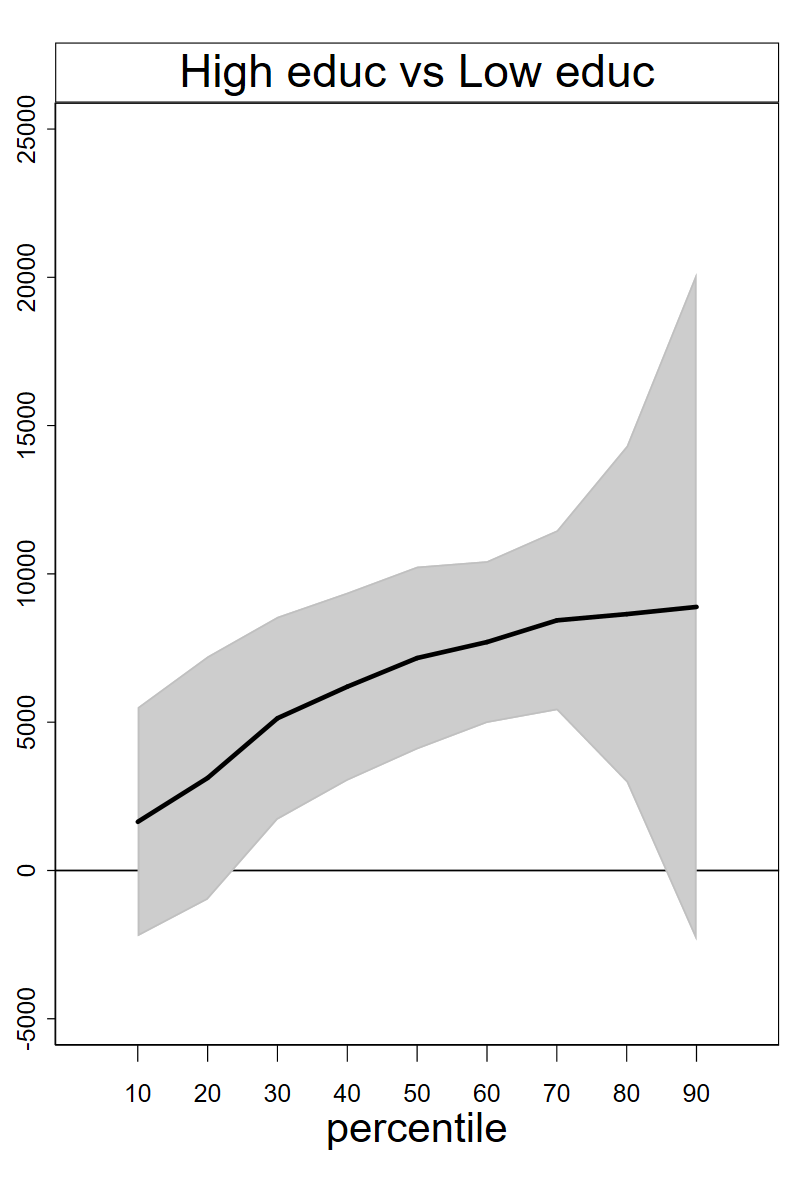

Supplement: Supplementary file 1 [file mmc1.zip › Data_in_Brief/output/graphs/G_1_3_2011_12.png]

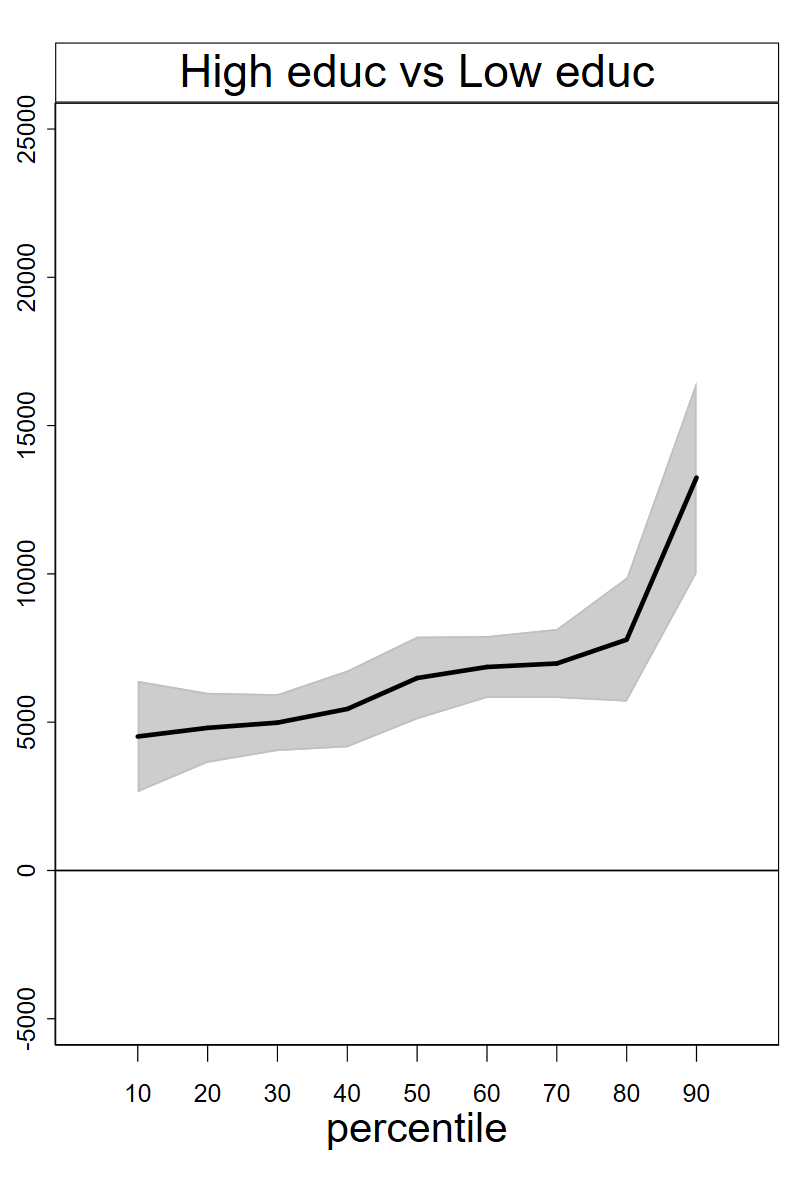

Supplement: Supplementary file 1 [file mmc1.zip › Data_in_Brief/output/graphs/G_1_3_2011_15.png]

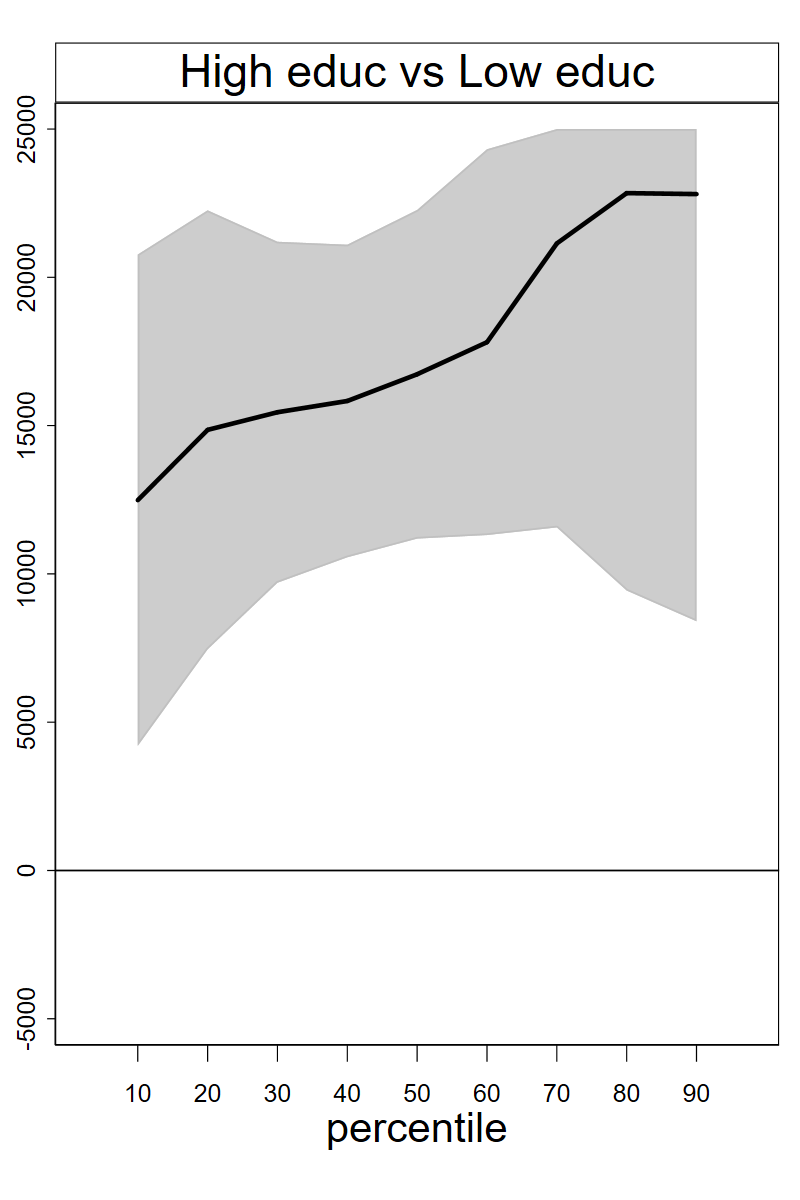

Supplement: Supplementary file 1 [file mmc1.zip › Data_in_Brief/output/graphs/G_1_3_2011_16.png]

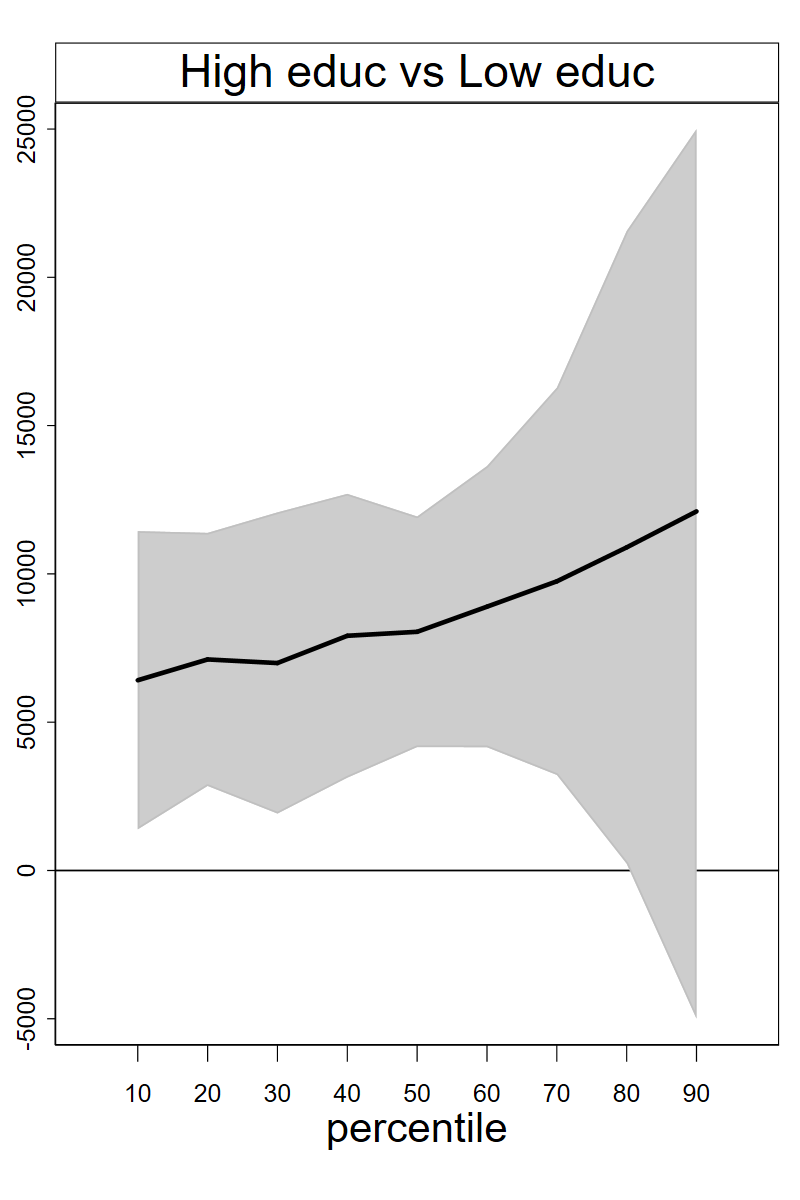

Supplement: Supplementary file 1 [file mmc1.zip › Data_in_Brief/output/graphs/G_1_3_2011_17.png]

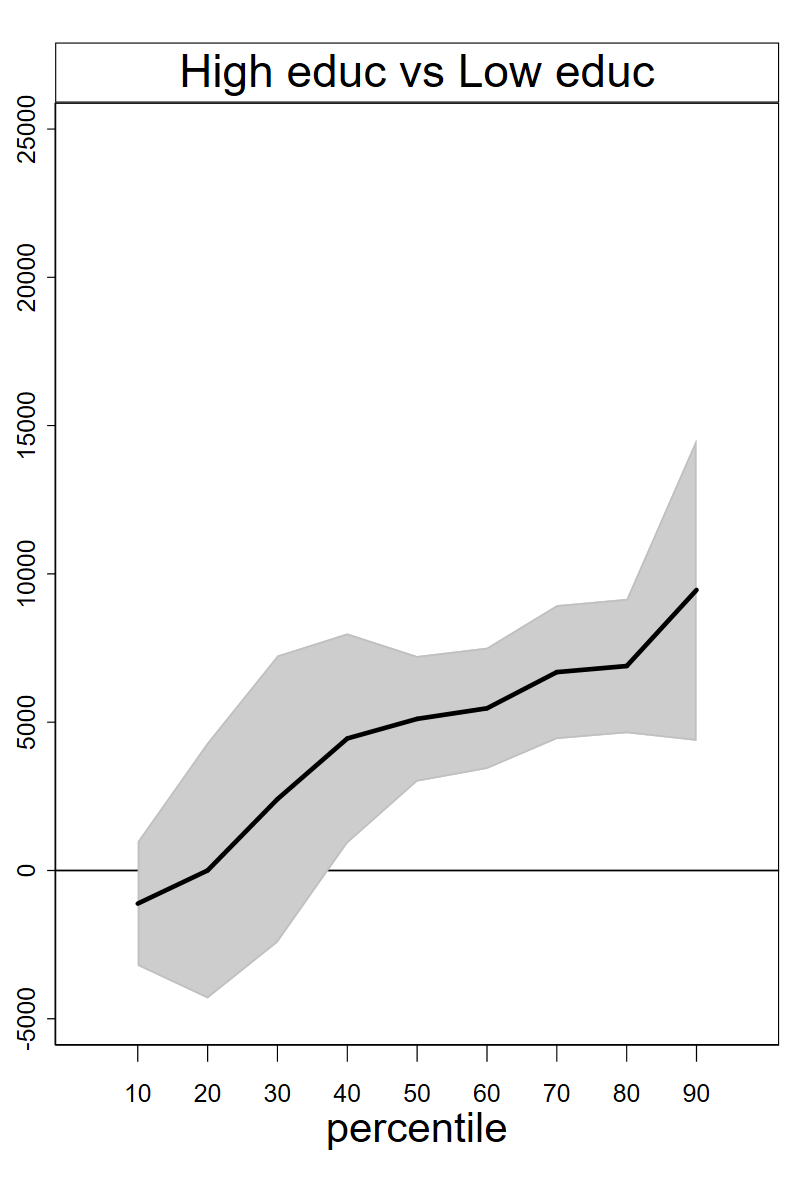

Supplement: Supplementary file 1 [file mmc1.zip › Data_in_Brief/output/graphs/G_1_3_2011_19.png]

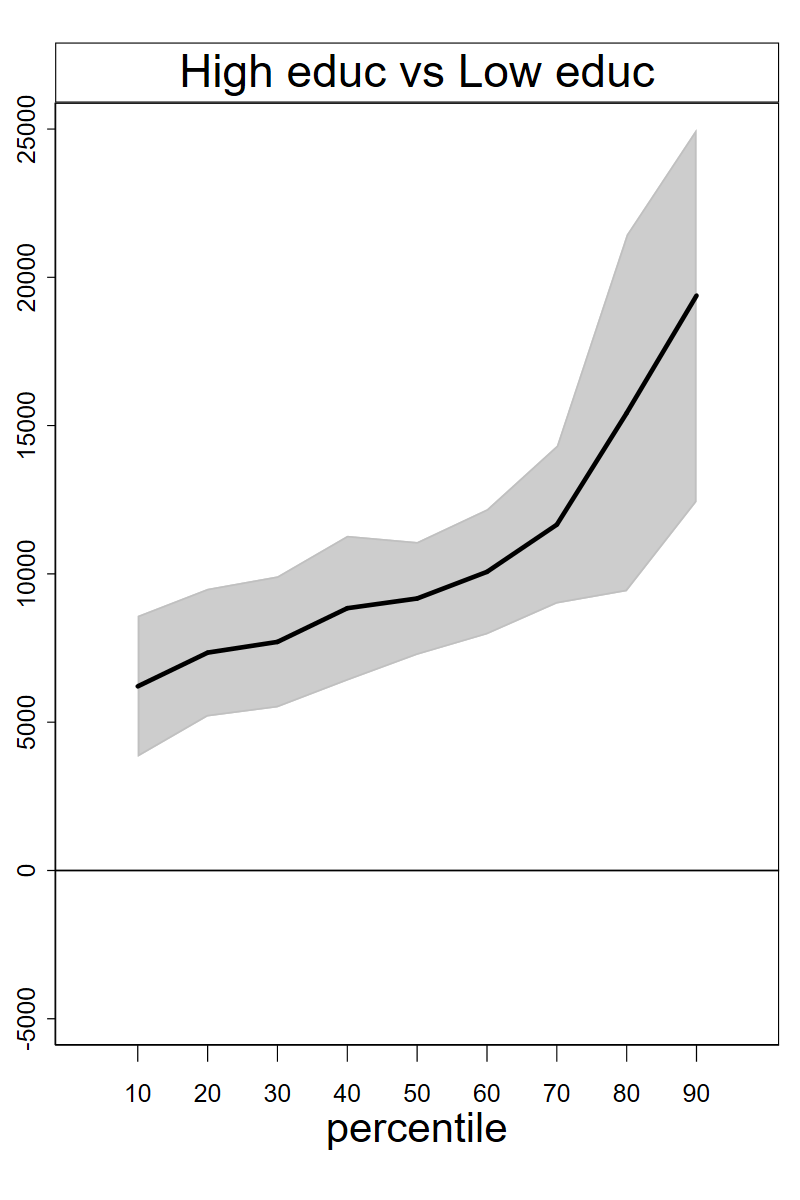

Supplement: Supplementary file 1 [file mmc1.zip › Data_in_Brief/output/graphs/G_1_3_2011_2.png]

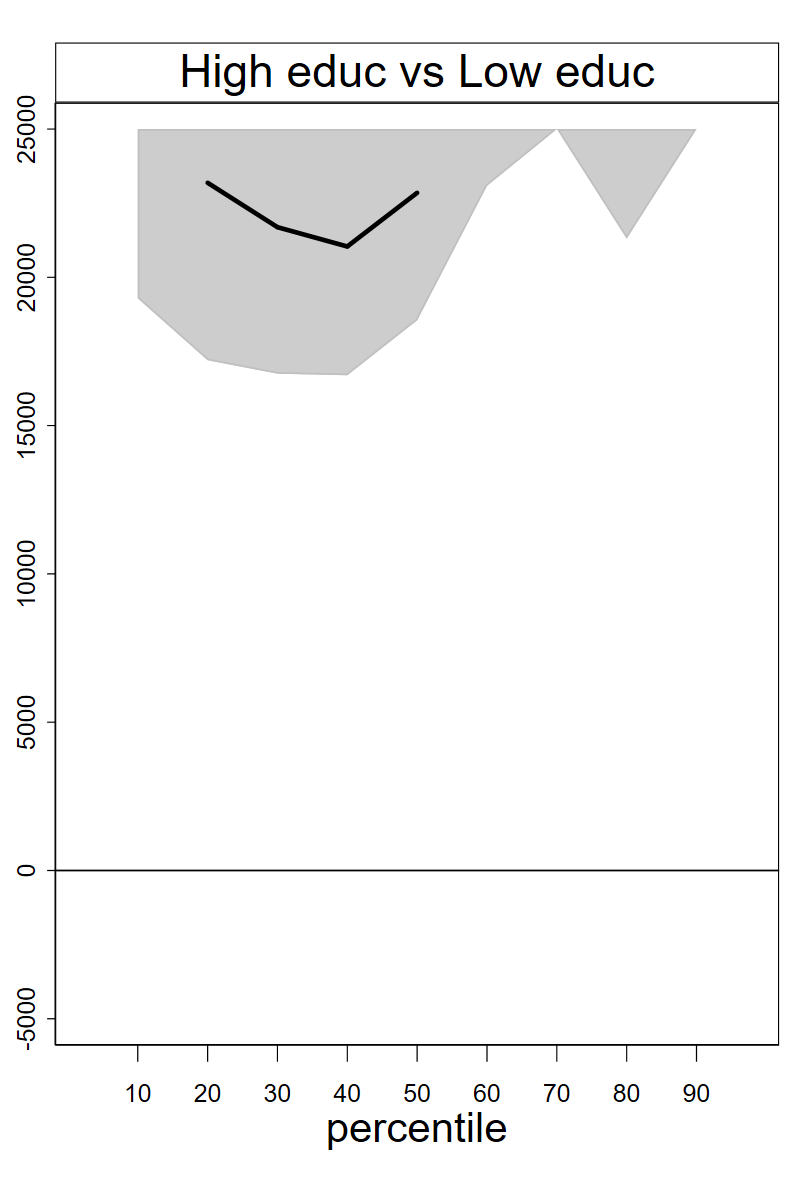

Supplement: Supplementary file 1 [file mmc1.zip › Data_in_Brief/output/graphs/G_1_3_2011_20.png]

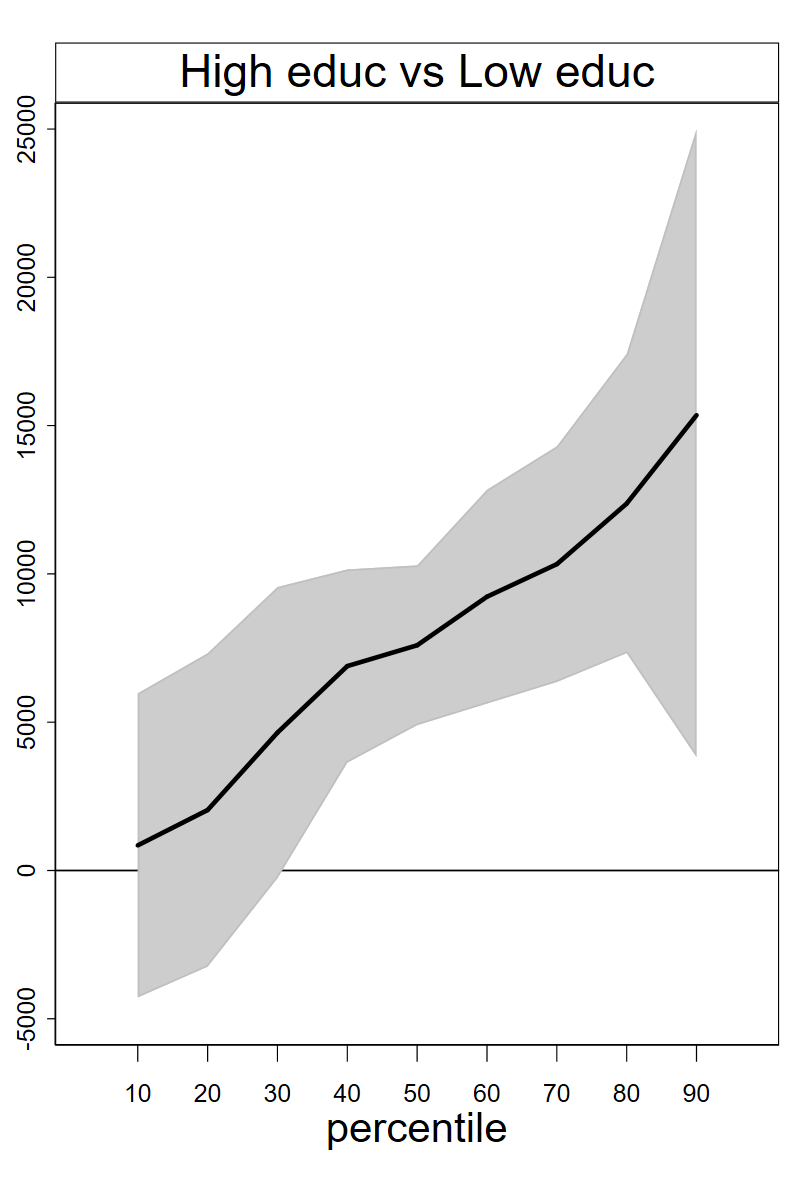

Supplement: Supplementary file 1 [file mmc1.zip › Data_in_Brief/output/graphs/G_1_3_2011_23.png]

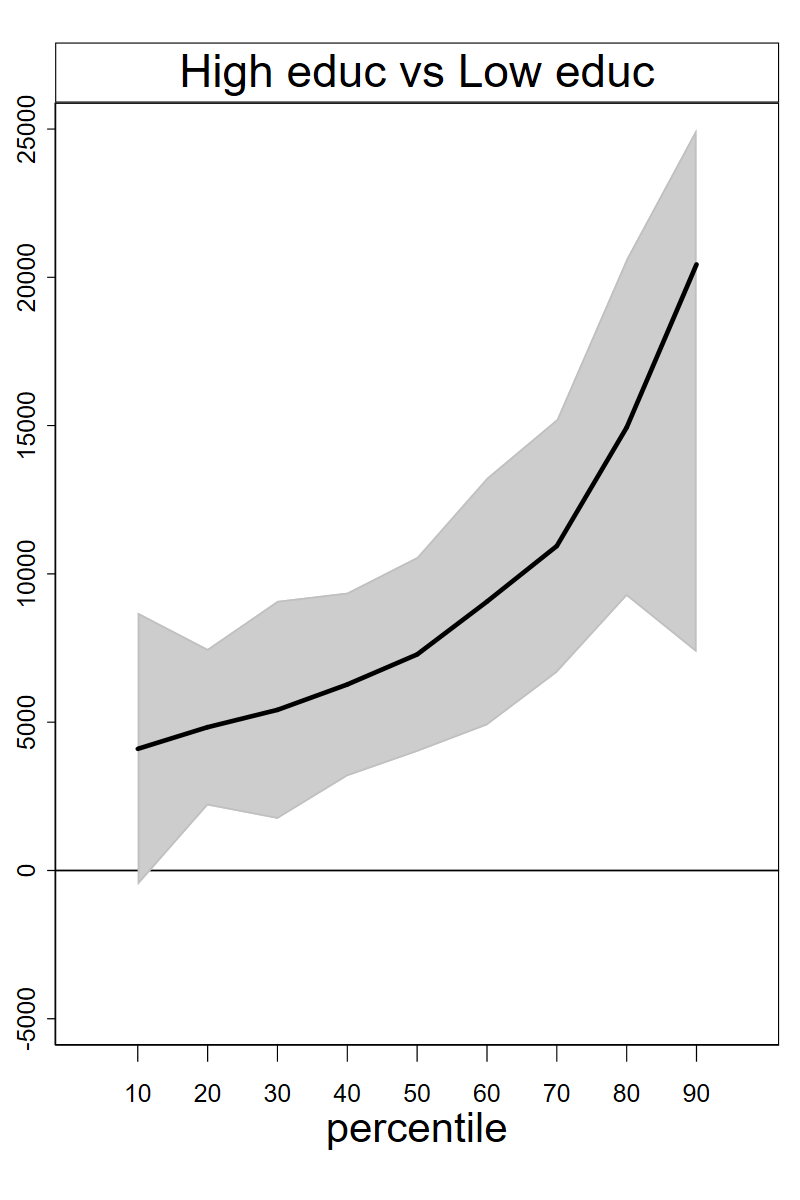

Supplement: Supplementary file 1 [file mmc1.zip › Data_in_Brief/output/graphs/G_1_3_2011_24.png]

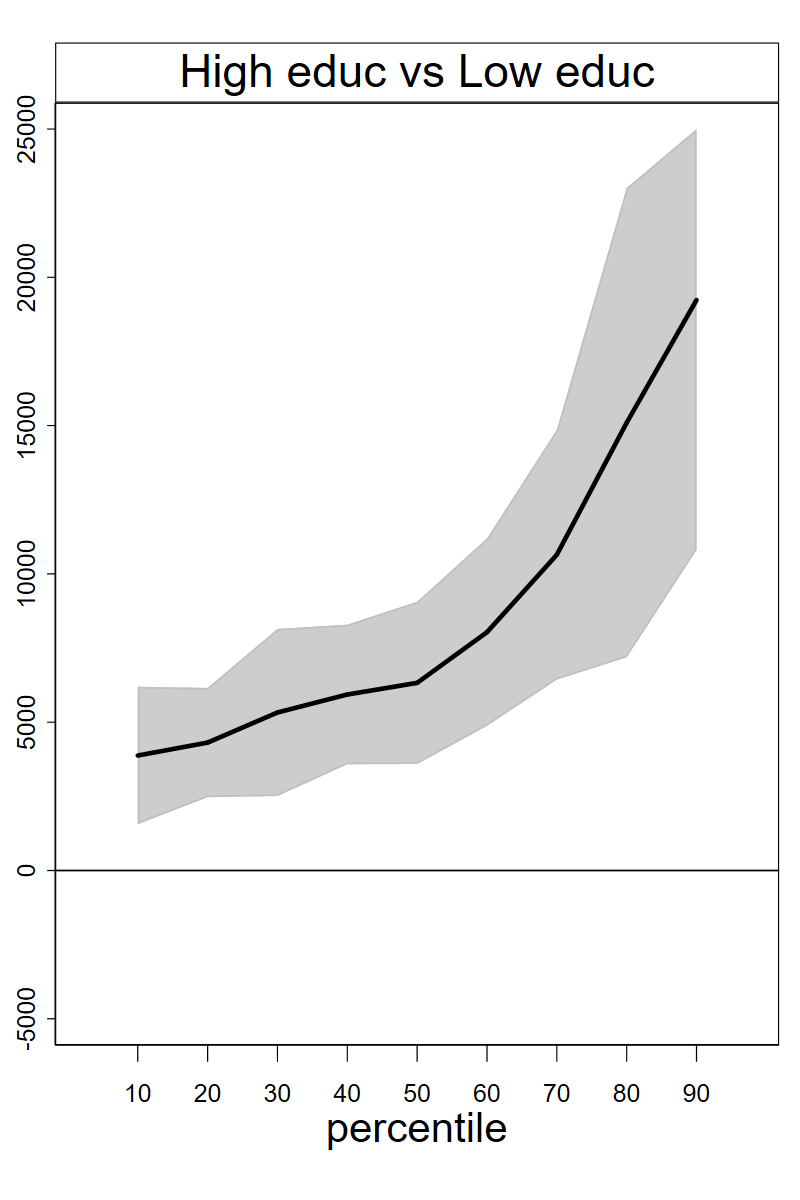

Supplement: Supplementary file 1 [file mmc1.zip › Data_in_Brief/output/graphs/G_1_3_2011_25.png]

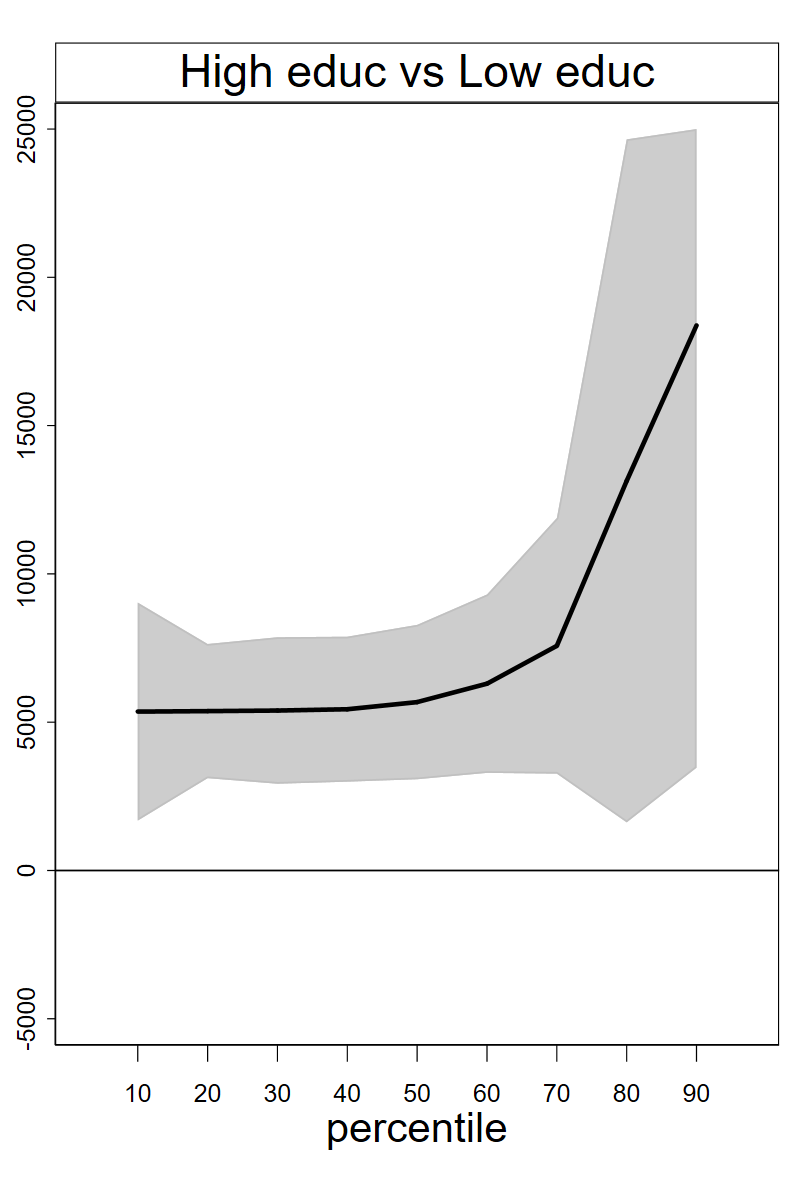

Supplement: Supplementary file 1 [file mmc1.zip › Data_in_Brief/output/graphs/G_1_3_2011_28.png]

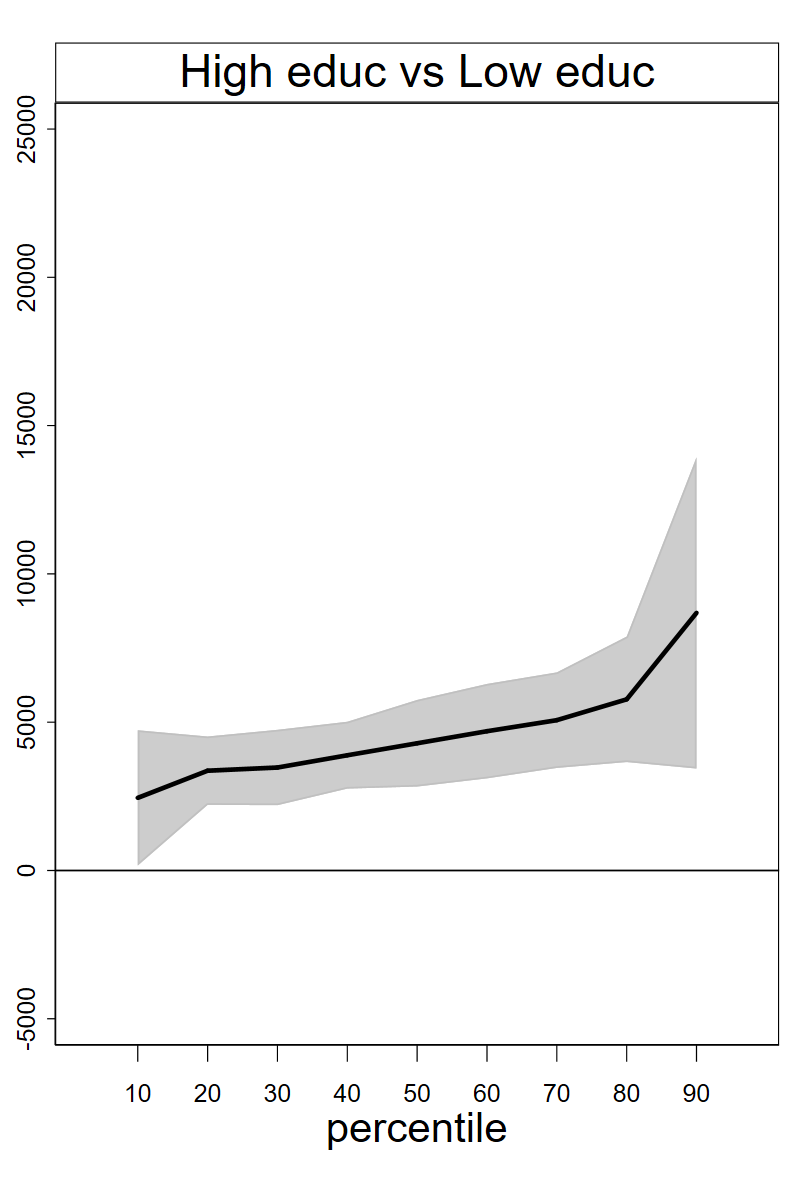

Supplement: Supplementary file 1 [file mmc1.zip › Data_in_Brief/output/graphs/G_1_3_2011_30.png]

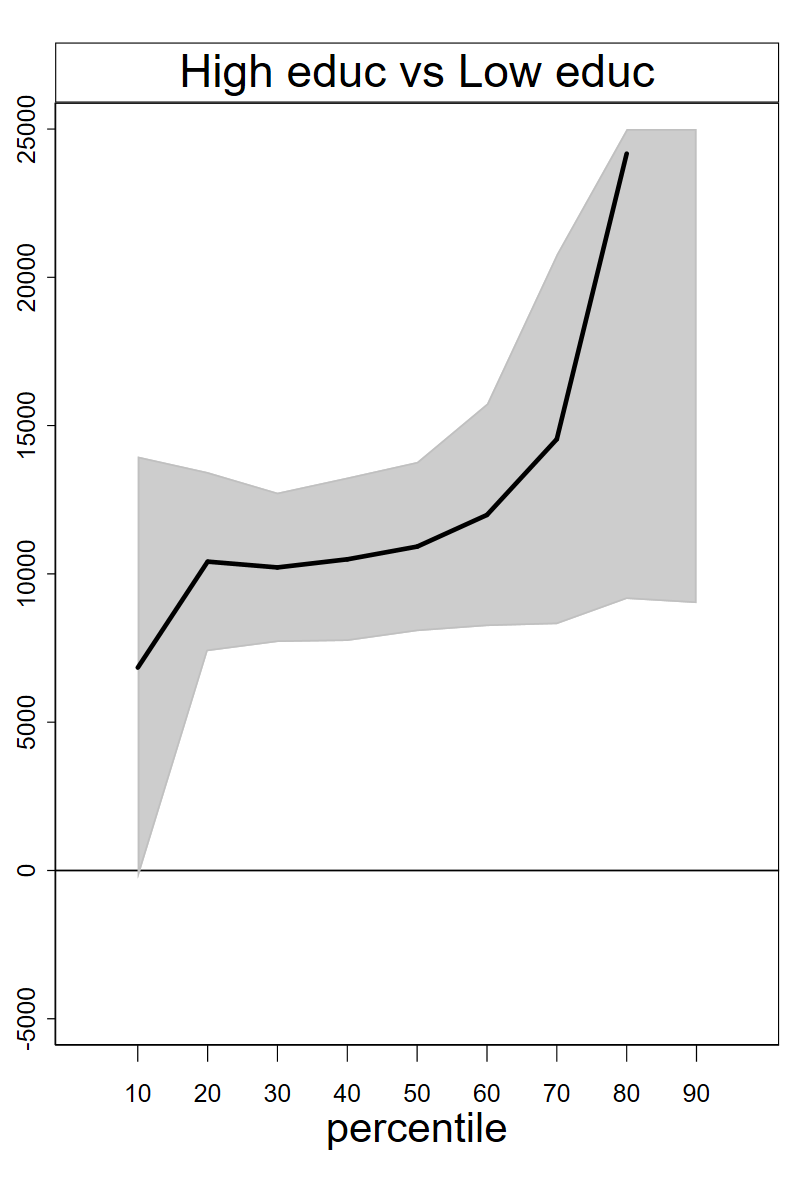

Supplement: Supplementary file 1 [file mmc1.zip › Data_in_Brief/output/graphs/G_1_3_2011_31.png]

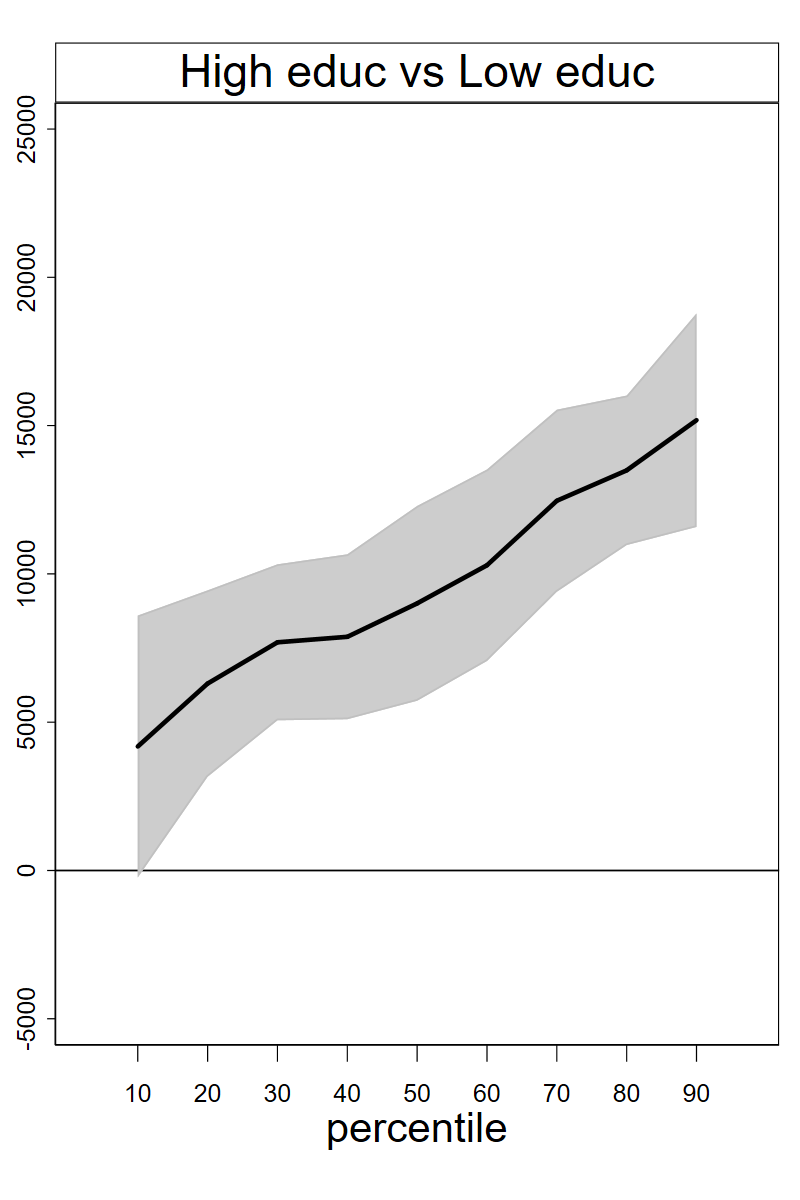

Supplement: Supplementary file 1 [file mmc1.zip › Data_in_Brief/output/graphs/G_1_3_2011_7.png]

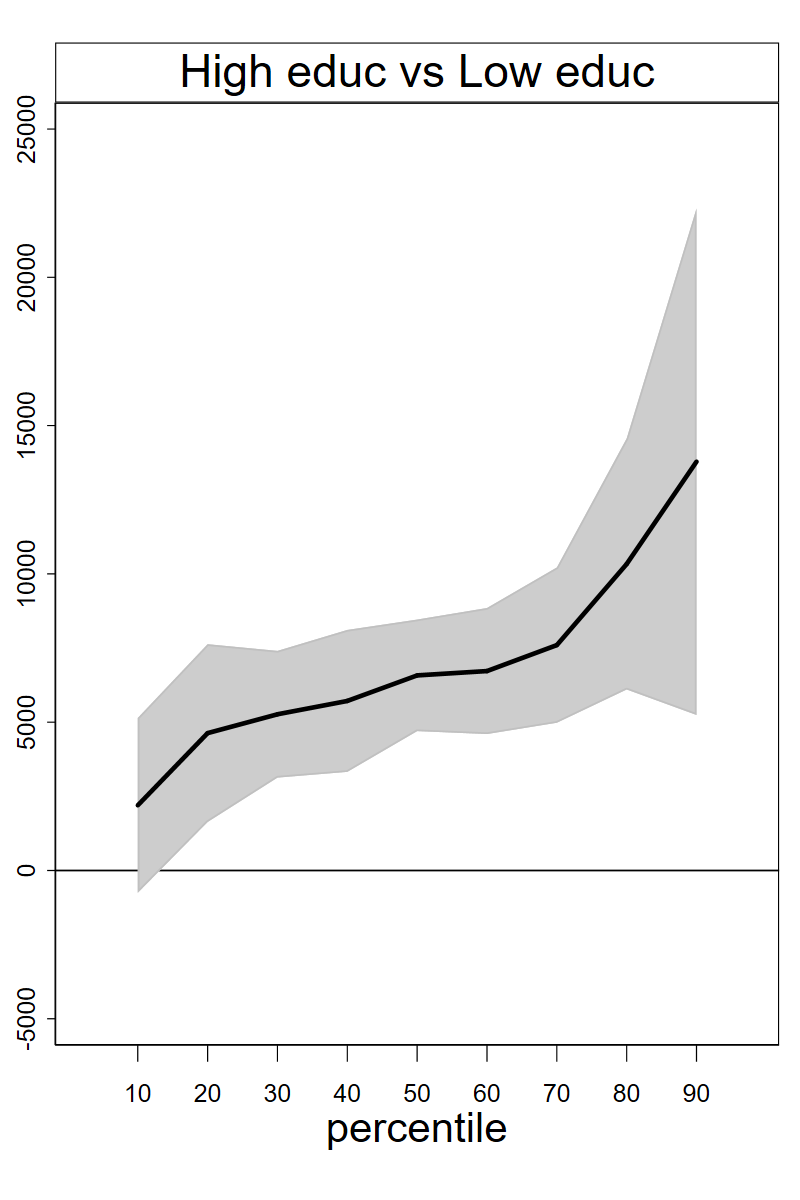

Supplement: Supplementary file 1 [file mmc1.zip › Data_in_Brief/output/graphs/G_1_3_2011_9.png]

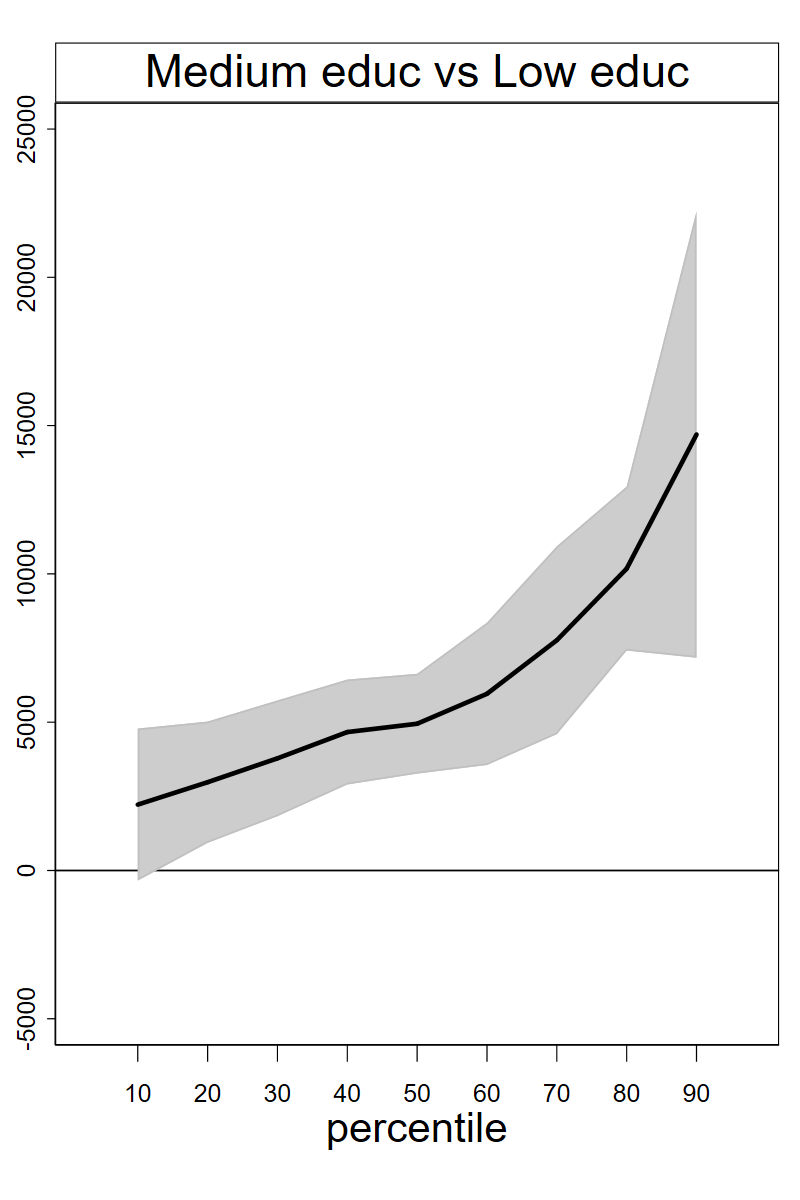

Supplement: Supplementary file 1 [file mmc1.zip › Data_in_Brief/output/graphs/G_2_3_2011_1.png]

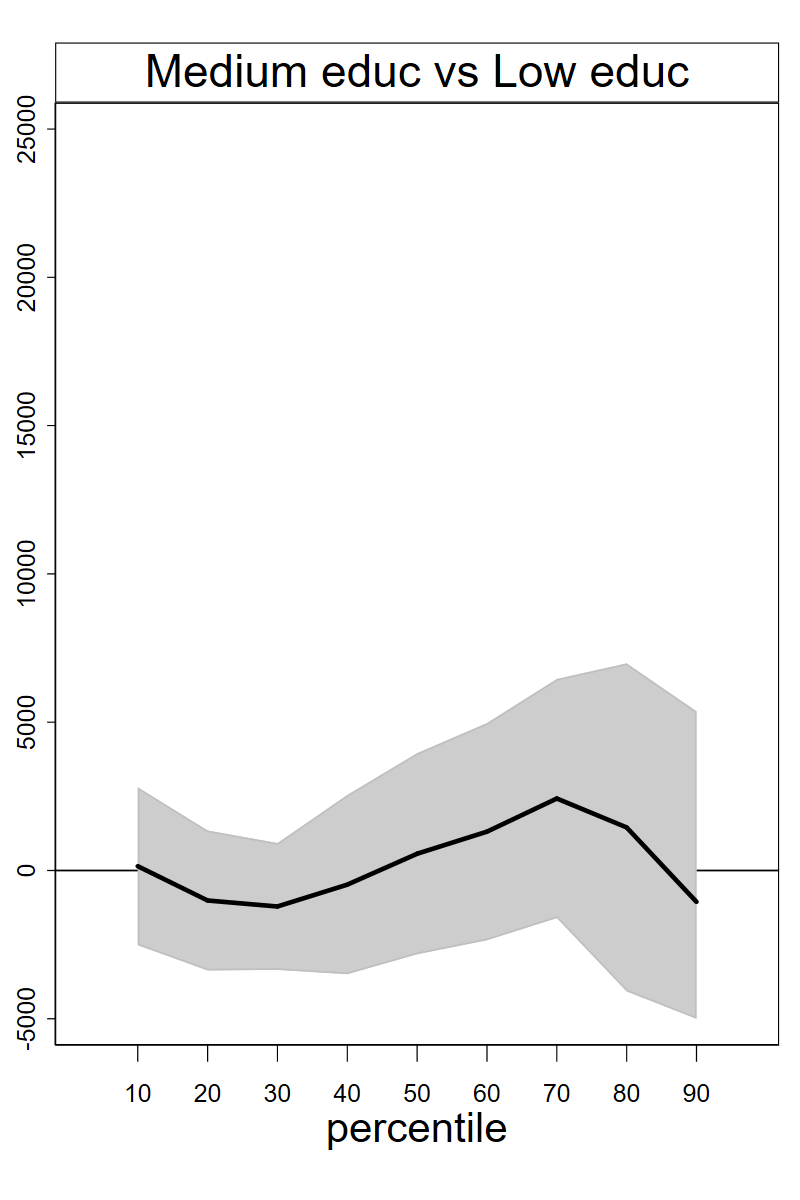

Supplement: Supplementary file 1 [file mmc1.zip › Data_in_Brief/output/graphs/G_2_3_2011_12.png]

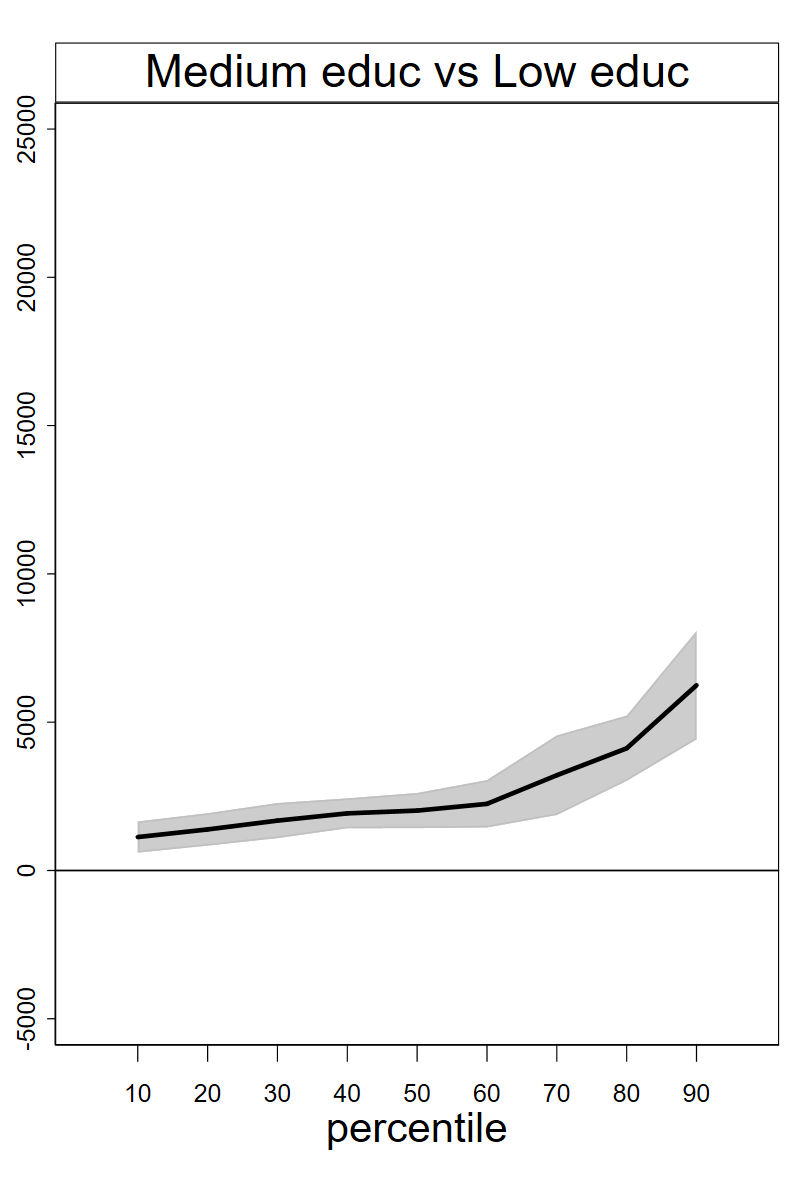

Supplement: Supplementary file 1 [file mmc1.zip › Data_in_Brief/output/graphs/G_2_3_2011_15.png]

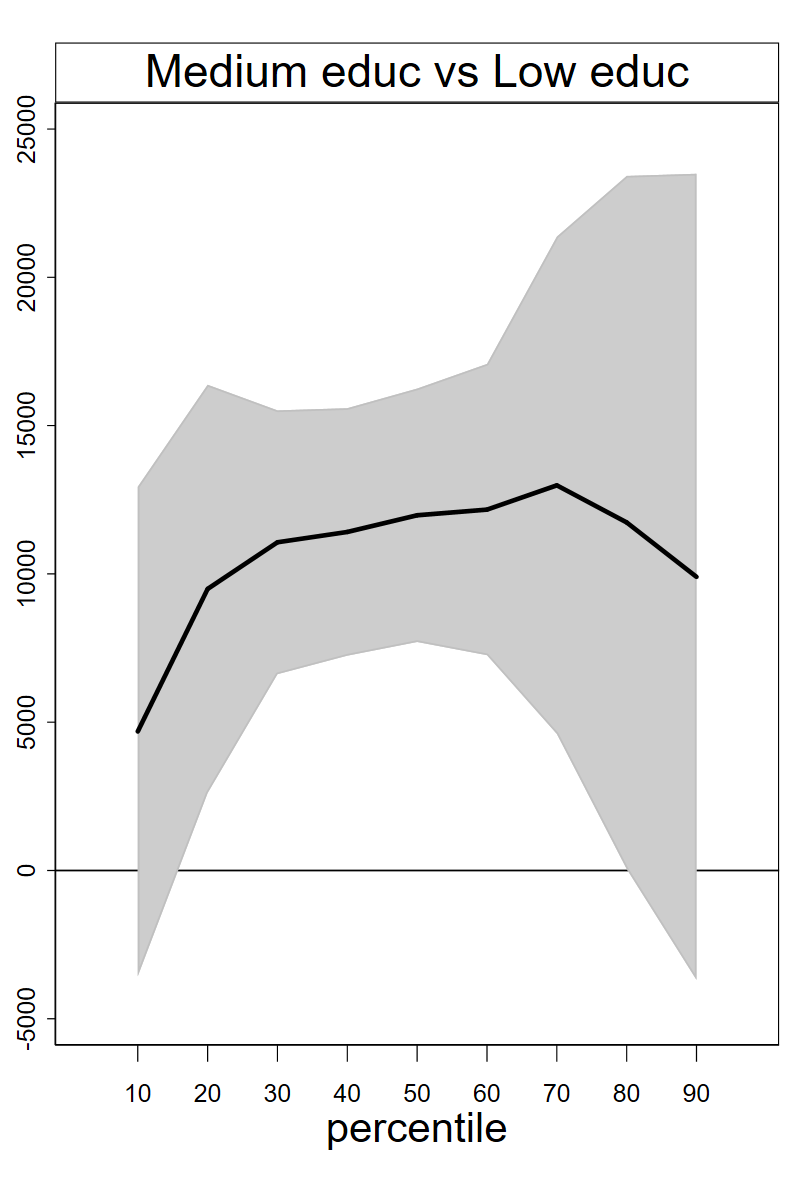

Supplement: Supplementary file 1 [file mmc1.zip › Data_in_Brief/output/graphs/G_2_3_2011_16.png]

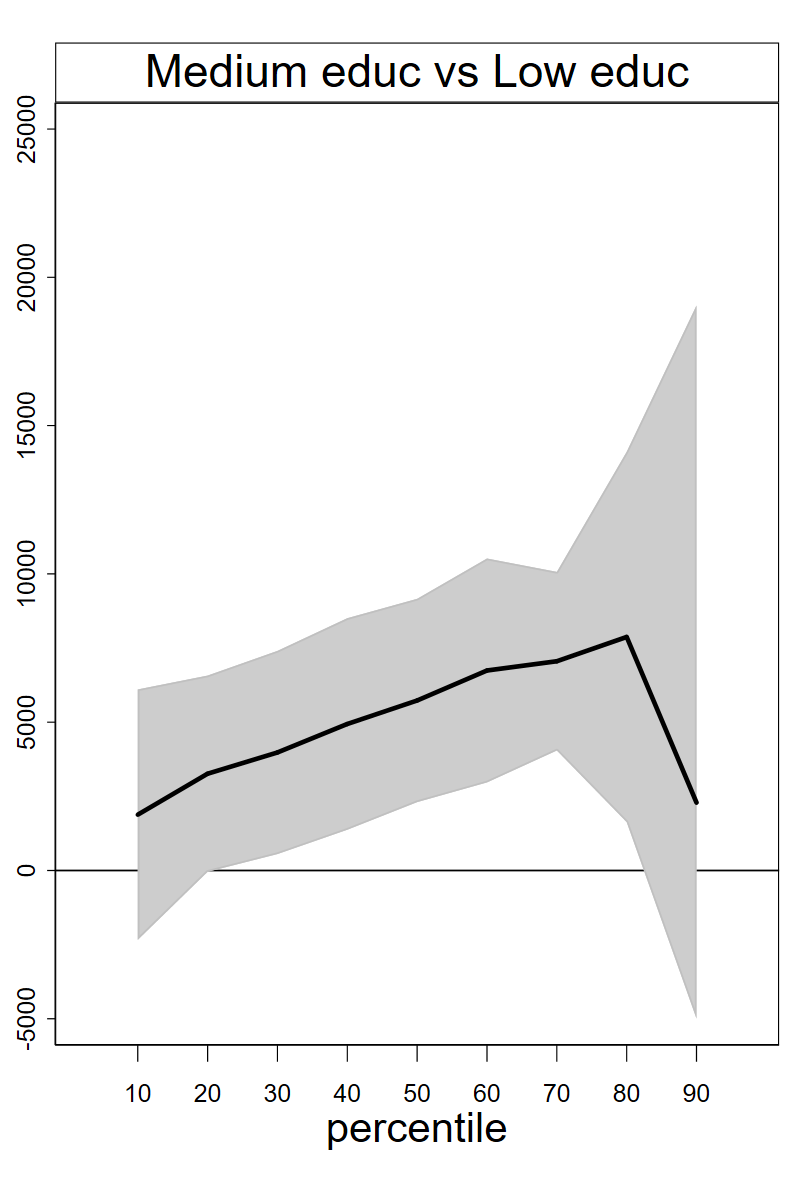

Supplement: Supplementary file 1 [file mmc1.zip › Data_in_Brief/output/graphs/G_2_3_2011_17.png]

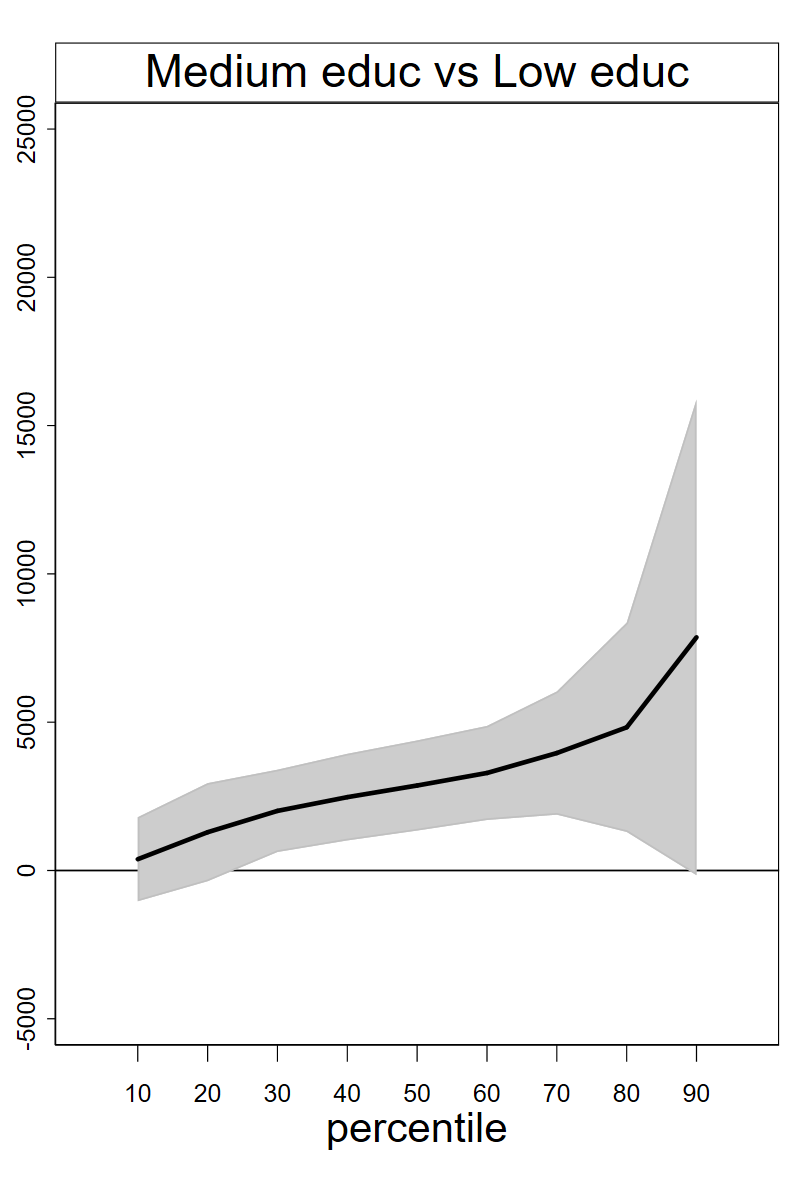

Supplement: Supplementary file 1 [file mmc1.zip › Data_in_Brief/output/graphs/G_2_3_2011_19.png]

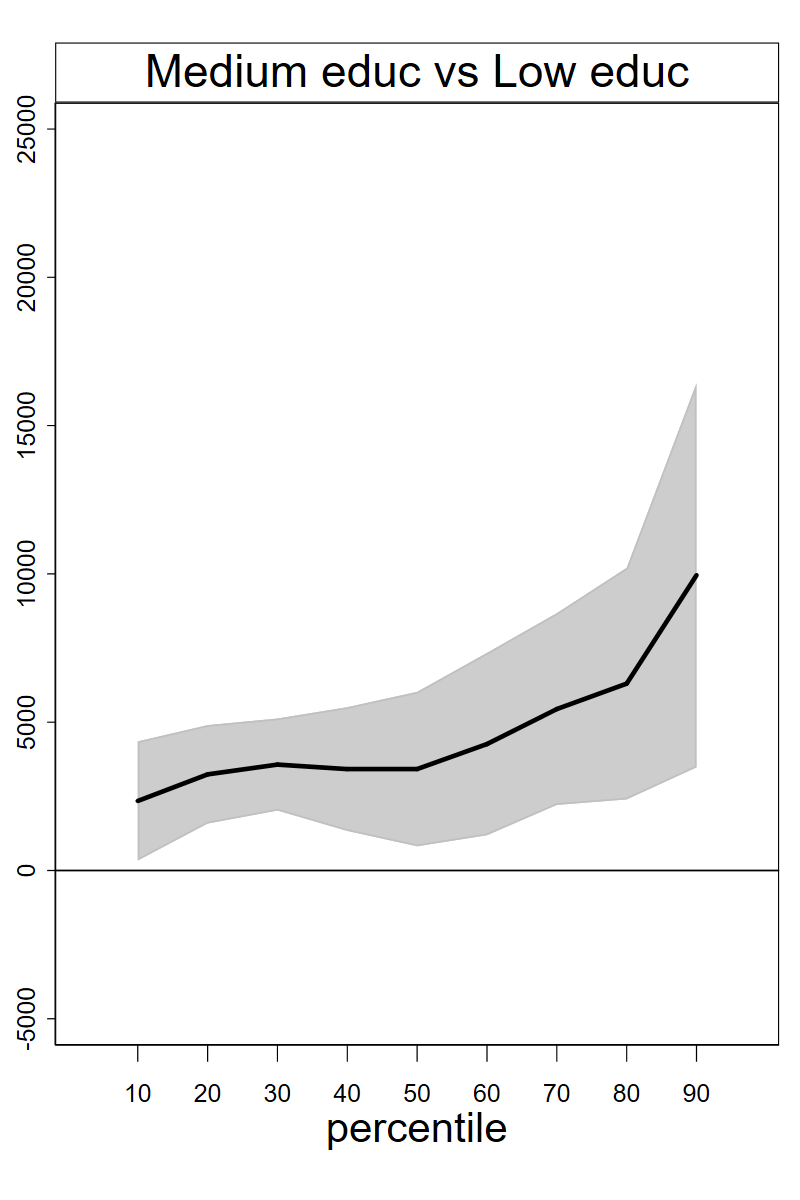

Supplement: Supplementary file 1 [file mmc1.zip › Data_in_Brief/output/graphs/G_2_3_2011_2.png]

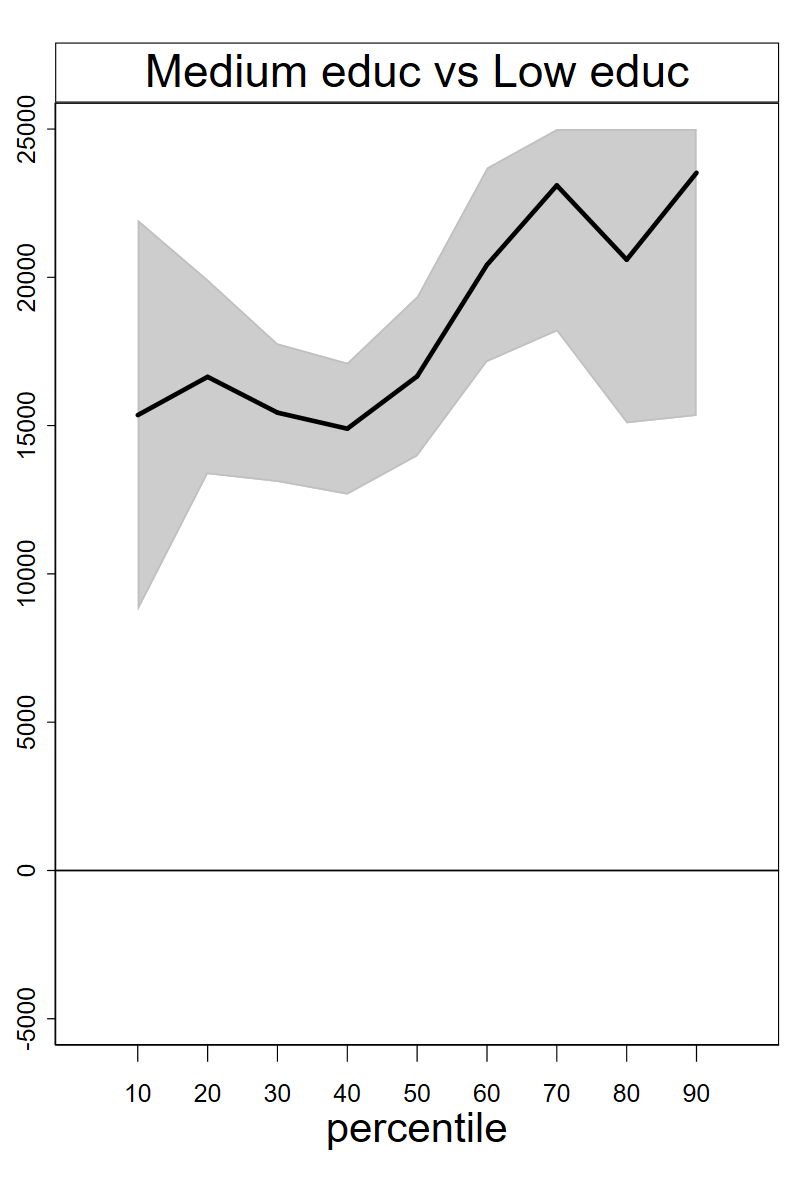

Supplement: Supplementary file 1 [file mmc1.zip › Data_in_Brief/output/graphs/G_2_3_2011_20.png]

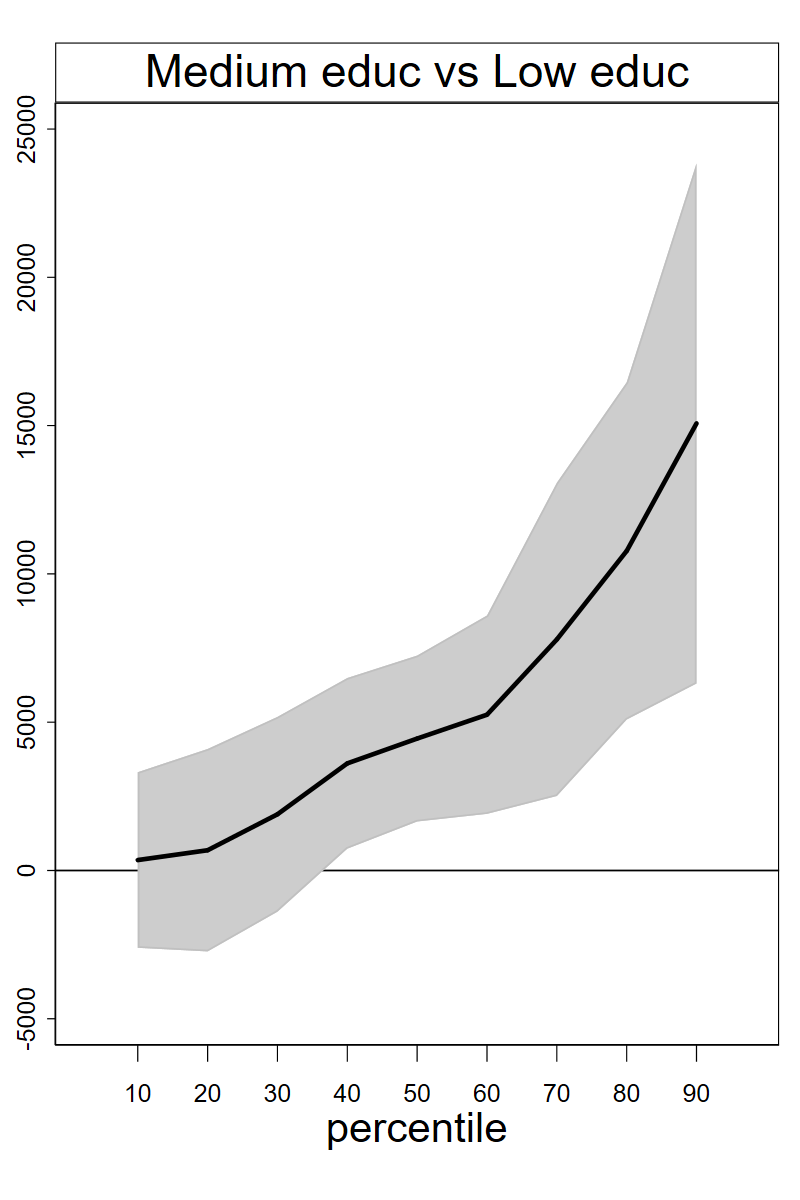

Supplement: Supplementary file 1 [file mmc1.zip › Data_in_Brief/output/graphs/G_2_3_2011_23.png]

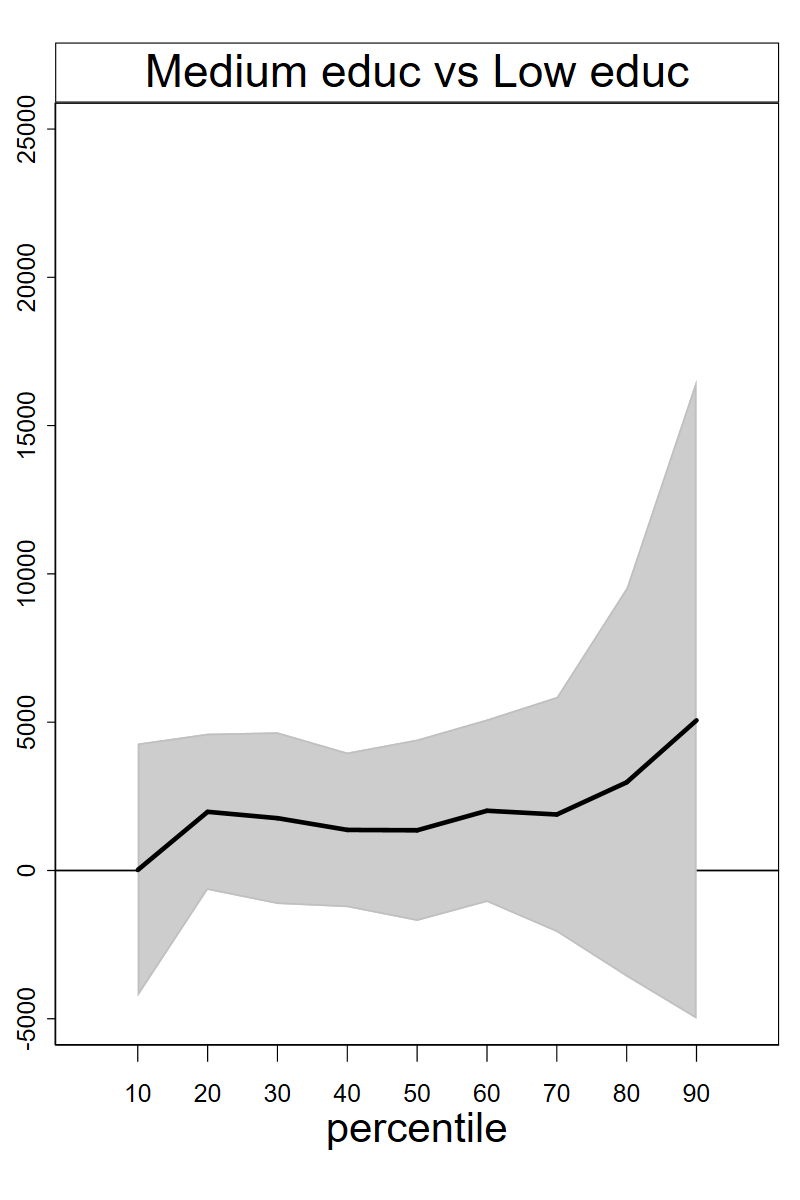

Supplement: Supplementary file 1 [file mmc1.zip › Data_in_Brief/output/graphs/G_2_3_2011_24.png]

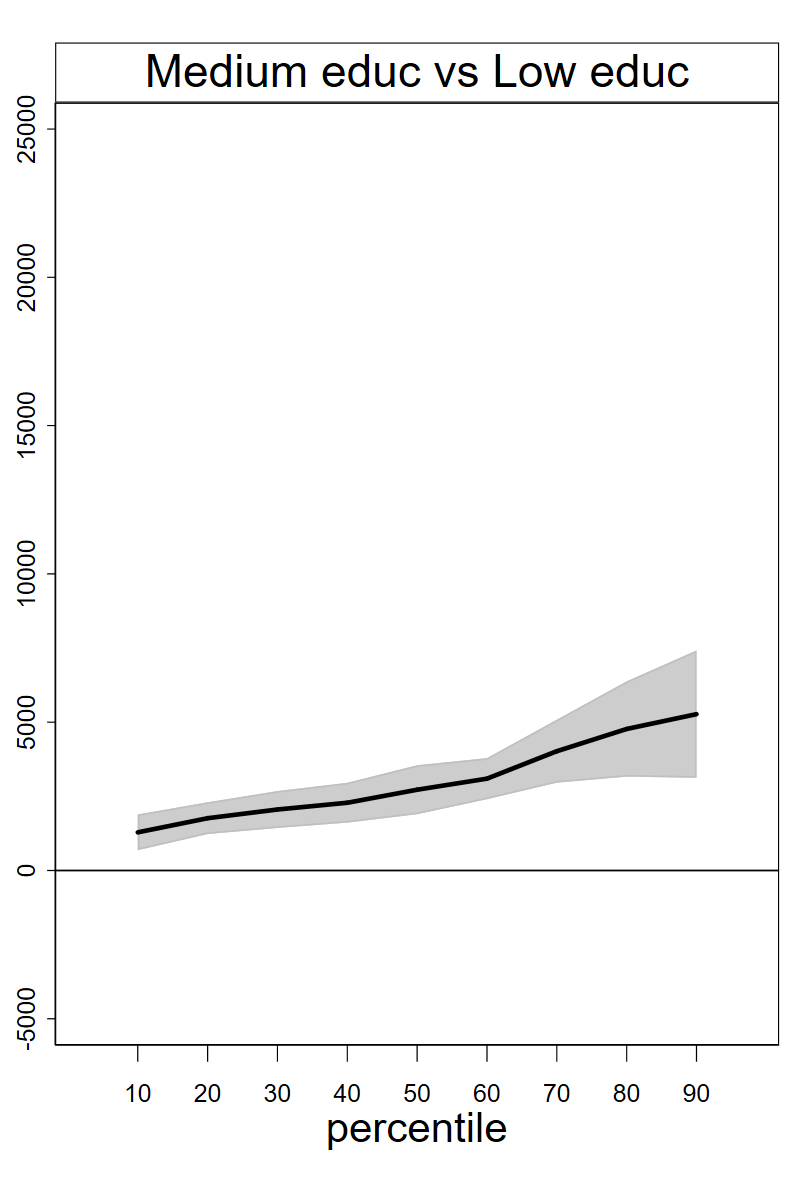

Supplement: Supplementary file 1 [file mmc1.zip › Data_in_Brief/output/graphs/G_2_3_2011_25.png]

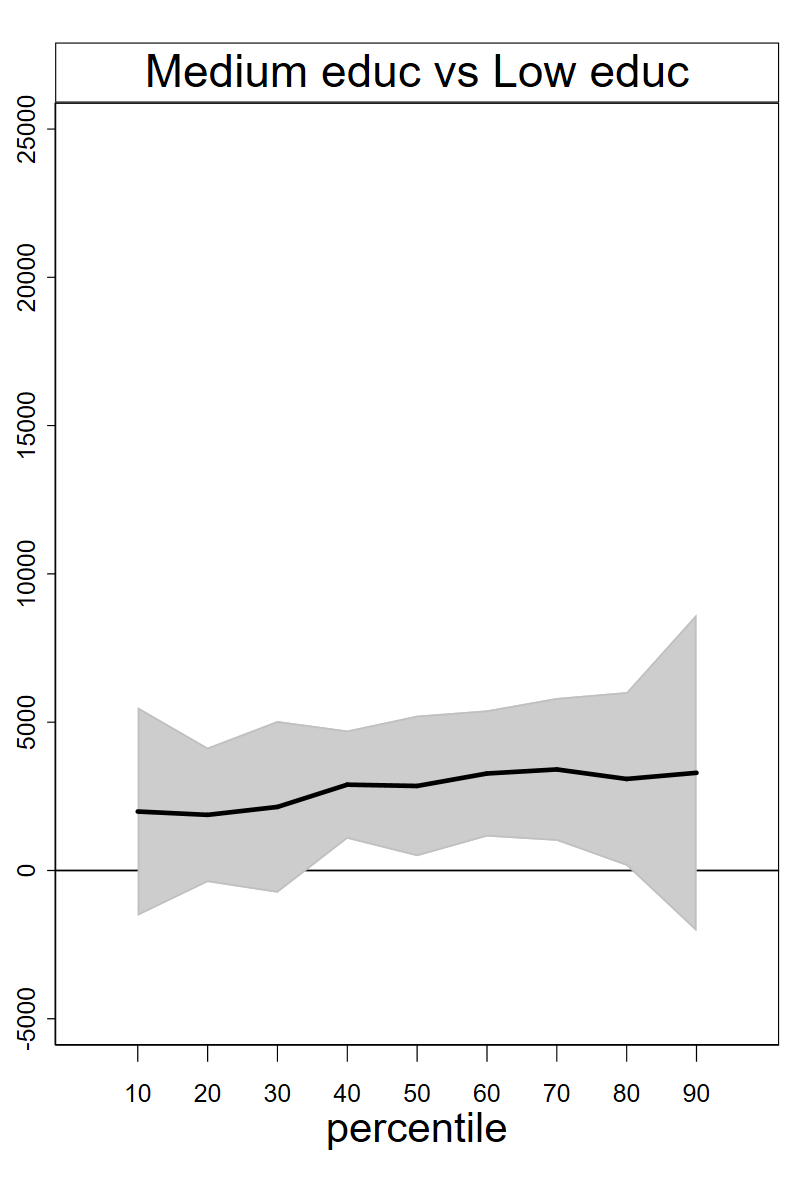

Supplement: Supplementary file 1 [file mmc1.zip › Data_in_Brief/output/graphs/G_2_3_2011_28.png]

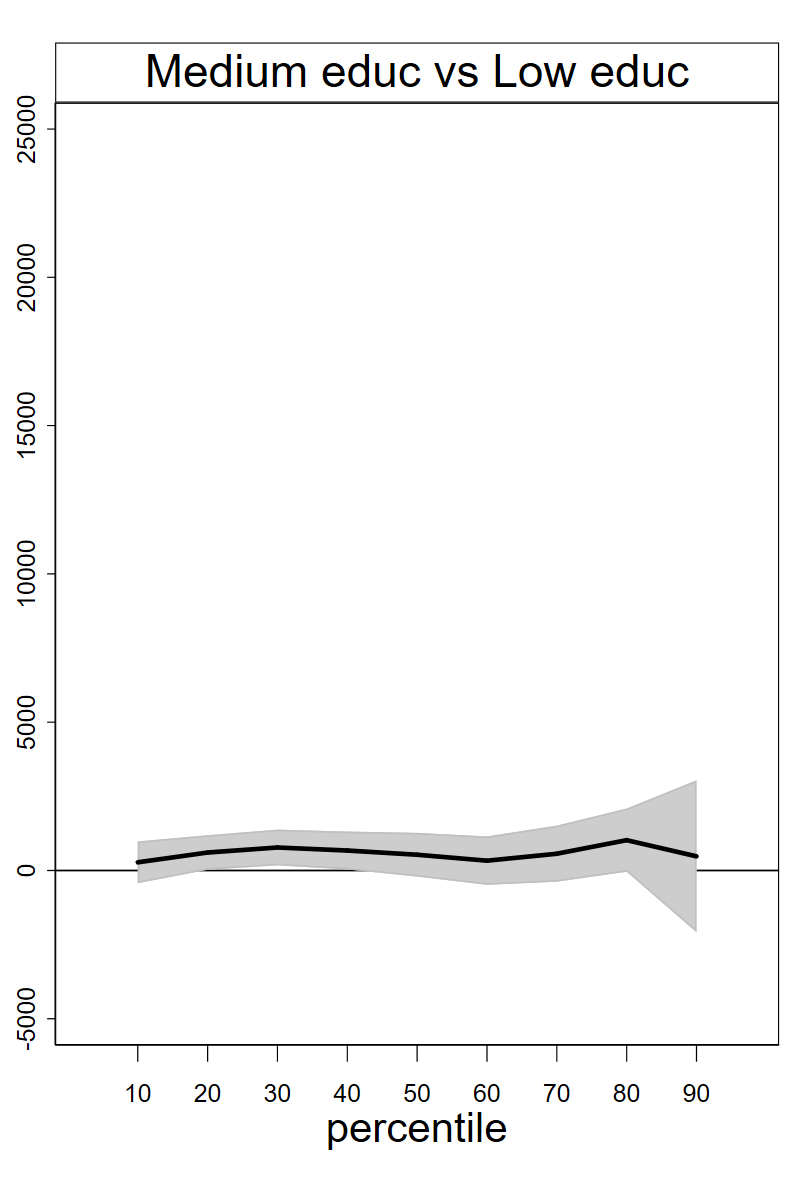

Supplement: Supplementary file 1 [file mmc1.zip › Data_in_Brief/output/graphs/G_2_3_2011_30.png]

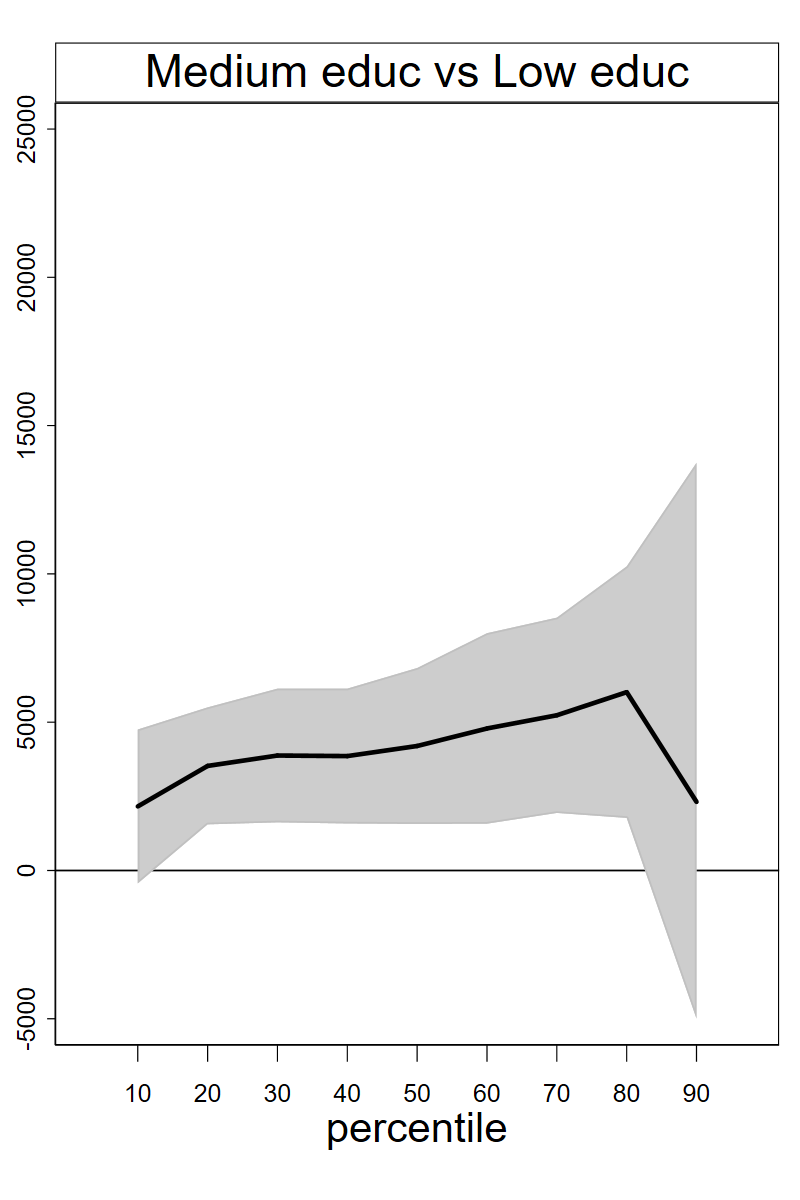

Supplement: Supplementary file 1 [file mmc1.zip › Data_in_Brief/output/graphs/G_2_3_2011_31.png]

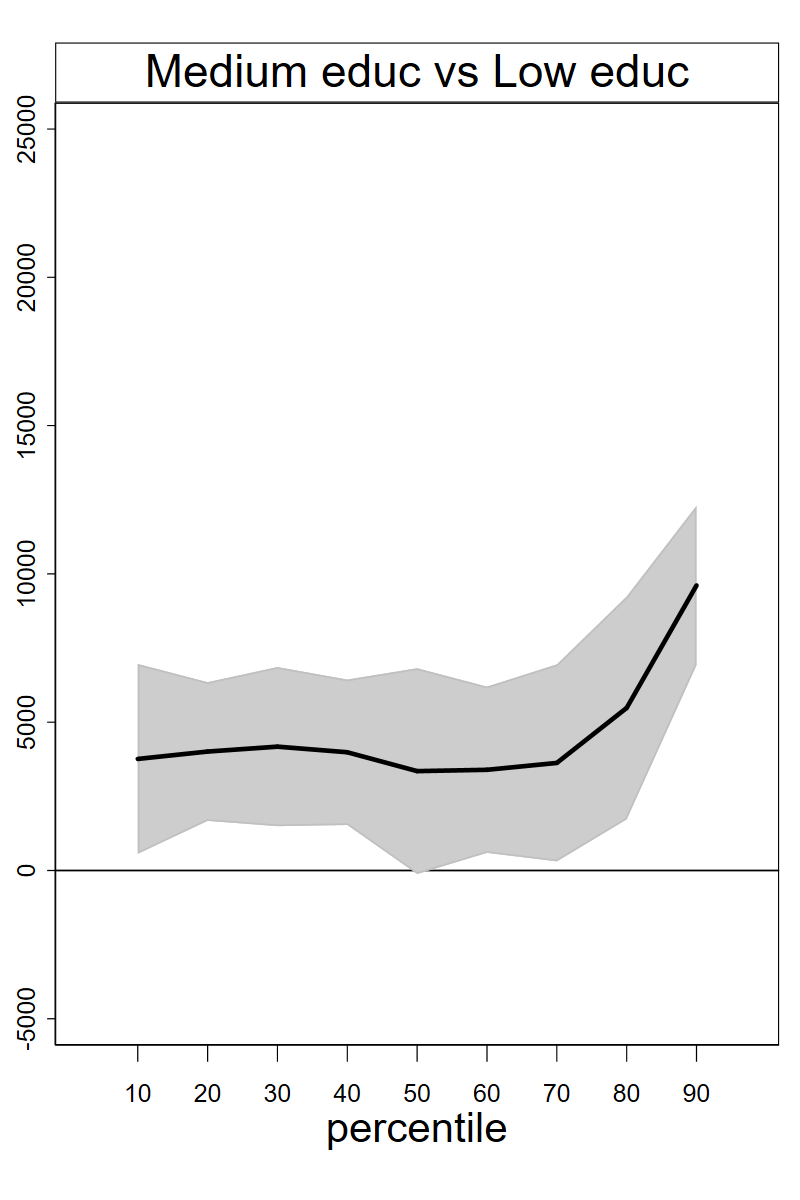

Supplement: Supplementary file 1 [file mmc1.zip › Data_in_Brief/output/graphs/G_2_3_2011_7.png]

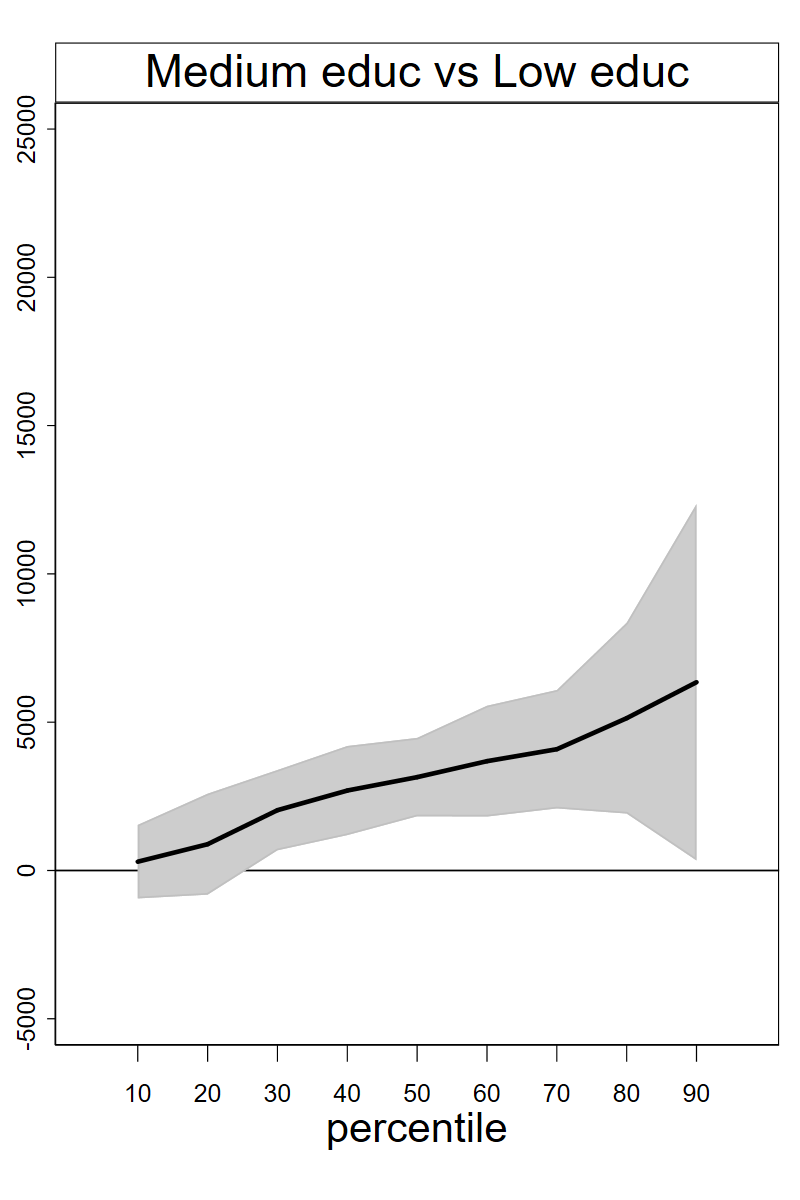

Supplement: Supplementary file 1 [file mmc1.zip › Data_in_Brief/output/graphs/G_2_3_2011_9.png]

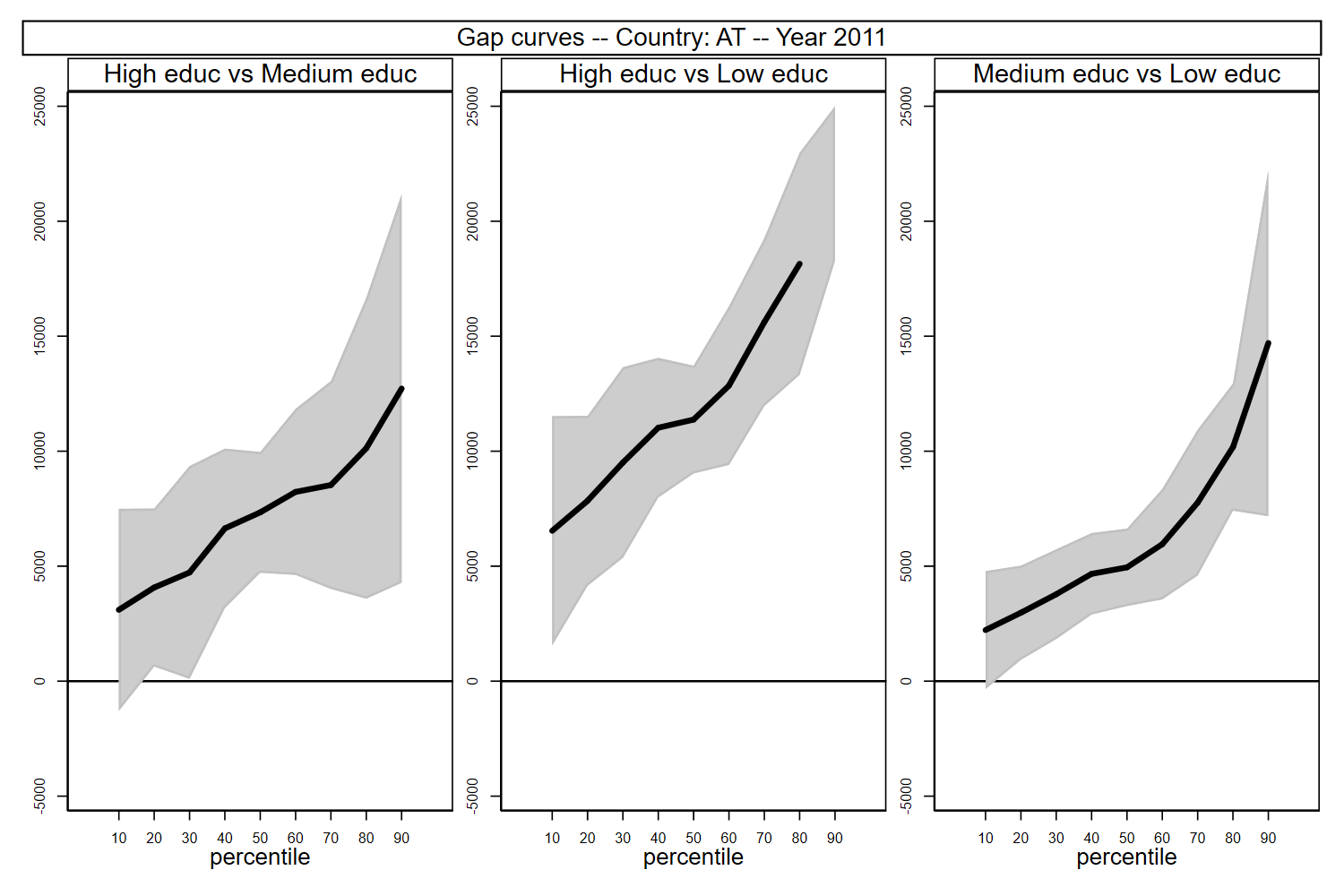

Supplement: Supplementary file 1 [file mmc1.zip › Data_in_Brief/output/graphs/gdom_1.png]

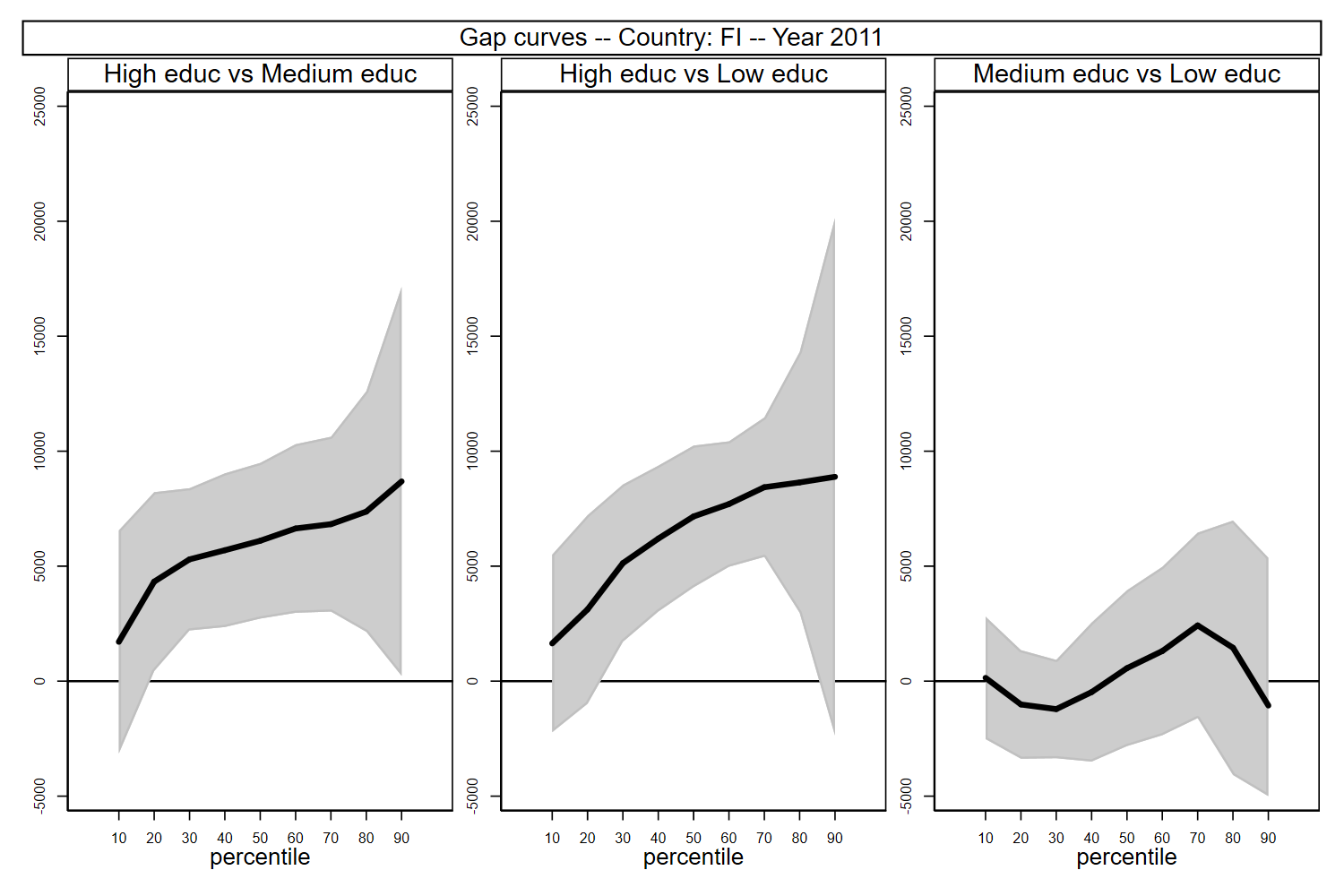

Supplement: Supplementary file 1 [file mmc1.zip › Data_in_Brief/output/graphs/gdom_12.png]

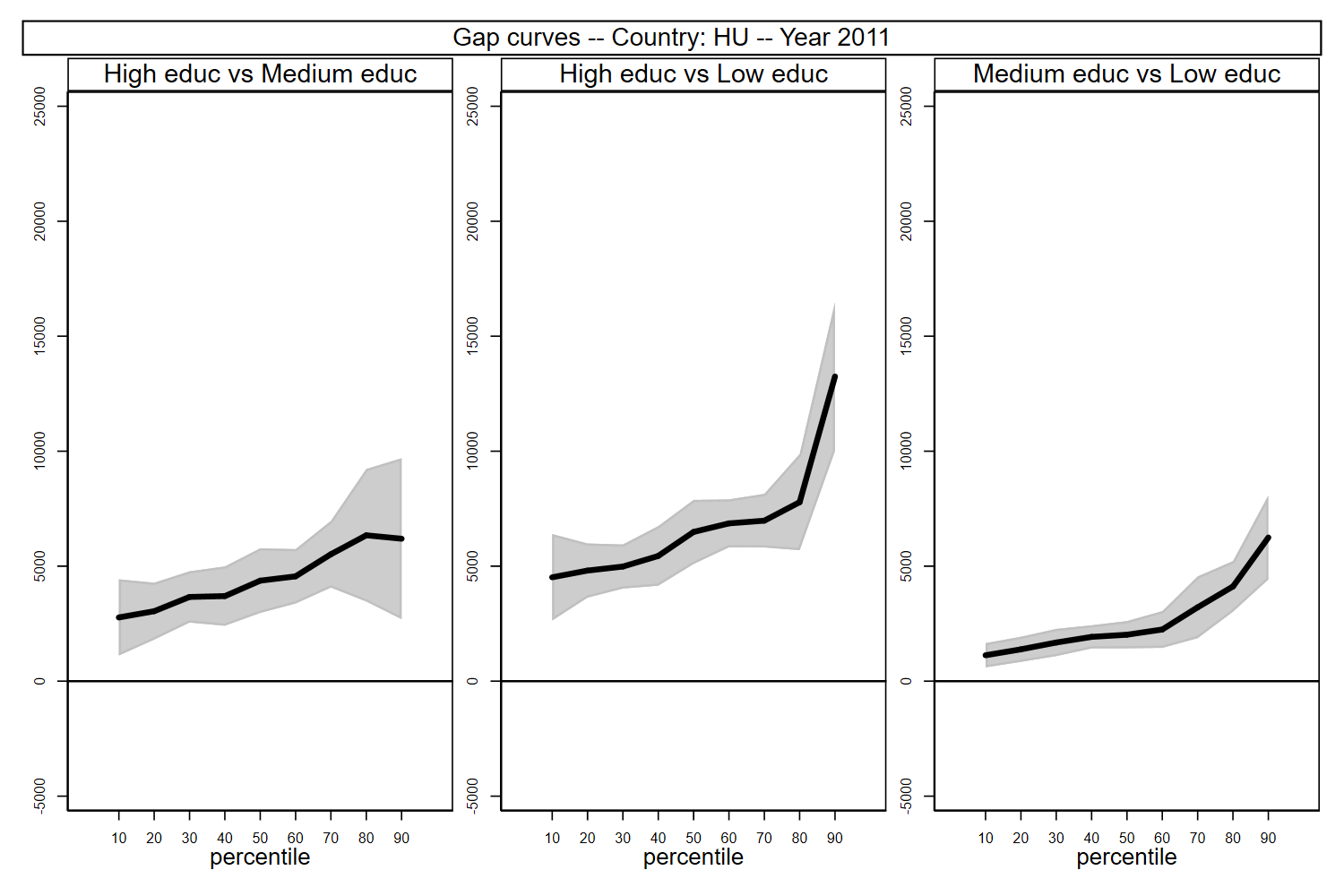

Supplement: Supplementary file 1 [file mmc1.zip › Data_in_Brief/output/graphs/gdom_15.png]

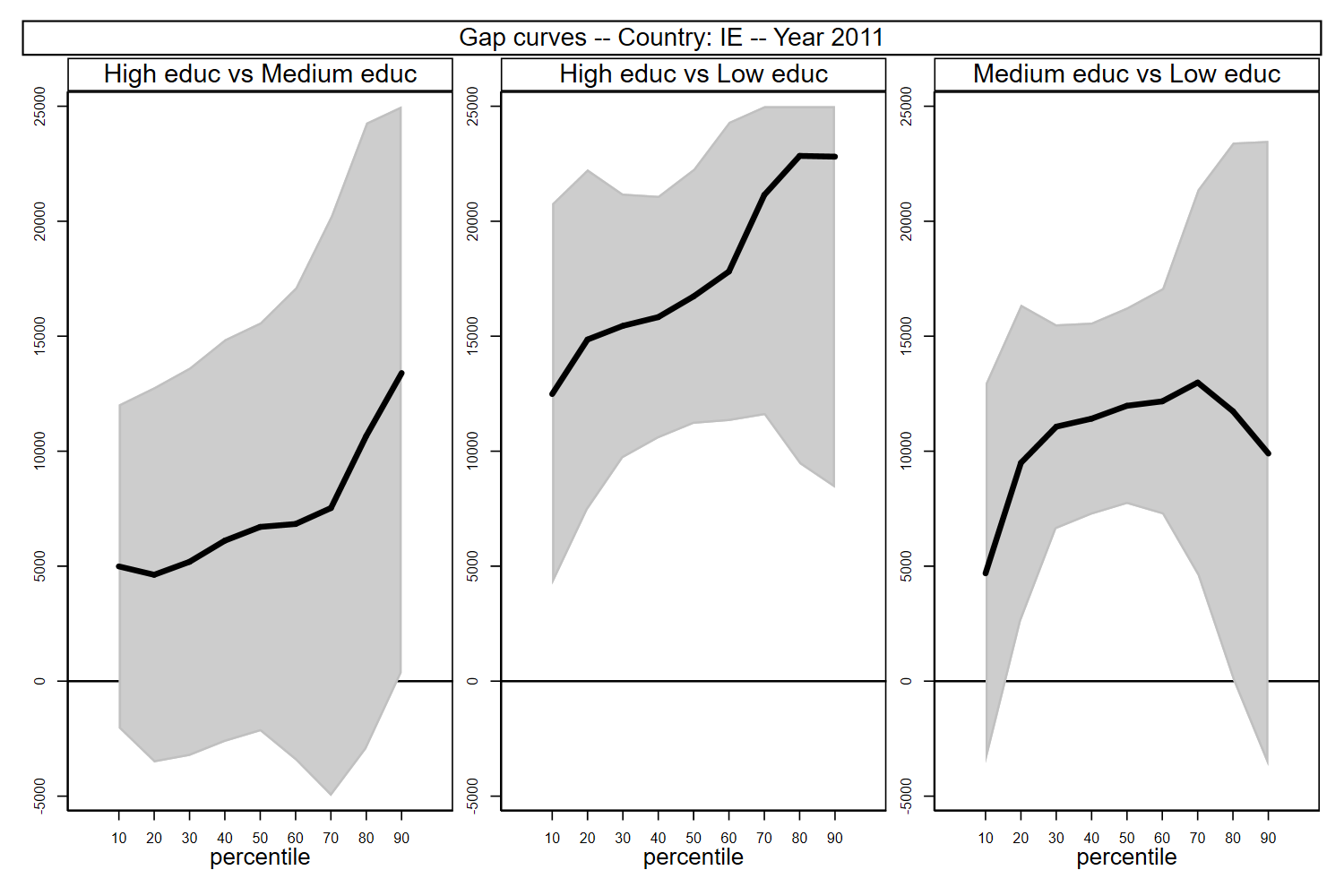

Supplement: Supplementary file 1 [file mmc1.zip › Data_in_Brief/output/graphs/gdom_16.png]

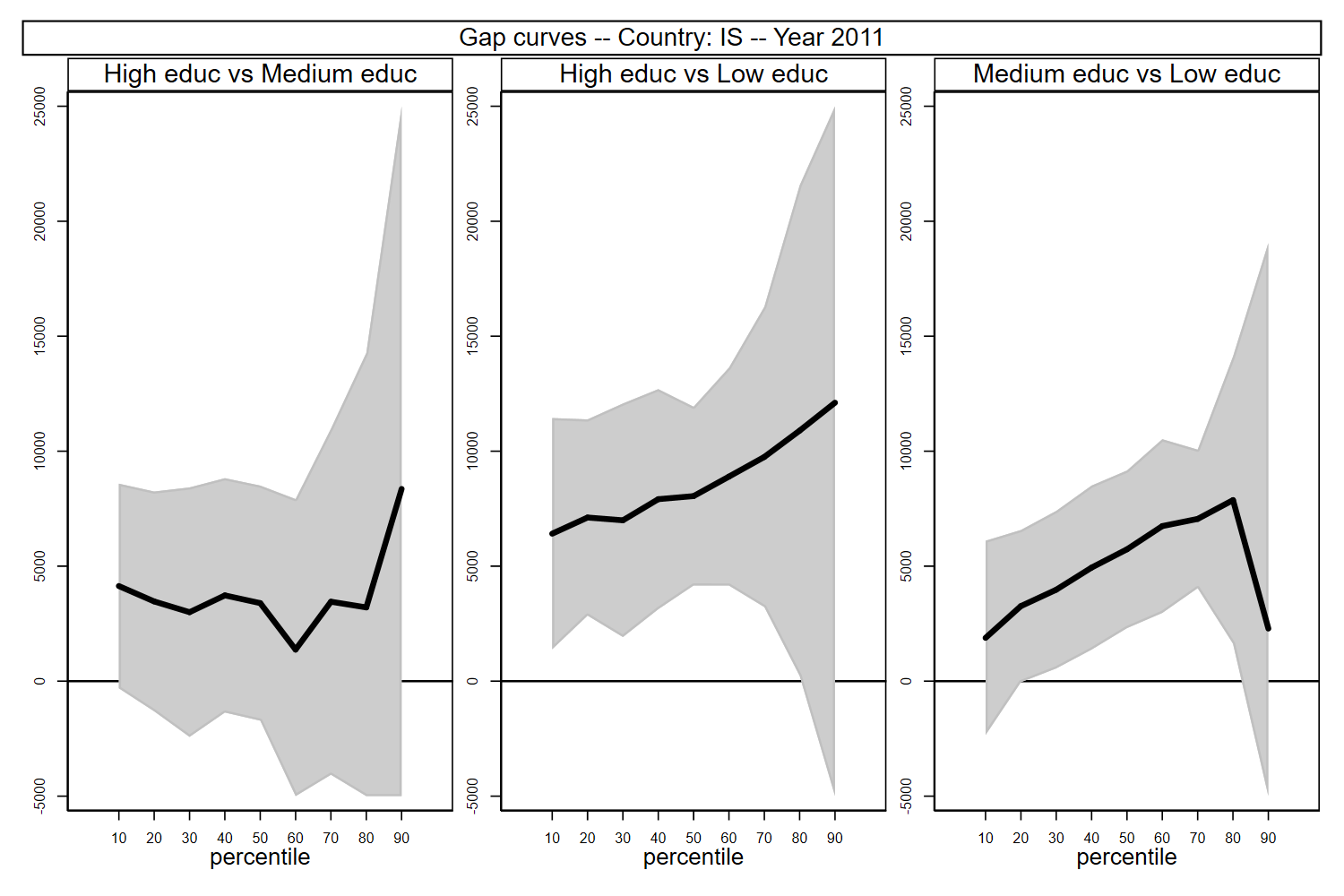

Supplement: Supplementary file 1 [file mmc1.zip › Data_in_Brief/output/graphs/gdom_17.png]

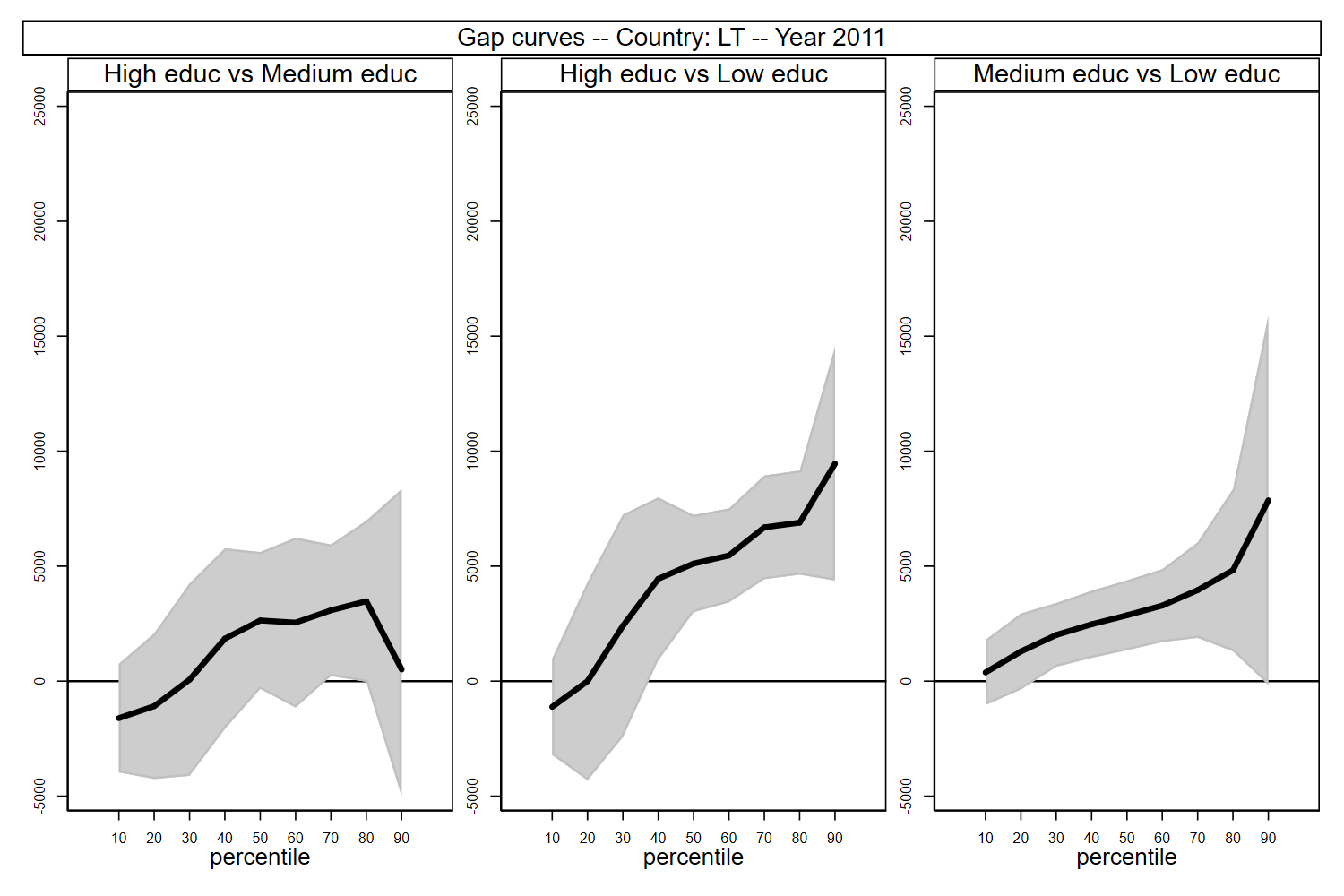

Supplement: Supplementary file 1 [file mmc1.zip › Data_in_Brief/output/graphs/gdom_19.png]

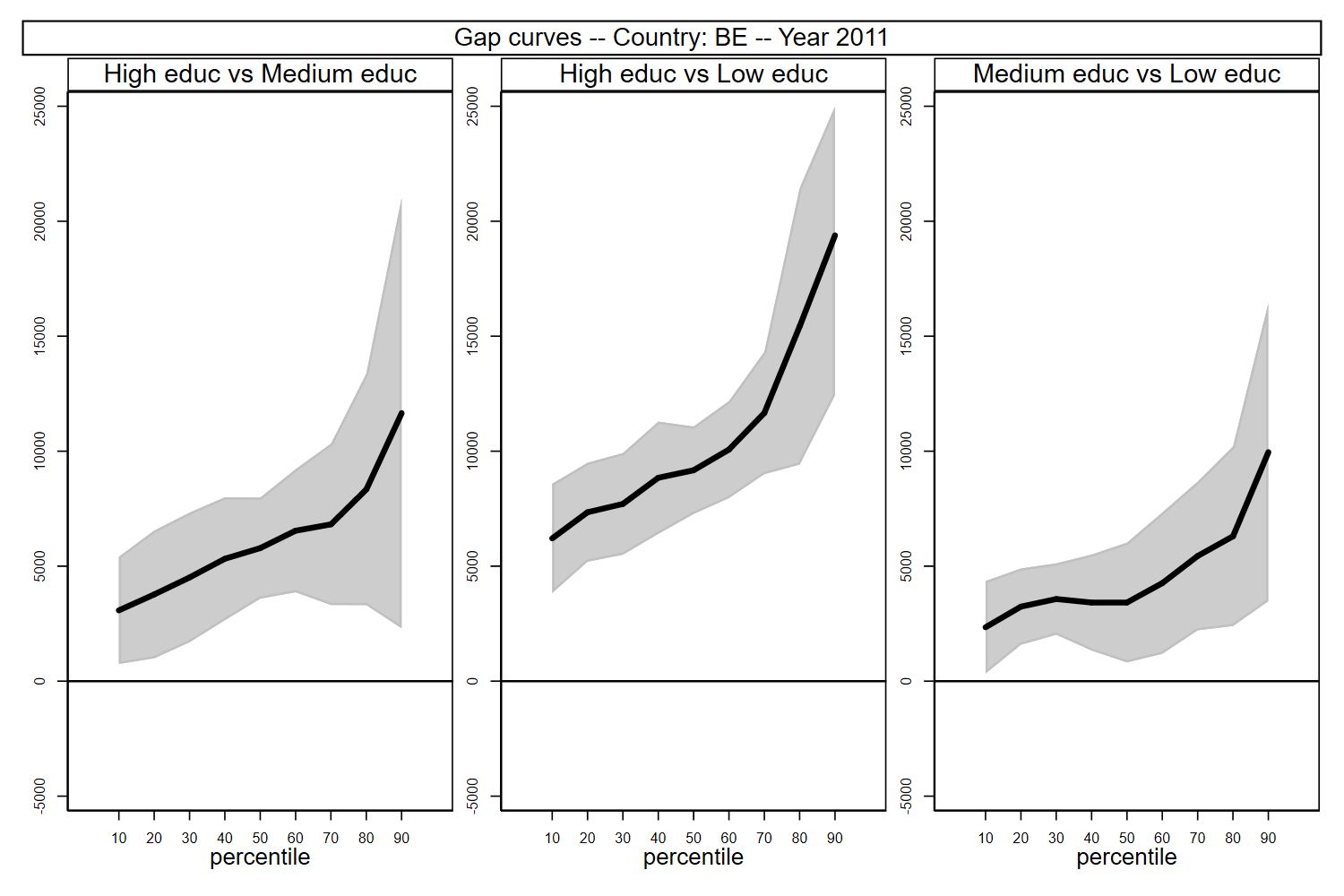

Supplement: Supplementary file 1 [file mmc1.zip › Data_in_Brief/output/graphs/gdom_2.png]

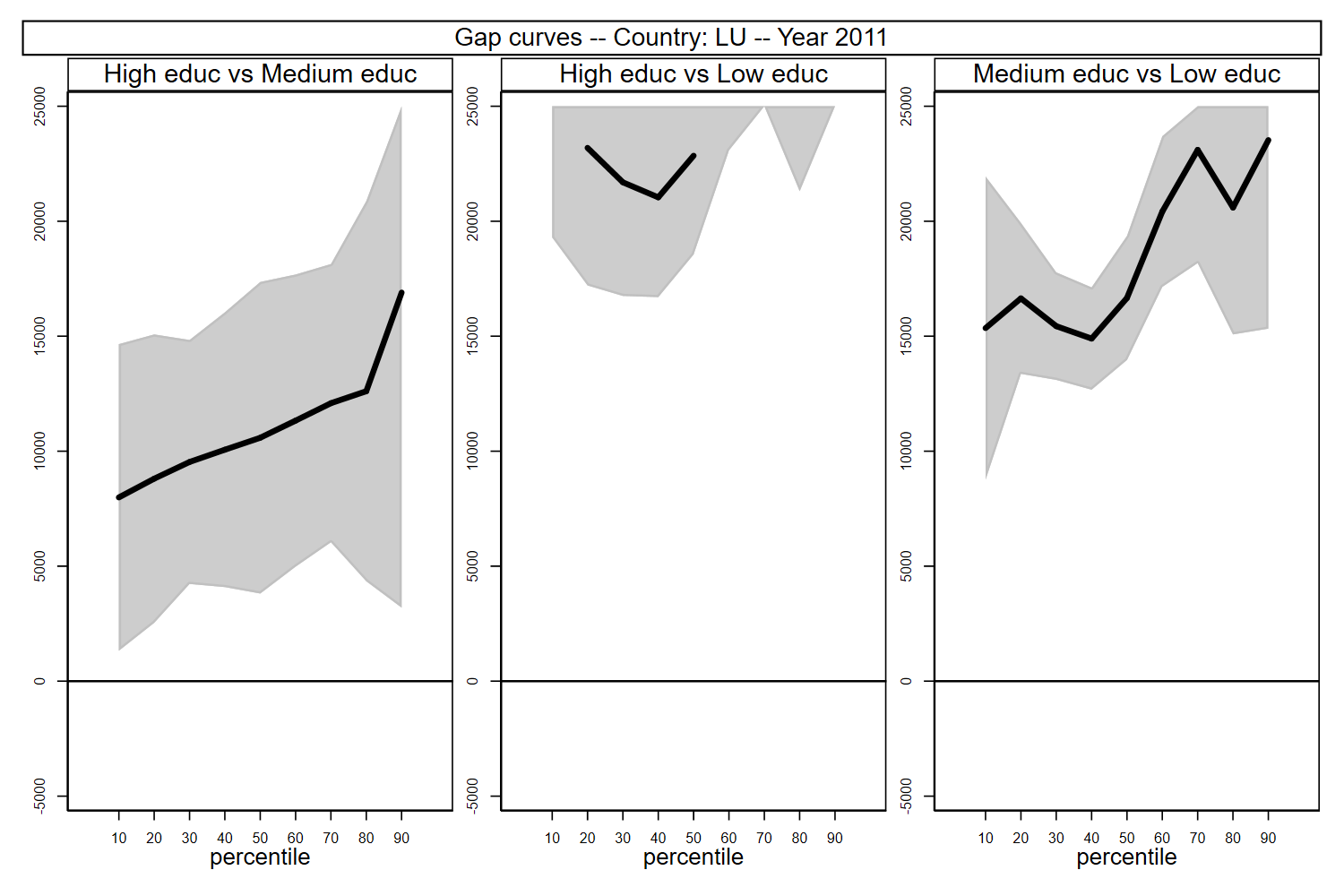

Supplement: Supplementary file 1 [file mmc1.zip › Data_in_Brief/output/graphs/gdom_20.png]

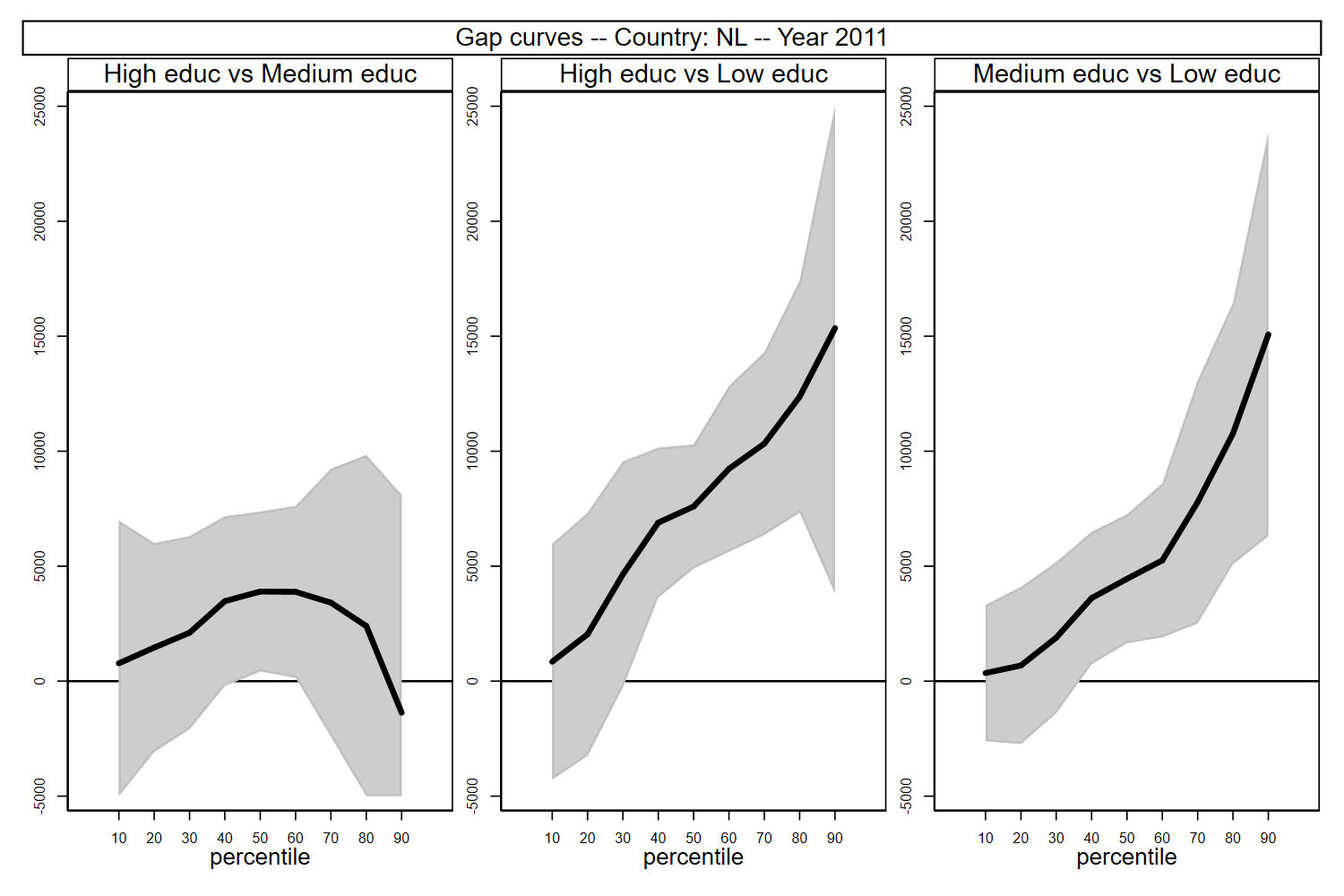

Supplement: Supplementary file 1 [file mmc1.zip › Data_in_Brief/output/graphs/gdom_23.png]

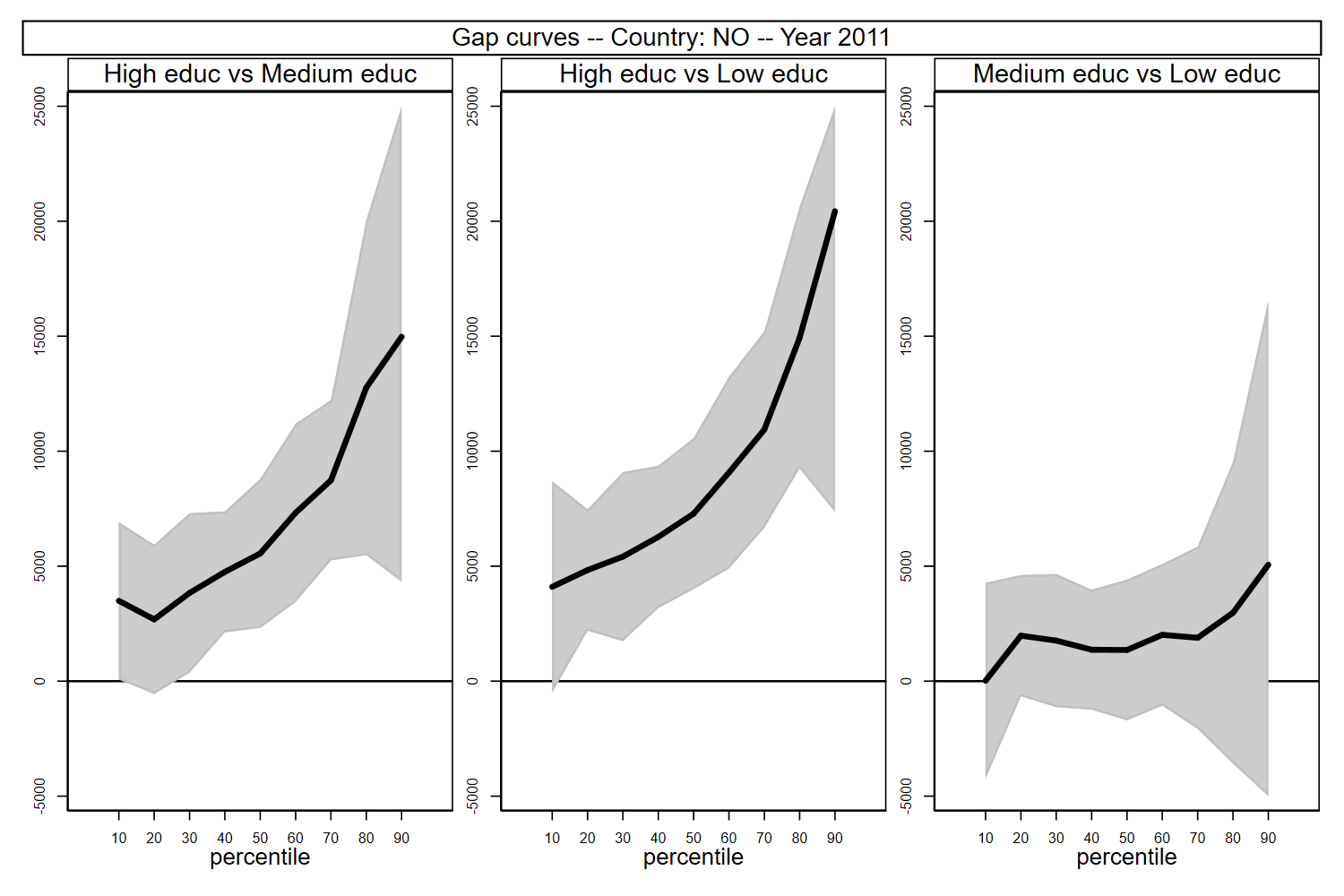

Supplement: Supplementary file 1 [file mmc1.zip › Data_in_Brief/output/graphs/gdom_24.png]

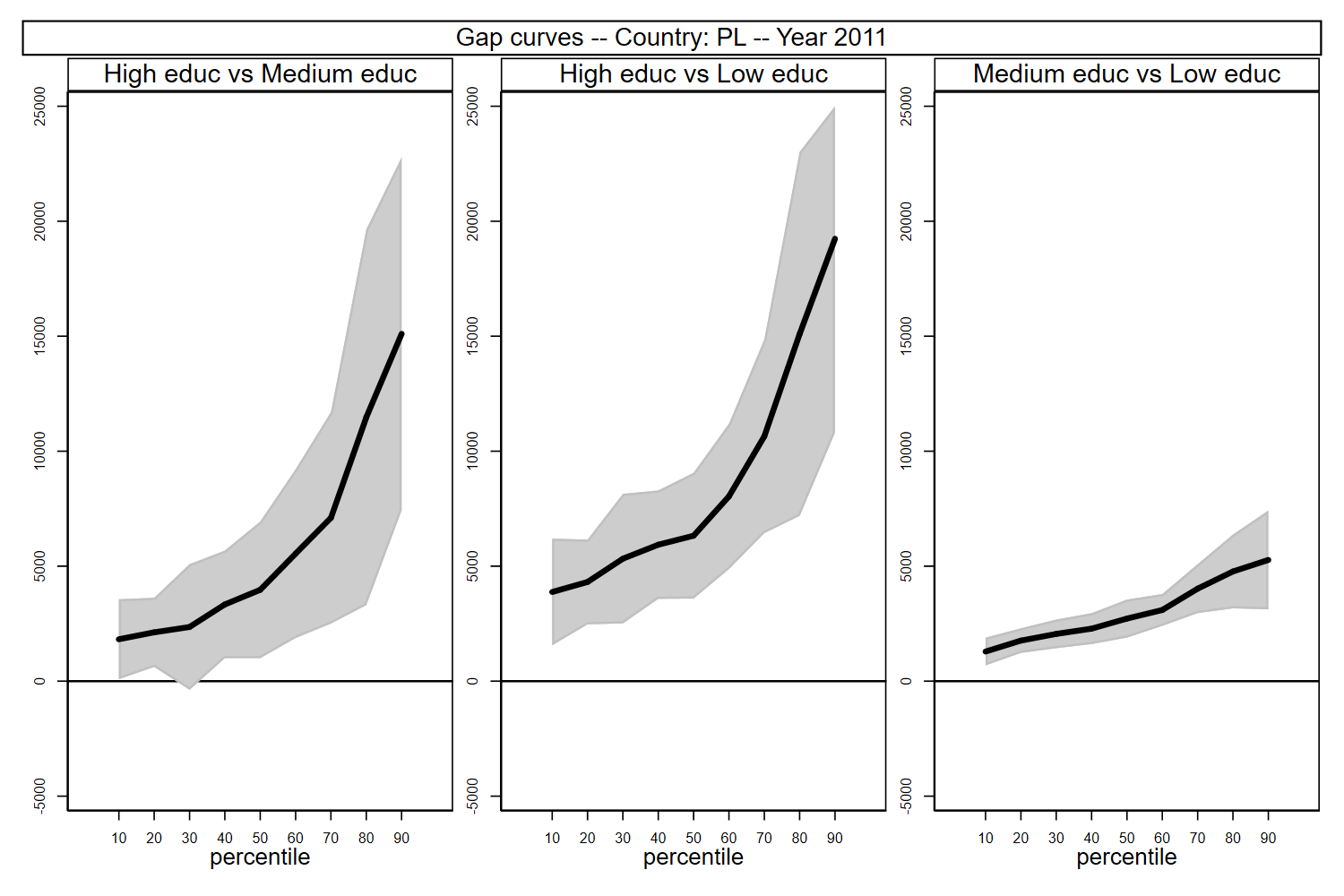

Supplement: Supplementary file 1 [file mmc1.zip › Data_in_Brief/output/graphs/gdom_25.png]

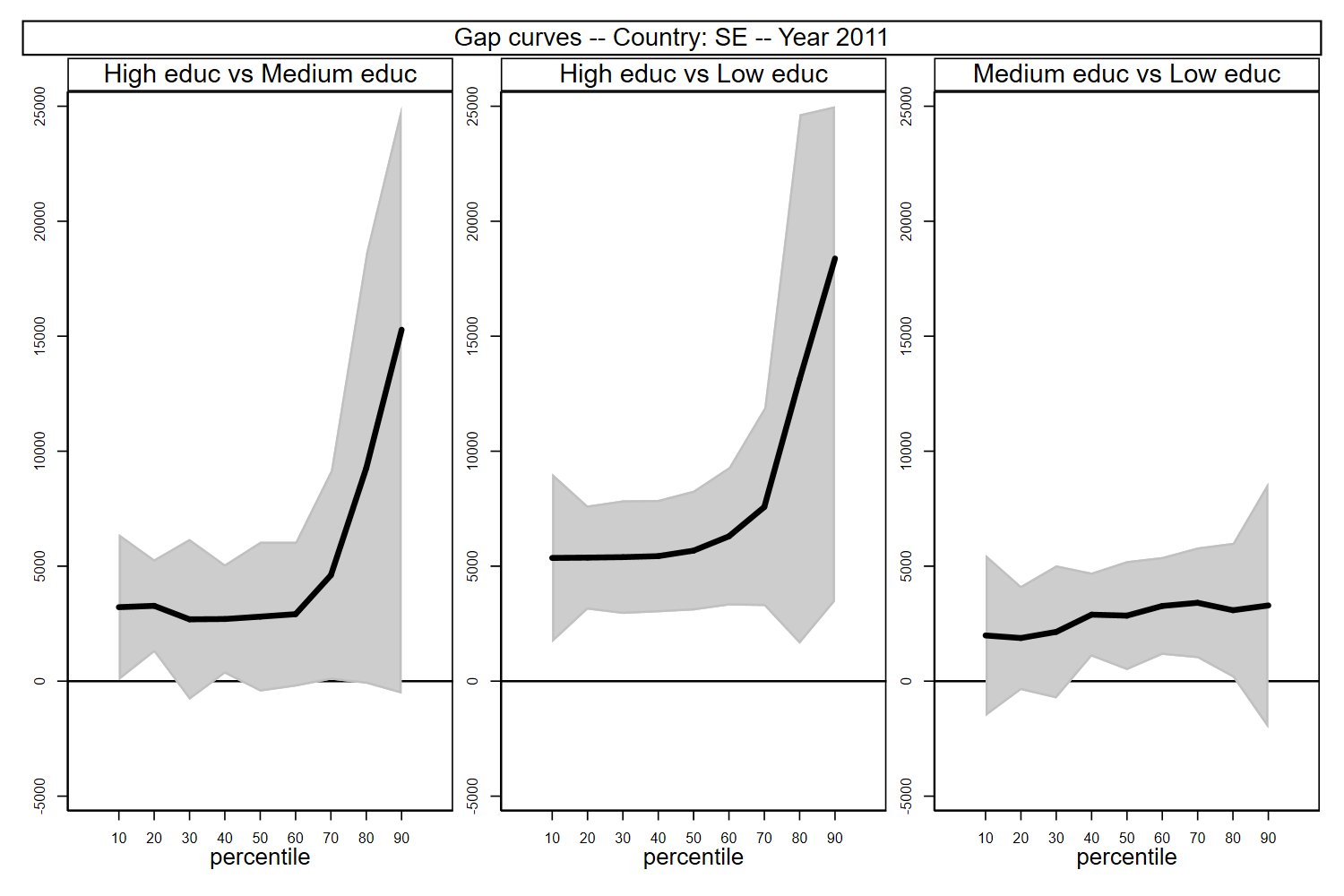

Supplement: Supplementary file 1 [file mmc1.zip › Data_in_Brief/output/graphs/gdom_28.png]

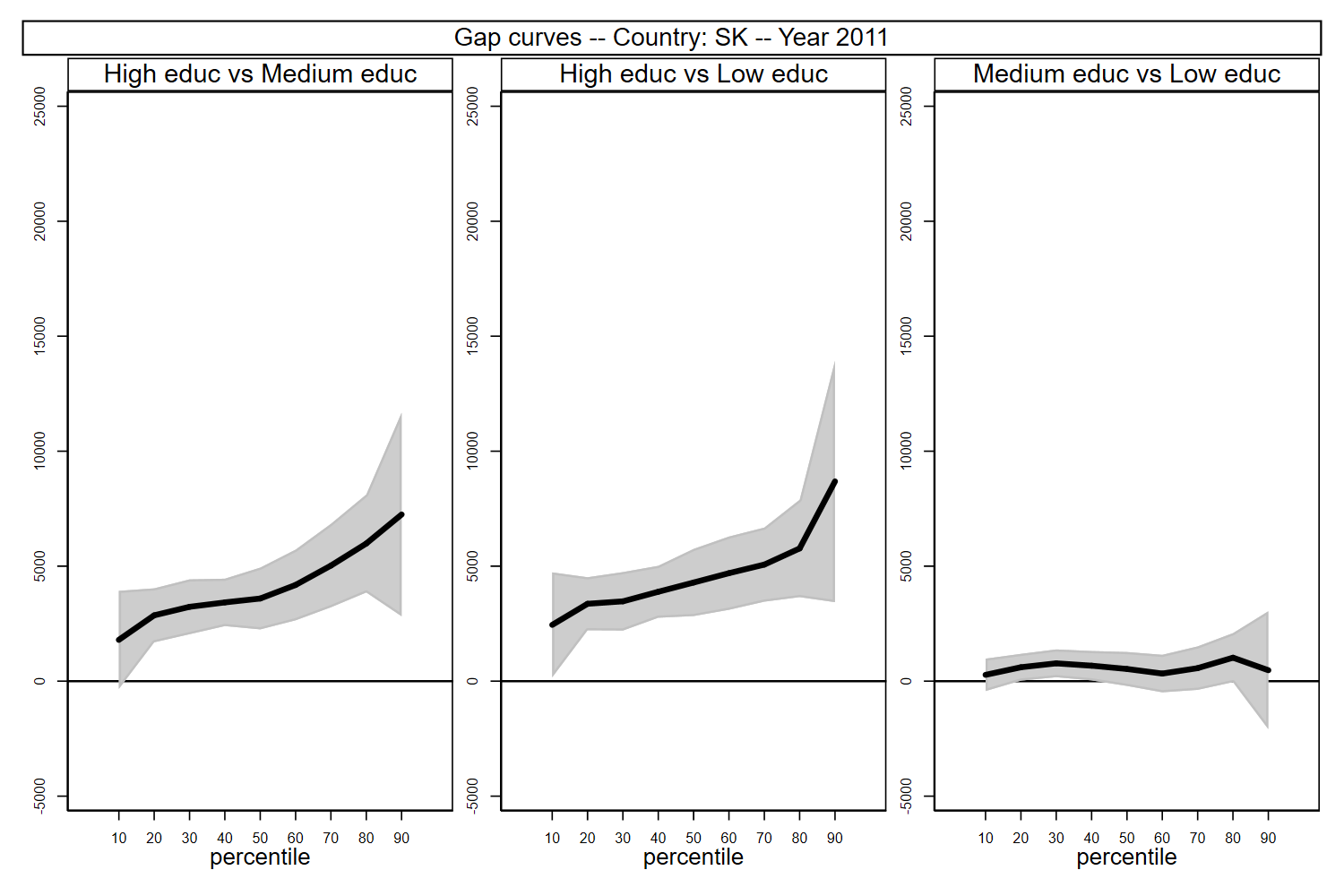

Supplement: Supplementary file 1 [file mmc1.zip › Data_in_Brief/output/graphs/gdom_30.png]

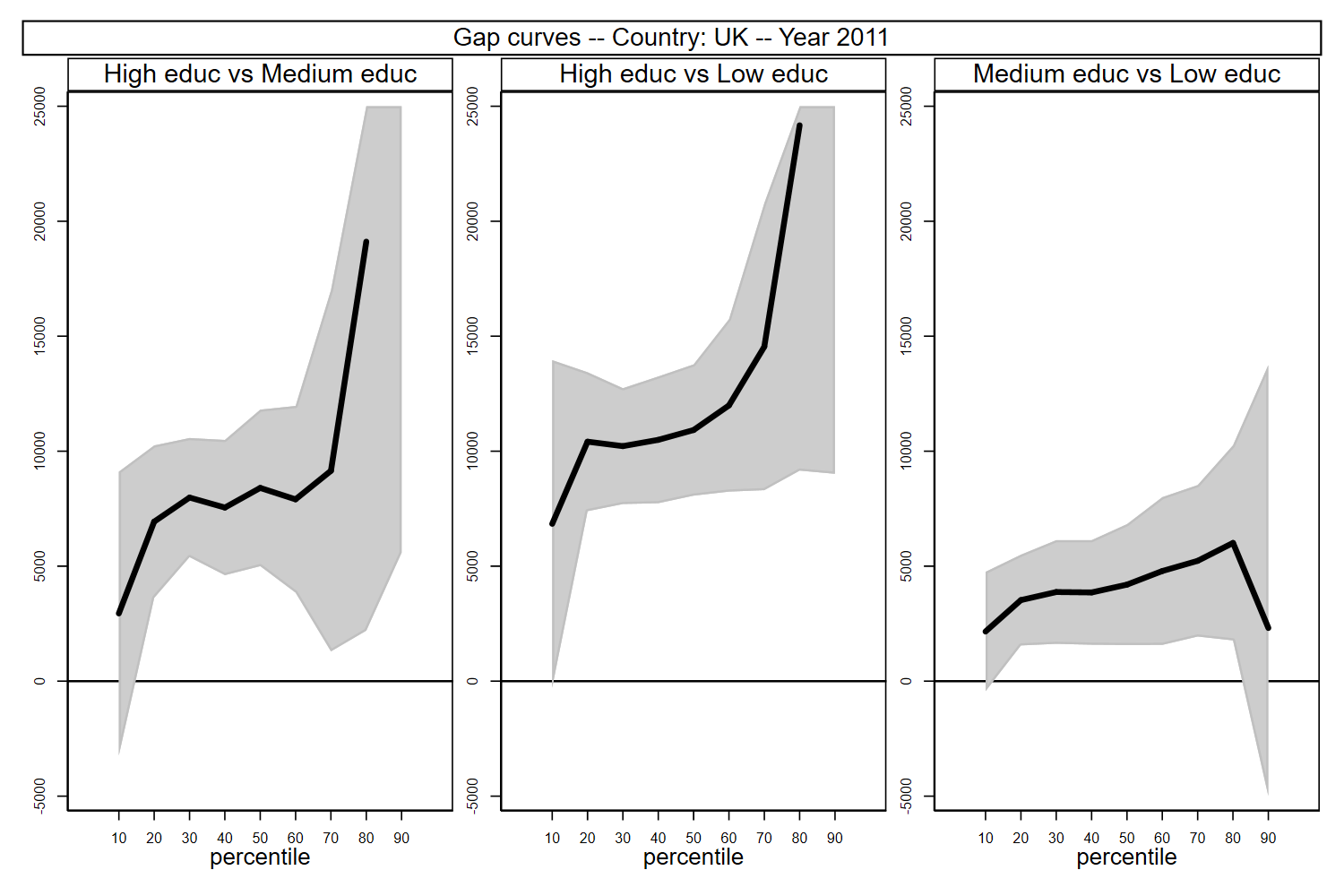

Supplement: Supplementary file 1 [file mmc1.zip › Data_in_Brief/output/graphs/gdom_31.png]

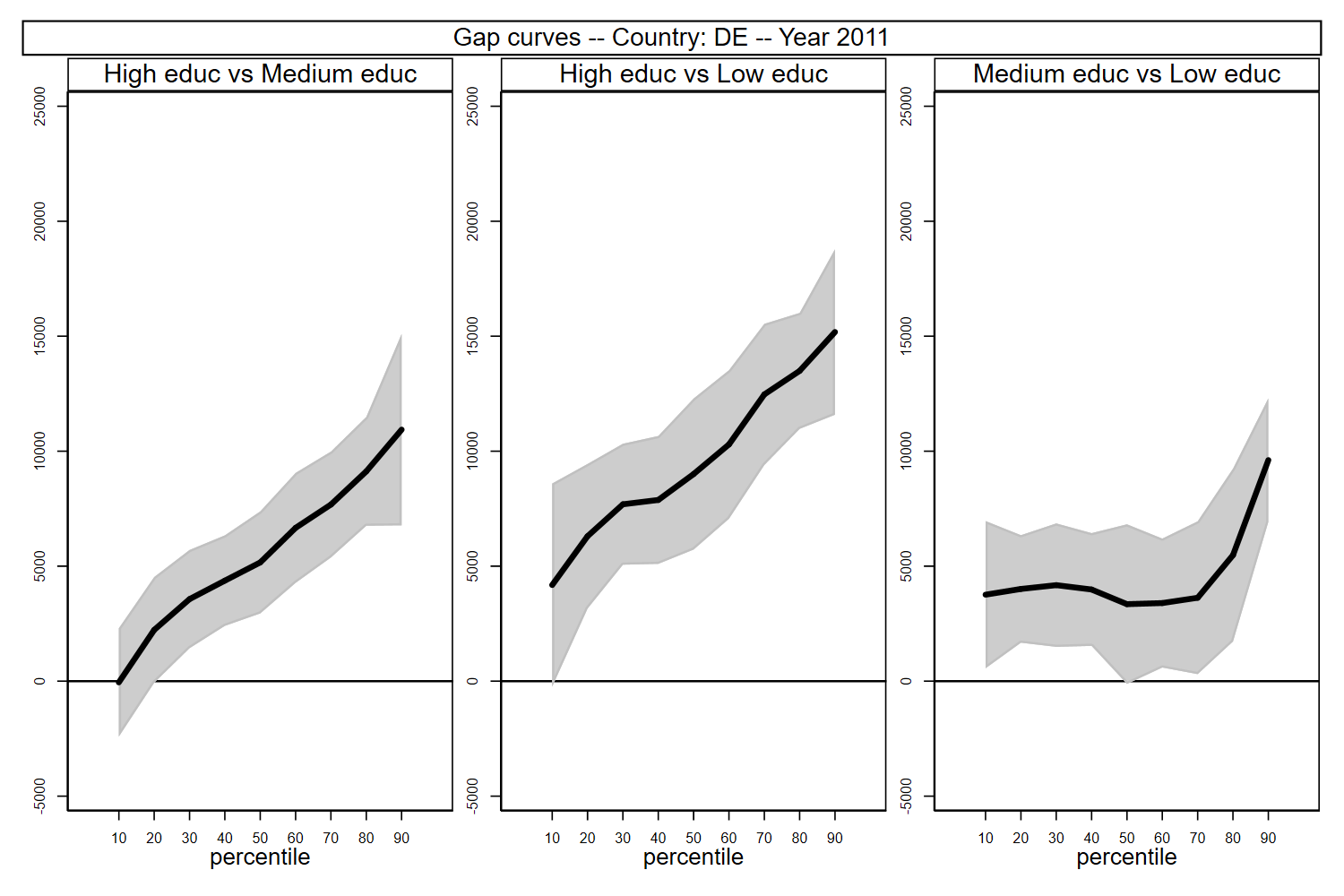

Supplement: Supplementary file 1 [file mmc1.zip › Data_in_Brief/output/graphs/gdom_7.png]

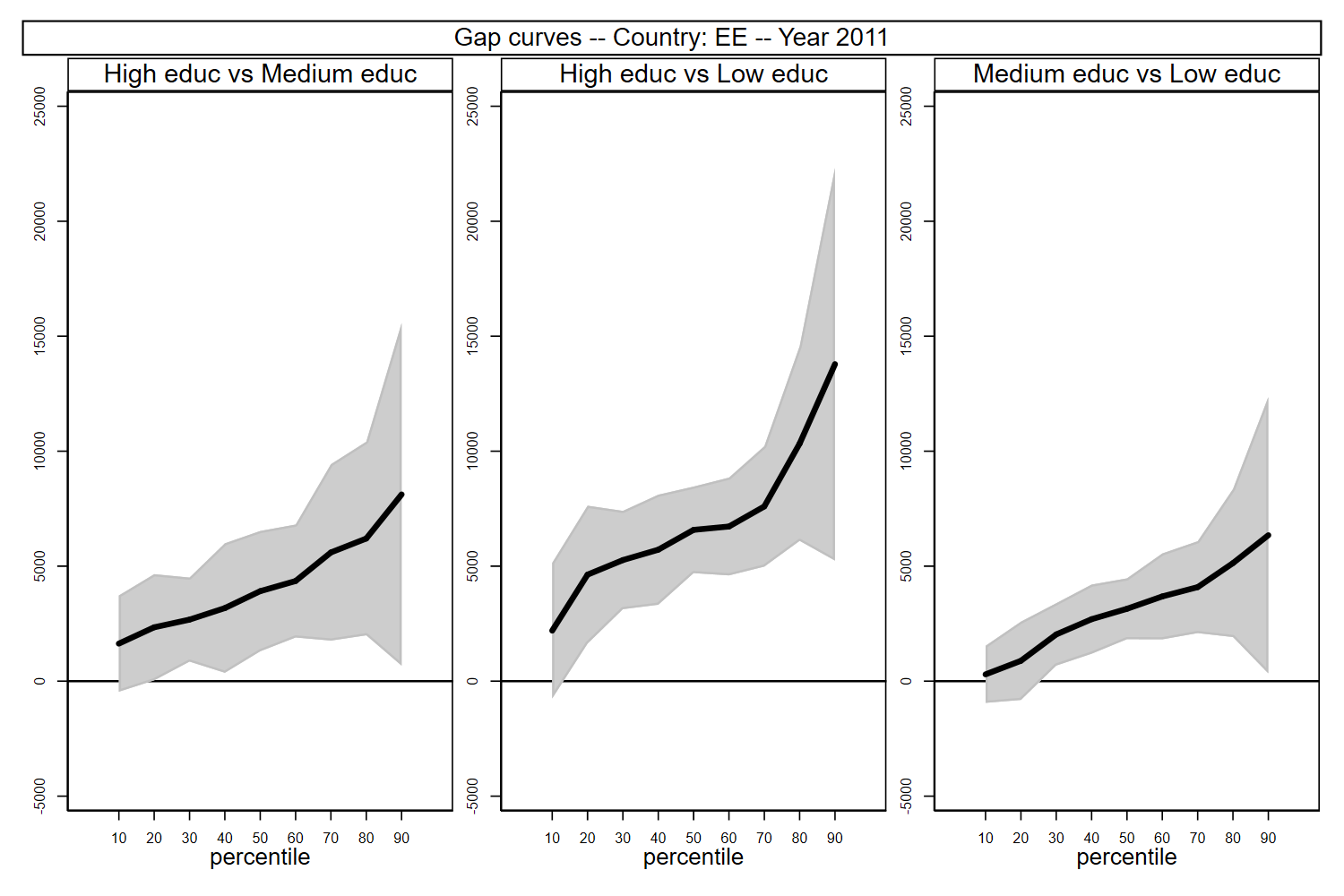

Supplement: Supplementary file 1 [file mmc1.zip › Data_in_Brief/output/graphs/gdom_9.png]
